# Mutations in histones dysregulate copper homeostasis leading to defect in Sec61-dependent protein translocation mechanism in *Saccharomyces cerevisiae*

**DOI:** 10.1016/j.jbc.2025.108163

**Published:** 2025-01-08

**Authors:** Santoshi Acharjee, Rajshree Pal, Smriti Anand, Prateeksha Thakur, Vandana Anjana, Ranu Singh, Mrittika Paul, Ashis Biswas, Raghuvir Singh Tomar

**Affiliations:** 1 Department of Biological Sciences, Indian Institute of Science Education and Research Bhopal, Bhopal, Madhya Pradesh, India; 2 Department of Earth and Environmental Sciences, Indian Institute of Science Education and Research Bhopal, Bhopal, Madhya Pradesh, India

**Keywords:** Sec61, endoplasmic reticulum, copper homeostasis, protein translocation

## Abstract

The translocation of proteins from the cytoplasm to the endoplasmic reticulum occurs *via* a conserved Sec61 protein channel. Previously, we reported that mutations in histones cause downregulation of a *CUP1* copper metallothionein, and copper exposure inhibits the activity of Sec61. However, the role of epigenetic dysregulation on the activity of channel is not clear. Identification of cellular factors regulating copper metabolism and Sec61 activity is needed as the dysregulation can cause human diseases. In this study, we elucidate the intricate relationship between copper homeostasis and Sec61-mediated protein translocation. Utilizing copper-sensitive yeast histone mutants exhibiting deficiencies in the expression of *CUP1*, we uncover a copper-specific impairment of the protein translocation process, causing a reduction in the maturation of secretory proteins. Our findings highlight the inhibitory effect of copper on both cotranslational and posttranslational protein translocations. We demonstrate that supplementation with a copper-specific chelator or amino acids such as cysteine, histidine, and reduced glutathione, zinc, and overexpression of *CUP1* restores the translocation process and growth. This study, for the first time provides a functional insight on epigenetic and metabolic regulation of copper homeostasis in governing Sec61-dependent protein translocation process and may be useful to understand human disorders of copper metabolism.

Homeostasis of micronutrients must be tightly regulated in all living organisms because dysregulation can lead to pathological conditions (1). Copper is one of the micronutrient elements required as a cofactor by many of the enzymes that regulate a wide range of physiological processes such as immune response (2), respiration, photosynthesis, DNA damage response (3), lipid metabolism, autophagy (4, 5), and the detoxification of reactive oxygen species (6). Copper homeostasis must be regulated as it can cause cuproptosis by binding to lipoylated enzymes of the tricarboxylic cycle, leading to protein aggregation stress and cell death (7). The optimum cellular copper pool is regulated through many ways, such as import, export, sub-cellular distribution, sequestration by specific metallothionein proteins, utilization as cofactors, and metabolism of copper-binding amino acids such as cysteine (8), histidine (9, 10), methionine, and reduced glutathione (11). Dysregulation in cellular copper metabolism results in disease conditions (12, 13, 14). For example, excess copper in the mouse model of Wilson's disease (Atp7b^−/−^) results in metabolic dysregulations, increasing intermediates of glycolysis and TCA cycle components (15). Copper ions are imported through the cell membrane copper transporters and bind with the chaperone proteins to deliver to target proteins of the cytosol, nucleus, mitochondria, and secretory intracellular compartments (16, 17). For example, under low copper conditions, Mac1 (Metal-binding activator), a transcription factor that acts as a nutritional copper sensor, activates the expression of genes of copper membrane transporters such as *CTR1*, *CTR3*, and *FRE1* (18, 19). On the other hand, under excess copper conditions, Ace1, another transcription factor that acts as a toxic copper sensor, activates the expression of genes involved in copper detoxification, such as *CUP1*, *SOD1*, and *CRS5* (20, 21). Copper can also be toxic because it can compete with other metal ions for binding with the proteins and thiol groups (22, 23). Excessive copper has also been found to impair intracellular protein trafficking in the liver (24). Furthermore, copper accumulation has also been shown to induce endoplasmic reticulum (ER) stress, suggesting ER plays a critical role in Wilson’s disease (25). Copper exists in two oxidation states, Cu+ (cuprous) and Cu^2+^ (cupric). Enzymes primarily utilize Cu^+^ (cuprous) as a cofactor. The Fre1/2 catalyzes the reduction of cupric to cuprous form localized on the membrane of yeast cells. In addition, a novel copper reductase activity (converting cupric to cuprous form) has been identified recently on the surface of the nucleosomal histone octamer complex, suggesting that nucleosome structure can regulate the redox states of copper (26). The structural as well as chemical alterations in the nucleosomes are associated to several diseases in humans. A plethora of mutations have been identified in the histone proteins and chromatin-modifying enzymes which can perturb the structure of chromatin and can cause several pathological conditions, including cancer in humans (27, 28). However, the impact of such mutations on the copper reductase property still needs to be understood entirely. In addition, studies with yeast models have revealed the role of histone proteins, especially the tail regions of H3 and H4, in regulating *CUP1* gene transcription that encodes a major copper metallothionein. Yeast cells with mutations in the N-terminal tail mutants of histones are found to be copper-sensitive due to downregulation of *CUP1*, indicating that mutants probably accumulate more protein-free or labile copper pool, which can nonspecifically bind with proteins (29). However, whether yeast cells with mutations in histones contain more protein-free or labile copper, a condition similar to Wilson's disease, is yet to be identified. In addition, copper exposure has also been shown to inhibit the protein translocation activity of Sec61, a transmembrane channel localized on the ER membrane (30). Sec61 is a conserved heterotrimeric protein complex that conducts cotranslational and posttranslational translocation of secretory pathway proteins (31, 32, 33). Mutations in Sec61 have been shown to correlate with cancerous conditions (34). Inhibitors that can modulate the activity of the Sec61 channel are in high demand to prevent human diseases (35). For example, Coibamide A, a natural product, has been shown to suppress cancerous growth by inhibiting the Sec61 activity (36). Similarly, decatransin, a cyclic decadepsipeptide, has also been shown to inhibit the protein translocation process by binding to Sec61 (37). In addition, pharmacological modulation of the Sec61 channel has been shown to suppress the replication of Dengue and Zika viruses in human and mosquito cells (38, 39).

By keeping the above studies in mind, we hypothesized that yeast cells with mutations in histones that are sensitive to copper and show low expression of *CUP1* probably contain more intracellular total copper or increased labile cellular copper concentration than the WT cells, which might target the Sec61 translocation activity, causing a decrease in the maturation of secretory proteins. To investigate the hypothesis, we utilized copper-sensitive histone H3, H4, and H2A mutants that we identified through screening previously along with WT cells and grew them in the presence of exogenous copper in a time- and dose-dependent manner. Interestingly, as per the hypothesis, we observed a significant decrease in the maturation of secretory proteins in the copper-sensitive histone mutants translocated posttranslationally or cotranslationally. Time-resolved inductively coupled plasma mass spectrometry (ICP-MS) analysis, however, suggests that most of the copper-sensitive histone mutants indeed show intracellular copper concentrations similar to the WT cells. On the other hand, microscopic analysis of cells stained with a fluorescent copper-specific dye suggests that most of the copper-sensitive histone mutants contain slightly increased labile copper pool than the WT cells. By utilizing copper reductase histone mutants, we observed that the reduction in the maturation of secretory proteins could be induced by both cuprous and cupric forms of copper. However, other metals that we tested, including zinc and iron, did not affect the Sec61-mediated protein translocation process. Interestingly, supplementation of a copper chelator, amino acids (cysteine and histidine), glutathione, zinc, and over-expression of *CUP1* restored the growth of copper-sensitive mutant cells and maturation of secretory proteins, indicating the role of metabolic pathways and zinc in the regulation of cellular bioavailable copper concentration. Furthermore, we detected the copper-induced protein translocation defect specific to the fermentation growth phase of yeast cells, suggesting that cellular stress conditions can modulate the copper-mediated inhibition of the protein translocation process.

Altogether, this study, for the first time, demonstrates the metabolic and epigenetic regulation of cellular labile copper levels impacting the Sec61-dependent protein translocation process, which provides a solid basis to explore further the regulatory role of copper in the secretory pathway of proteins.

## Results

### Copper-sensitive histone mutants display protein translocation defects upon copper exposure

**Figure 1:**
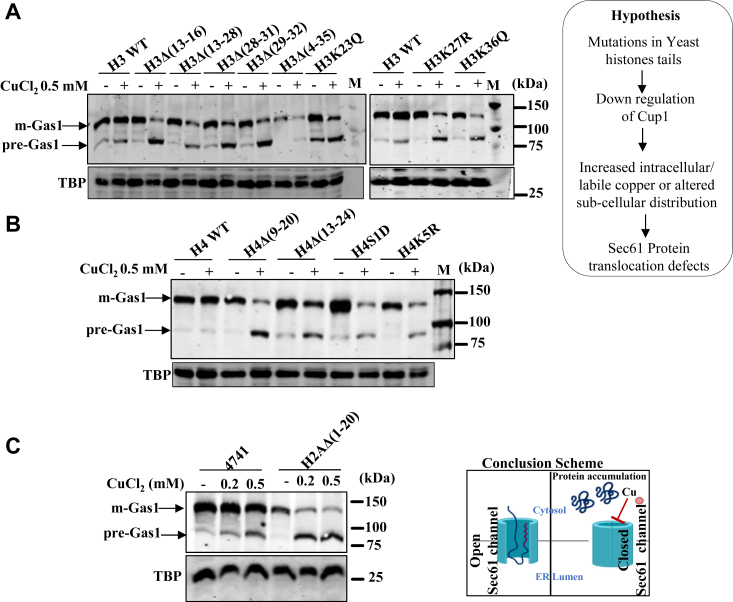
**Copper homeostasis dysregulation leads to the accumulation of immature/premature forms of Gas1, a secretory protein.** A hypothesis, as shown on the *right*, was proposed that copper-sensitive histone mutants may contain intracellular higher copper content/increased labile copper pool/altered subcellular distribution than WT cells which can cause protein translocation defects. *A*, immunoblots of Gas1-GFP–transformed copper-sensitive histone H3 mutants [H3K36Q, H3K23Q, H3K27R, H3Δ(4–35), H3Δ(29–32), H3Δ(13–16), H3Δ(13–28), H3Δ(28–31)] and WT cells treated with 0.5 mM of CuCl_2_.2H_2_O. *B*, immunoblots of Gas1-GFP–transformed copper-sensitive histone H4 mutants [H4Δ(9–20), H4Δ(13–24), H4S1D, H4K5R] and WT cells treated with 0.5 mM of CuCl_2_.2H_2_O. *C*, immunoblots of Gas1-GFP–transformed WT and H2AΔ(1–20) mutant treated with 0.2 mM and 0.5 mM of CuCl_2_.2H_2_O. Gas1-GFP plasmid transformed histone mutants, and WT was grown till an A_600_ ∼1 and then left untreated (−) or treated (+) with indicated concentrations of copper for 2 hours. The total proteins extracted from all these cells were subjected to Western blotting. Western blotting TBP served as a protein loading control. The conclusion scheme shown in the figure below indicates that the copper inhibits the Sec61 protein translocation process. Gas1, glycophospholipid-anchored surface protein; TBP, TATA-binding protein.

**Figure 2:**
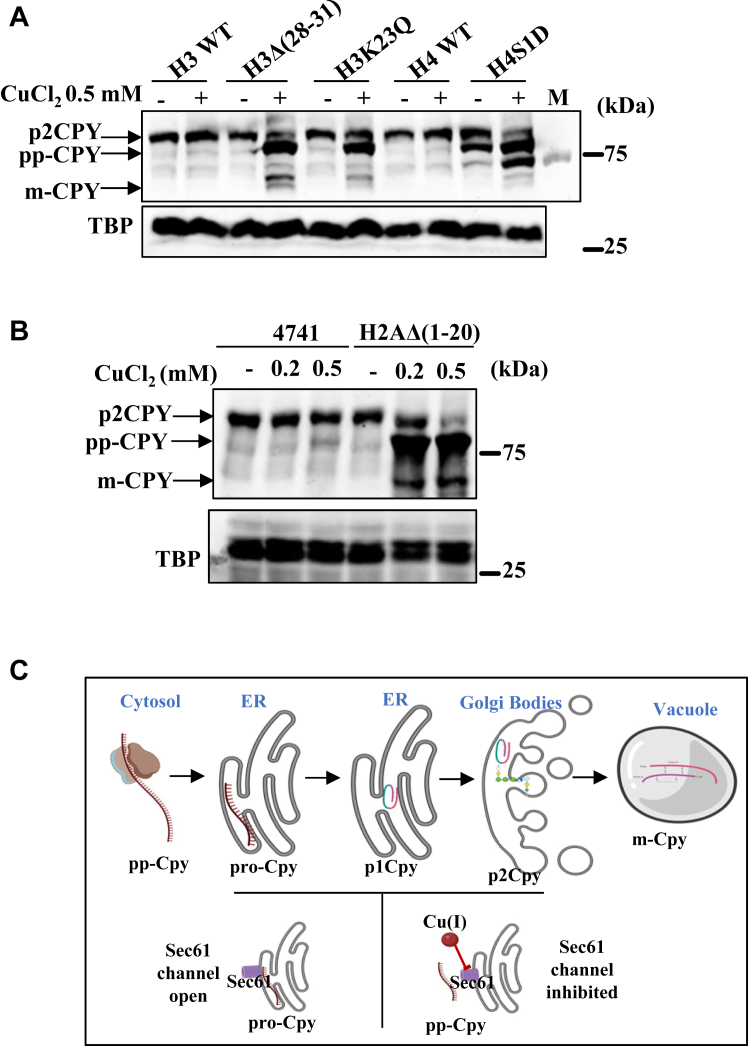
**Copper homeostasis dysregulation leads to the accumulation of precursor CPY, a secretory protein.***A*, immunoblots of CPY myc-tagged protein in copper-sensitive histone H3 and H4 mutants [H3Δ(28–31), H3K23Q, and H4S1D] and WT cells treated with 0.5 mM of CuCl_2_.2H_2_O. *B*, immunoblots of CPY myc-tagged in WT and H2A mutant H2AΔ(1–20), mutant treated with 0.2 mM, and 0.5 mM of CuCl_2_.2H_2_O. CPY myc-tagged histone mutants andWT cells as indicated were grown to an A_600_ ∼1 and then left untreated (−) or treated (+) with indicated concentrations of copper for 2 h. The total proteins extracted from these cells were subjected to Western blotting. TBP Western blot served as a protein loading control. *C*, schematic representation of different subcellular forms of CPY. CPY, Carboxypeptidase Y; TBP, TATA-binding protein.

Mutations in histones are associated with several human diseases. However, the impact of histone mutations on micronutrients' cellular homeostasis needs to be clearly understood. Previously, by screening yeast histone mutants with metal ions, we identified the regulatory role of core histone tails in copper homeostasis. Our studies suggested that the N-terminal tails of histone H3, H4, and H2A are essential for the expression of *CUP1*, a major copper-specific metallothionein. We observed severe downregulation of the *CUP1* gene in the yeast cells with point and truncation mutations in the tail regions of H3 [H3K36Q, H3K23Q, H3K27R, H3Δ(4–35), H3Δ(29–32), H3Δ(13–16), H3Δ(13–28), H3Δ(28–31)], H4 [H4Δ(9–20), H4Δ(13–24), H4S1D, H4K5R], and H2A [H2AΔ(1–20)] and growth retardation upon copper treatment. In another study, we showed that copper can inhibit the activity of a conserved Sec61 protein translocation channel located on the ER membrane, leading to cytosolic accumulation of premature forms of secretory pathway proteins. Therefore, we anticipated that copper-sensitive histone mutant cells might accumulate more intracellular copper or more labile copper ions, which can target the activity of Sec61 and slow down the translocation process of secretory proteins, as shown in the scheme next to Figure 1*A*. We studied the secretory pathway by employing well-established examples of secretory proteins, such as Gas1 (Beta-1,3-glucanosyltransferase) and carboxypeptidase Y (CPY). Yeast histone mutant cells and respective WTs expressing GFP-tagged Gas1 or myc-tagged CPY were treated with 0.2 and 0.5 mM of CuCl_2_ for 2 h. Cells were harvested, and whole cell extracts were made to test the mature and premature forms of Gas1 and CPY proteins of the secretory pathway through Western blotting experiments. The growth phenotype was not affected much at these concentrations of CuCl_2_ of both the WT and copper-sensitive mutants examined by spot assays (Fig. S1, *A* and *B*) and CFU (Fig. S1, *C*–*F*). Interestingly, we detected a significant increase in the premature form of Gas1 in histone H3 mutants (Fig. 1*A*), H4 mutants (Fig. 1*B*), H2A mutants (Fig. 1*C*), and premature forms of CPY in H3, H4, and H2A mutants (Fig. 2, *A* and *B*). In most mutants, the percentage ratio between mature and immature (also known as premature) forms of Gas1 is about 1:1 upon copper treatments for 2 h compared to WT cells. The immature form of Gas1 protein in the WT cells is found in the range of 10 to 20%; however, in mutants, it is about 50% or even more upon copper treatments. In fact, in some mutants such as H3Δ(13–16), H4Δ(9–20), H4K5R, and H2AΔ(1–20), the percentage of immature Gas1 protein is increased up to 70% (only 30% mature form) upon 0.5 mM of copper treatments (Figs. S4, *A* and S5, *A* and *B*). We also examined the effect on protein translocation process at a very low concentration of CuCl_2_ in the range of 10 to 200 μM with WT cells and found that even low concentration can induce the defect (Fig. S3, *H*–*L*). The CPY can exist in multiple subcellular forms, starting from cytosol to ER, golgi, and vacuoles (Fig. 2*C*), but the intensities of different forms vary in the mutants. For example, we could detect apparent differences in the intensities of CPY protein bands in H3Δ(28–31), H3K23Q, and H4S1D mutants, upon copper treatment. We could detect about 70% premature cytosolic form (pre-pro-CPY or pp-CPY) of the CPY protein band in copper-sensitive histone mutants [H3Δ(28–31), H3K23Q, H4S1D, and H2AΔ(1–20)] than WT cells upon 0.5 mM of copper treatments (Fig. S6, *A* and *B*). In addition, we could detect multiple forms of CPY even without copper treatment in the H4S1D mutant. These observations are consistent and highly reproducible, as evident from several biological replicates (Figs. S2, *A*–*N* and S3, *A*–*F*). All these Western blotting experiments with additional copper-sensitive histone mutants were performed just to support the original hypothesis and conclusions. Although there was a significant increase in premature forms of Gas1 and CPY proteins in almost all copper-sensitive H3, H4, and H2A mutants compared to the WT cells, it was not at the same level. The possible explanation for the variation in the levels of premature protein bands of Gas1 and CPY among histone mutants could be attributed to differences in the rate of protein translocation, as the cellular labile copper pool and copper reductase activities may not be at the same level across all mutants. Further exploration is needed in this regard. Another reason could be the differential expression of Sec61 channel protein among mutants. However, we did not find any noticeable differences in the expression of Sec61 across mutants; it is same as the WT cells (Fig. S3*G*).

To further understand whether the impact on the protein translocation process is specific to copper ions, WT cells were treated with the salt forms of several other transition metal ions (Cu, Fe, Zn) and toxic metals (Ni, Cd, and As) at the concentrations that did not affect the growth of cells significantly (Fig. S7, *A* and *B*). Western blotting was then performed to analyze the forms of the Gas1 secretory protein. To our surprise, we observed an increase in the premature form of Gas1 only in the case of copper treatment (Fig. S7, *C*–*E*), not in cells treated with other metal ions. Together, these data provide suggest that epigenetic dysregulation of copper homeostasis can increase the available cellular labile copper pool, leading to cytosolic accumulation of secretory pathway proteins by inhibiting the activity of the Sec61 channel.

### Copper-sensitive yeast histone mutant cells contain intracellular total copper content similar to WT cells but contain increased labile copper pool

As yeast cells with mutations in the tail regions of nucleosomal histone proteins, H3, H4, and H2A, are copper-sensitive and display copper-dependent protein translocation defect, we hypothesized that mutants might contain more intracellular total copper content or more labile copper pool. Moreover, we previously reported downregulation of *CUP1* in copper-sensitive histone mutants which indicates that mutants may contain more labile pool of copper. We also measured the *CUP1* expression in additional copper-sensitive histone mutant strains and found that *CUP1* is downregulated as compared to WT cells (Fig. S1*G*). Therefore, to test the hypothesis, we first measured the intracellular total copper content in a few of the histone H3 mutants and the WT cells by employing ICP-MS. All the histone H3 mutant yeast strains [H3Δ(4–35), H3Δ(13–16), H3Δ(28–31), H3K36Q, H3K27R, H3K23Q, H3Δ(29–32), and H3Δ(13–28)] and WT were grown in 10 ml of SC medium until 0.8 to 1.0 A_600_ and were harvested by low-speed centrifugation. Cells were subsequently washed two times with milliQ distilled water, resuspended in 5.0 ml of sterile milliQ distilled water, boiled for 15 min, centrifuged, and supernatants (metal extracts) were collected into new tubes. Appropriate dilutions of collected supernatants of each yeast strain were made and subjected to ICP-MS analysis to measure the intracellular copper contents. Before the ICP-MS analysis of yeast samples, the machine was calibrated with internal standard samples and increasing concentrations of copper ranging from 0.1 to 150 ppb. Careful analysis of ICP-MS readings obtained with supernatants of yeast strains suggests that mutant cells contain intracellular copper content similar to that of WT cells (Fig. S7*N*). Although the intracellular copper content in most of the copper-sensitive histone mutants is similar to the WT cells, however, two of the mutants [H3Δ(4–35) and H3K36Q] showed more intracellular copper content than the WT cells. It is quite possible that these two mutants may have imported more copper than other mutants and the WT cells as an adaptive mechanism to restore Cu+ levels to survive under unknown stress conditions induced by these mutations.

**Figure 3:**
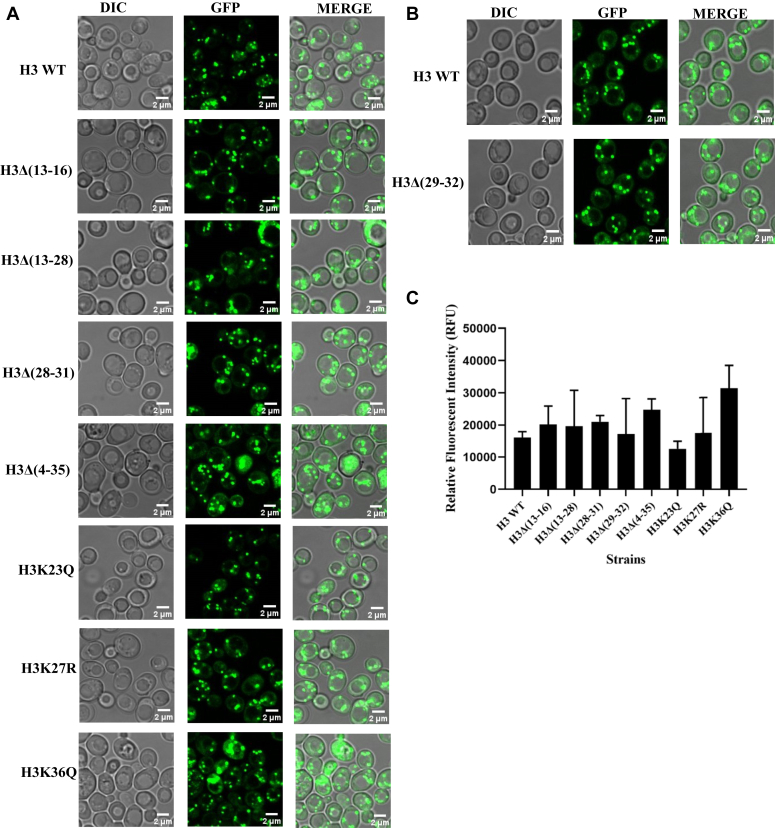
**Measurement of labile copper pool in WT yeast and histone H3 mutant cells.***A* and *B*, confocal microscopy images of yeast cells; WT and mutants of histone H3. Equal OD of exponentially growing cells were stained with CS1 (copper sensor-1) probe purchased from MedChemExpress. The culture of cells grown till the mid-log phase were harvested and washed twice with 1X PBS. The cells were subsequently resuspended in 1 ml of PBS and treated with 2.4 μM of copper sensor probe, CS1, for 10 min in dark condition at 30 °C and resuspended in ∼25 μl of same, and images were taken with Olympus FV-3000 confocal microscope. The ex/em used for CS1 is 473−491/513−533 nm, visualized by using GFP channel and DIC. For control, unstained cells were used. *C*, data indicates the relative fluorescent intensity units (RFU) quantified by using ImageJ software. Data represents the means and SD from two independent biological repeats (n = 2).

To gain more insight, we also measured the cellular labile copper pool by using a fluorescent copper-specific probe (copper sensor-1). The analysis of microscopic fluorescent images of cells after treatment with a copper-specific probe indicates that most of the mutants contain 10 to 20% more labile copper pool than the WT cells (Figs. 3, *A*–*C* and S7, *J*–*M*). Thus, it is possible that the labile copper which is slightly more in the copper-sensitive mutants than the WT cells can cause slow growth phenotype by inhibiting the translocation process of proteins. Since the expression of *CUP1* (a major high affinity copper metallothionein) is downregulated in most of the copper-sensitive histone mutants than the WT cells, it is quite possible that in the mutants, cellular labile copper is mobilized towards other low affinity proteins including Sec61 to which copper can bind and inhibit their function. It is also possible that labile copper concentration in the mutants may not remain labile although CUP1 is downregulated, it may weakly associate with other proteins such as Sec61.

### Copper inhibits both cotranslational and posttranslational translocation processes

**Figure 4:**
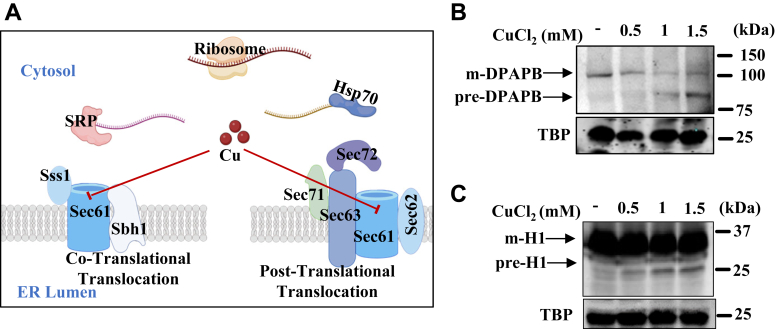
**Copper can also inhibit the cotranslational protein translocation process.***A*, schematic representation showing copper-mediated inhibition of protein translocation by binding to Sec61. Cotranslational translocation of secretory pathway proteins translocates inside the ER lumen through the Sec61 trimeric complex comprising Sec61, Sss1, and Sbh1, while the posttranslational translocation of the secretory pathway protein substrate takes place through the Sec61 trimeric complex along with other subunits, that is, Sec62, Sec63, Sec71, and Sec72. *B* and *C*, immunoblots of DPAPB-HA and H1-HA upon CuCl_2_.2H_2_O (0.5 mM, 1 mM, 1.5 mM) treatment of WT cells. The respective plasmid-transformed WT yeast cells were grown to an A_600_ ∼ 1 and then left untreated (−) or treated (+) with indicated concentrations of copper for 2 hours. The total protein samples extracted from these treated cells were subjected to Western blotting of HA-tagged DPAPB and H1 by using α-HA antibody. TBP expression of each blot served as a protein loading control. Molecular weight markers are indicated on right side of the blots by aligning marker lane. DPAPB, dipeptidyl aminopeptidase B; H1, human asialoglycoprotein receptor H1; TBP, TATA-binding protein.

Protein translocation into the ER in eukaryotes can occur either cotranslationally, where insertion into the ER lumen or membrane happens simultaneously with protein synthesis, or post-translationally, where translocation occurs after the polypeptide has been fully synthesized (Fig. 4*A*). Using examples of Gas1 and CPY secretory proteins, we have previously shown that copper can inhibit posttranslational translocation activity of the Sec61 channel. To further understand whether copper can also inhibit the cotranslational translocation process, we examined the translocation of DPAPB (dipeptidyl aminopeptidase B), H1 (a membrane protein), and Suc2 (invertase, sucrose hydrolyzing enzyme) proteins which are known to enter ER through the cotranslational translocation mode. WT cells were transformed with plasmids to express HA-tagged proteins: DPAPB, H1, and Suc2. Transformed cells at 0.8 to 1.0 A_600_ were treated for 2 h with increasing concentrations of copper chloride as indicated, in case of DPAPB and H1 and 4 h treatment in case of Suc2, as 2 h treatment did not show any noticeable effect on the translocation of Suc2 protein. The cells were subsequently harvested and whole cell extracts were made as described in Experimental procedures, and Western blotting was performed. We anticipated that copper may inhibit both modes of the translocation process as previous studies have shown that copper can inhibit the translocation activity of the Sec61 channel. Indeed, we detected an increase in the premature forms of DPAPB, H1, and Suc2 proteins (Figs. 4, *B* and *C* and S7*H*). There is an increase of 60% in the premature form of DPAPB (pre-DPAPB) and a reduction of 50% of the mature form upon 1 mM and 1.5 mM of copper chloride treatment (Fig. S7*F*). Similarly, we detected an increase of 25% in the premature form of H1 (pre-H1) upon treatment with 1 mM and 1.5 mM of copper chloride (Fig. S7*G*). On the other hand, treatment of cells with copper showed an increase in precursor Suc2 by 40% (Fig. S7*I*) in 4 h. This observation suggests that copper can inhibit protein translocation of both the modes, cotranslational and posttranslational.

### A copper-specific chemical chelator suppresses copper-induced protein translocation defects in the histone tail mutants

**Figure 5:**
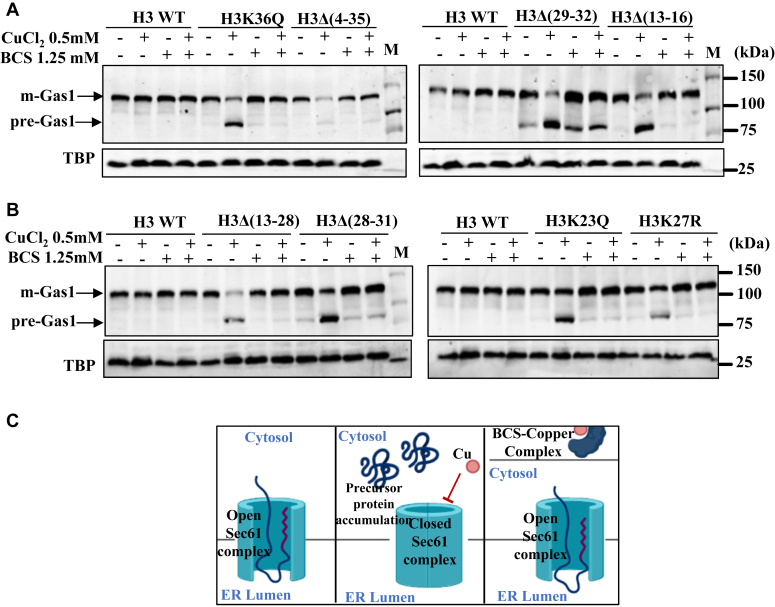
**BCS supplementation suppresses the copper mediated protein translocation defect.***A* and *B*, immunoblot of Gas1-GFP in copper-sensitive histone H3 mutants [H3K36Q, H3K23Q, H3K27R, H3Δ(4–35), H3Δ(29–32), H3Δ(13–16), H3Δ(13–28), H3Δ(28–31)] and WT cell treated with CuCl_2_.2H_2_O (0.5 mM), BCS (1.25 mM), and co-treatments. Gas1-GFP plasmid-transformed histone mutants and WT was grown to an A_600_ ∼ 1 and then left untreated (−) or treated (+) with indicated concentrations of copper and BCS for 2 hours. TBP Western blotting served as a protein loading control. *C*, schematic representation of copper-mediated protein translocation defect and rescue by co-treatment with copper chelator BCS. Gas1, glycophospholipid-anchored surface protein; TBP, TATA-binding protein.

To further dissect that the inhibition of the Sec61-dependent protein translocation process is copper-specific in the histone mutants, we tested the growth of WT and the copper-sensitive core histone mutants, H3 and H4 in the presence of copper chloride, bathocuproine sulphonate (BCS) and co-treatments, a copper specific chemical chelator (Fig. 5*C*). As we expected, BCS supplementation completely rescued the growth of mutant cells (Fig. S8, *A* and *B*).

Next, we performed the Western blotting experiments to inspect the influence of BCS on the copper-dependent inhibition of the protein translocation process by taking the examples of Gas1 and CPY proteins. Respective WT and copper-sensitive histone mutant cells of H3[H3Δ(4–35), H3Δ(13–16), H3Δ(28–31), H3K36Q, H3K27R, H3K23Q, H3Δ(29–32), H3Δ(13–28)] and H4[H4Δ(9–20), H4Δ(13–24), H4K5R, and H4S1D] were treated with 0.5 mM of copper chloride or copper chloride + BCS for 2 h as described in Experimental procedures. Subsequently, cells were harvested, and whole cell extracts were made. Extracts were resolved on SDS-PAGE, and Western blotting was performed to measure the mature and premature forms of Gas1 protein. As expected, compared to WT cells, we could see a significant increase in premature Gas1 (pre-Gas1) protein band in all the mutants of H3 and H4 upon copper chloride treatment. However, in cotreatments (copper chloride + BCS), the appearance of the premature form of the Gas1 protein band was supressed entirely (Figs. 5, *A* and *B* and S9, *A*–*E*) in all the mutants, suggesting that inhibition of the Sec61 protein translocation process is highly copper-specific. In almost all the mutants of histone H3 and H4, we observed 50 to 70% of the premature form of Gas1 (pre-Gas1) and 30 to 50% mature form upon 0.5 mM of copper chloride treatment, while in WT cells, the premature form is only 10 to 20%. However, cotreatment with BCS increased the mature form of Gas1 protein up to 80 to 90%, the same as WT untreated cells (Fig. S10, *A* and *B*).

### Copper-dependent inhibition of yeast cell growth and Sec61-mediated translocation process can be restored by the supplementation of cysteine, histidine, and glutathione

**Figure 6:**
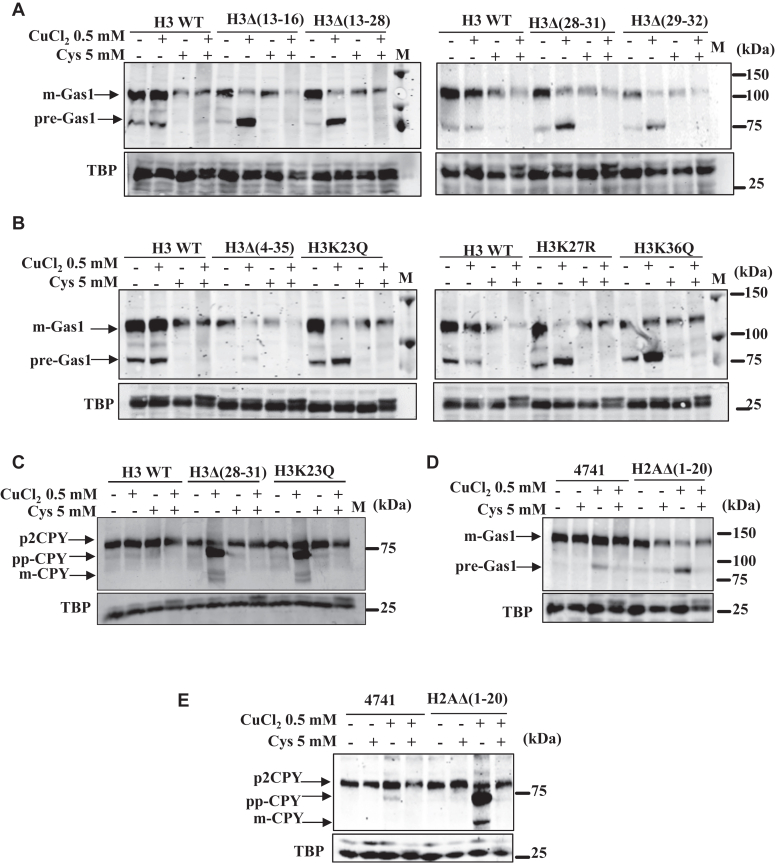
**Cysteine supplementation prevents copper-induced defects in the protein translocation process.***A* and *B*, immunoblots of Gas1-GFP–transformed copper-sensitive histone H3 mutants and WT yeast cells treated with CuCl_2_.2H_2_O (0.5 mM), cysteine (5 mM), and co-treatments. *C*, immunoblots of CPY myc-tagged copper-sensitive histone H3 mutants and WT cells treated with CuCl_2_.2H_2_O (0.5 mM), cysteine (5 mM), and co-treatments. *D*, immunoblot of Gas1 GFP-transformed WT and H2A mutant, H2AΔ(1–20)] treated with CuCl_2_.2H_2_O (0.5 mM), cysteine (5 mM), and co-treatments. *E*, immunoblot of CPY myc-tagged WT cells and H2A mutant, H2AΔ(1–20) treated with CuCl_2_.2H_2_O (0.5 mM), cysteine (5 mM), and co-treatments. TBP Western blot served as a protein loading control. The untreated (−) or treated (+) with indicated concentrations of copper and cysteine. CPY, Carboxypeptidase Y; Cys, Cysteine; Gas1, glycophospholipid-surface protein; M, protein ladder; TBP, TATA-binding protein.

**Figure 7:**
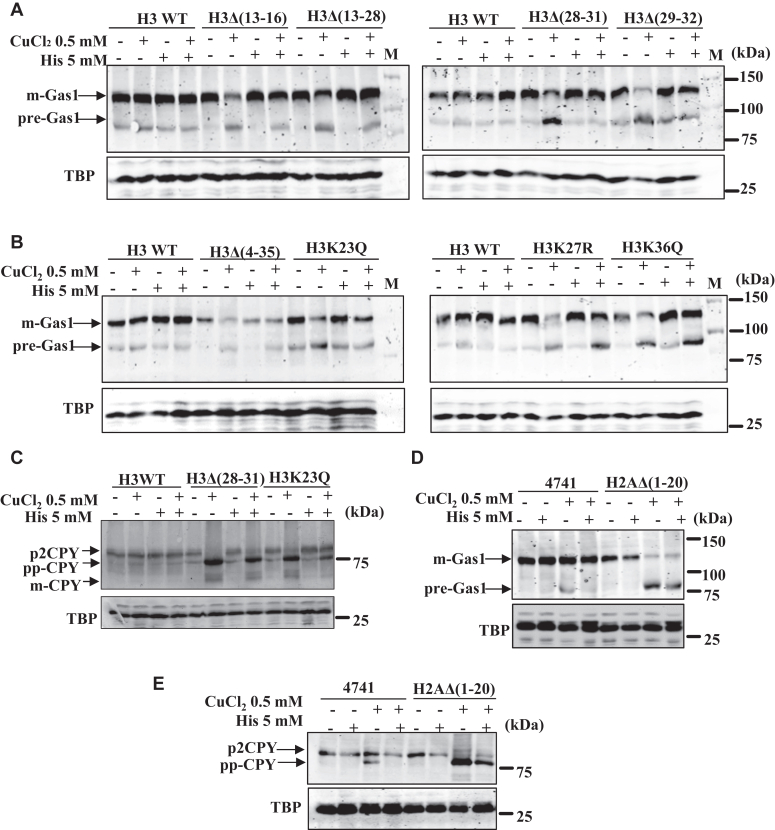
**Histidine supplementation halts copper-mediated protein translocation defects.***A* and *B*, immunoblots of Gas1-GFP–transformed copper-sensitive histone H3 mutants as indicated and WT yeast cells treated with CuCl_2_.2H_2_O (0.5 mM), histidine (5 mM), and co-treatments. *C*, immunoblots of CPY-myc–tagged histone H3 mutants and WT cells treated with CuCl_2_.2H_2_O (0.5 mM), histidine (5 mM), and co-treatments. *D*, immunoblot of Gas1-GFP–transformed histone H2A mutants [H2AΔ(1–20)] and WT yeast cells treated with CuCl_2_.2H_2_O (0.5 mM), histidine (5 mM), and co-treatments. *E*, immunoblot of CPY myc-tagged histone H2A mutant as indicated and WT cells treated with CuCl_2_.2H_2_O (0.5 mM), histidine (5 mM), and co-treatments. TBP Western blot served as a protein loading control. The untreated (−) or treated (+) with indicated concentrations of copper and histidine. CPY, Carboxypeptidase Y; His, Histidine; Gas1, glycophospholipid-anchored surface protein; M, protein ladder; TBP, TATA-binding protein.

**Figure 8:**
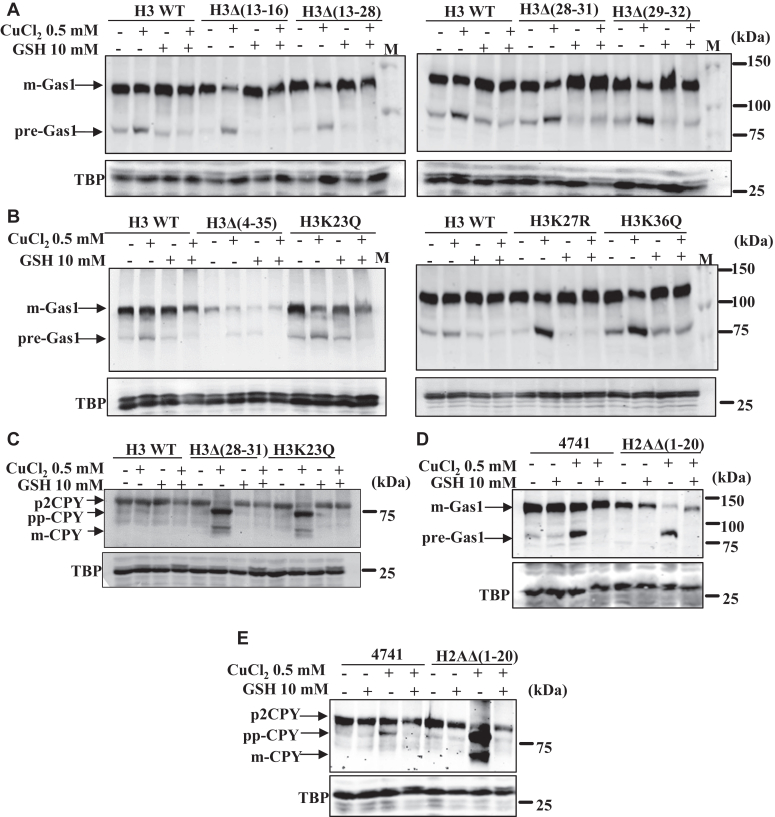
**The addition of reduced glutathione prevents copper-induced protein translocation defects.***A* and *B*, immunoblots of Gas1-GFP–transformed copper-sensitive histone H3 mutants and WT cells treated with CuCl_2_.2H_2_O (0.5 mM), glutathione (10 mM), and co-treatments. *C*, immunoblots of CPY myc-tagged histone H3 mutants and WT cells treated with CuCl_2_.2H_2_O (0.5 mM), glutathione (10 mM), and co-treatments. *D*, immunoblots of Gas1-GFP–transformed histone H2A mutant [H2AΔ(1–20)] and WT cells treated with CuCl_2_.2H_2_O (0.5 mM), glutathione (10 mM), and co-treatments. *E*, immunoblots of CPY myc-tagged WT and histone H2A mutant yeast cells [H2AΔ(1–20)] treated with CuCl_2_.2H_2_O (0.5 mM), glutathione (10 mM), and co-treatments. TBP Western blot served as a protein loading control. The untreated (−) or treated (+) with indicated concentrations of copper and glutathione. CPY, Carboxypeptidase Y; Gas1, glycophospholipid-anchored surface protein; GSH, Glutathione; M, protein ladder; TBP, TATA-binding protein.

As copper is essential and can be toxic, its uptake, export, transport, and utilization must be tightly regulated, and dysregulation in cellular copper homeostasis can cause diseases in humans (12). The copper in eukaryotes can exist in strongly protein-bound, weakly protein bound, and labile forms. In addition to acting as a cofactor for many enzymes, copper coordinates with methionine, cysteine, histidine, and glutathione. Furthermore, metabolic profiling studies with Wilson’s disease patients have identified the dysregulation in several metabolites, including a significant decrease in cysteine and glutathione levels (40). Therefore, it can be concluded that copper homeostasis is not only regulated by the cellular concentrations of transporters (export/import), cellular chaperones, and reductases but also through the metabolism of amino acids; methionine, cysteine, histidine, and glutathione. The amino acid homeostasis is regulated in several steps; import, synthesis, utilization, recycling, and catabolism (41). Amino acids are recycled inside the cellular environment by the process of autophagy. In addition, vacuolar pH regulation allows amino acid sequestration and utilization from the organelle, which is essential for cellular functions such as mitochondrial and ribosomal and regulates cell size (42). To this end, we performed many experiments to identify the role of these molecules (methionine, cysteine, histidine, and glutathione) on the copper-dependent inhibition of the Sec61 protein translocation process. First, we tested the growth of respective WT cells and copper-sensitive yeast mutants of histone H3[H3Δ(4–35), H3Δ(13–16), H3Δ(28–31), H3K36Q, H3K27R, H3K23Q, H3Δ(29–32), H3Δ(13–28)] and H4[H4Δ(9–20), H4Δ(13–24), H4K5R, and H4S1D] and histone H2A [H2AΔ(1–20), H2AΔ(1–20)H3R2A, H2AΔ(1–20)H3K4A] at different concentrations of copper chloride and copper chloride + Cys/His/Met/Glutathione as described in Experimental procedures. Among histone H2A mutants that we took for the study [H2AΔ(1–20), H2AΔ(1–20)H3R2A, H2AΔ(1–20)H3K4A], only the H2AΔ(1–20) is found to be copper-sensitive. As copper coordinates with Met, His, Cys, and glutathione, adding these can reduce the cellular bioavailability of copper. Indeed, the slow growth phenotype of the mutants in the presence of copper chloride was suppressed (growth rescued) by the supplementation of His and Cys and reduced glutathione (Figs. S11, *A*–*D* and S12, *A*–*F*). We then performed the Western blotting experiments to examine the influence of Cys/His/glutathione on the copper-dependent protein translocation defects by taking examples of Gas1 and CPY proteins. Histone mutants and WT cells were treated with copper chloride or copper chloride + Cys/His/glutathione for 2 h. Subsequently, cells were harvested, and whole cell extracts were made. Extracts were resolved on SDS-PAGE, and Western blotting was performed to test the percentage of mature and premature forms of Gas1 and CPY protein bands. As expected, compared to WT cells, we observed a significant increase in the premature forms of Gas1 (pre-Gas1) and CPY (pp-CPY) protein bands in all H3, H4, and H2A mutants upon copper chloride treatments. However, in cotreatments (copper chloride + Cys/His/Glutathione), the appearance of premature forms of Gas1 and CPY protein bands were eliminated (Figs. 6, *A*–*E*, 7, *A*–*E*, 8, *A*–*E*, and S13, *A*–*F* and S14, *A*–*G*) in all the copper-sensitive histone mutants suggesting that copper-induced defects in Sec61 protein translocation process can be suppressed by the addition of Cys/His/glutathione. As copper is known to bind more strongly with cysteine and glutathione than histidine and methionine, we observed a significant increase in the mature forms of Gas1 (m-Gas1) and CPY (p2CPY or mCPY) upon cotreatment with cysteine or glutathione. The cytosolic forms of Gas1 and CPY were observed to be about 50% or even more in some mutants upon 0.5 mM of copper treatment. However, supplementation with 5 mM of cysteine or 10 mM glutathione increased the mature forms to 90%, almost the same as copper untreated cells (Figs. S15, *A* and *B*, S16, *A*–*C*, S17, *A*–*C*, S18, *A* and *B*, S19, *A*–*C* and S20, *A* and *B*). Further we wanted to test whether copper can coordinate with cysteine/histidine/glutathione outside cells, inside the cells, or both ways. To test these possibilities, we first grew the cells (WT and only one of the histone H3 mutants, H3Δ(28–31) in growth medium containing a ligand (GSH/cysteine/histidine) for couple of hours and then medium was replaced with the fresh medium but without these molecules (GSH/cysteine/histidine). The cells were subsequently treated with 0.5 mM of CuCl_2_ for 2 h. We observed a very good rescue of protein translocation defect almost same as cotreated (CuCl_2_ + GSH/cysteine/histidine) conditions, although some amount of immature Gas1 protein was detected suggesting that a fraction of these molecules can also coordinate with the copper outside the cells as well (Figs. S13, *G* and *H* and S14*K*).

The above results suggest that the copper chloride–induced protein translocation defect can be suppressed by the binding of copper to these ligand molecules outside as well as inside the cells. Altogether based on the above investigations, we can conclude that copper-induced protein translocation defects or rate of protein translocation through Sec61 can be controlled through genetic or chemical modulation of pathways regulating the cellular levels of Cys/His/glutathione.

### Zinc supplementation suppresses the copper-induced protein translocation defect

**Figure 9:**
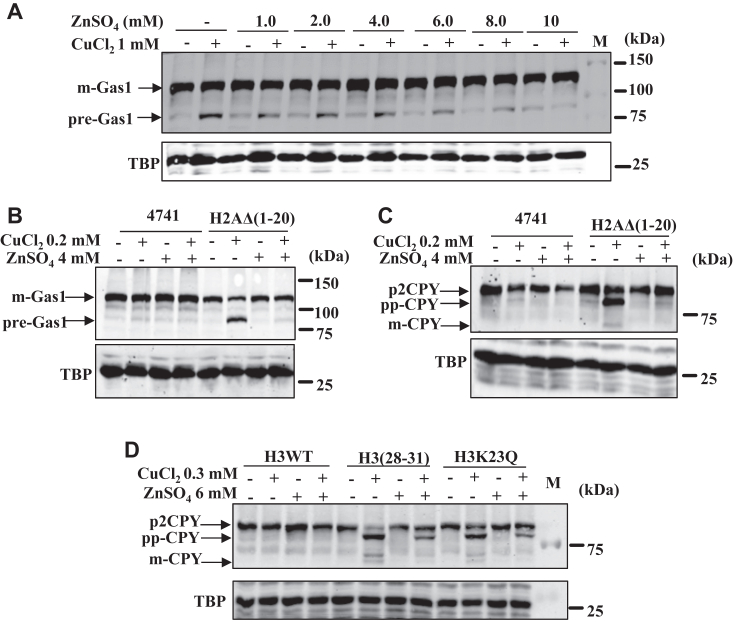
**Zinc supplementation suppresses copper-mediated inhibition of protein translocation process.***A*, immunoblot of Gas1-GFP–transformed WT yeast cells treated with CuCl_2_.2H_2_O (1 mM) and cotreated with different concentrations of ZnSO_4_.7H_2_O (1.0 mM, 2.0 mM, 4.0 mM, 6.0 mM, 8.0 mM, and 10 mM). *B*, immunoblot of Gas1-GFP transformed in WT and histone H2AΔ(1–20) mutant treated with CuCl_2_.2H_2_O (0.2 mM), ZnSO_4_.7H_2_O (4 mM), and co-treatments. *C*, immunoblot of CPY myc-tagged in WT and histone H2AΔ(1–20) mutant, treated with CuCl_2_.2H_2_O (0.2 mM), ZnSO_4_.7H_2_O (4 mM), and co-treatments. *D*, immunoblots of CPY myc-tagged copper-sensitive histone H3 mutants and WT cells treated with CuCl_2_.2H_2_O (0.3 mM), ZnSO_4_.7H_2_O (6 mM), and co-treatments. TBP Western blot served as a protein loading control. The untreated (−) or treated (+) with concentrations of copper and zinc. CPY, carboxypeptidase Y; Gas1, glycophospholipid-anchored surface protein; GSH, glutathione reduced; M, protein ladder; TBP, TATA-binding protein.

The intracellular concentrations of transition metal ions must be regulated as they are essential components of various biological processes. The abnormal cellular levels or subcellular distributions of metals and crosstalk among multiple metals can cause pathological conditions, including neurodegenerative, immunological, cancer, and other genetic disorders. For example, low or higher cellular levels of copper are associated with genetic disorders. Studies suggest that excessive zinc intake competes with copper for absorption in the gut, indicating that cellular ratios among metals are critical for regulating life processes. For example, the FDA approved the use of zinc in treating Wilson's disease. Zinc is thought to upregulate the intestinal cell metallothionein and inhibit copper absorption (43). Another study reveals that zinc is required for the copper reductase activity of the nucleosomal histone octamer. This information inspired us to investigate the role of zinc in the copper-dependent inhibition of the Sec61 protein translocation process. To this end, WT and the copper-sensitive histone mutant cells were grown for overnight culture. Cells were diluted to 1 A_600_ in distilled water serially as mentioned in Experimental procedures and spotted on SC agar solid medium in petridishes consisting of copper, zinc, and copper + zinc and growth was recorded after 72 h (Fig. S21, *A*–*C*). The amount of zinc (4–6 mM) that can suppress the toxic effects of copper (0.2–0.3 mM) did not affect the growth of cells tested by growth curve analysis (Fig. S21, *D*–*I*). Careful analysis suggests that the growth defects induced by the copper can be suppressed by the supplementation of zinc (Fig. S21). This observation suggests that copper-induced inhibition of the protein translocation process can be restored by adding zinc. To test it, overnight cultures of WT and the copper-sensitive histone mutant cells were seeded at 0.2 A_600_ and grown till 0.8 to 1.0 A_600_. Subsequently treated with copper, zinc, and copper + zinc for 2 h, harvested, whole cell extracts were prepared, and Western blotting experiments were performed to examine the mature and premature forms of Gas1 and CPY protein bands. Observation suggests that the copper-induced appearance of premature forms (cytosolic forms) of both Gas1 and CPY decreases in cotreatments (Cu + Zn) in WT and the mutants (Fig. 9, *A*–*D* and S22, *A*–*F*). Identifying the impact of Zn on the copper-mediated effects on the levels of mature or immature forms of Gas1 or CPY was a very challenging task because multiple cellular factors can affect the ratio between copper and zinc. After lots of optimization, we observed that 0.2 mM or 0.3 mM of copper chloride–induced effect on the levels of mature or premature forms of Gas1 or CPY can be reversed by supplementation of 4 to 6 mM of zinc, although it varies among mutants (Figs. S23, *A*–*C* and S24*A*). The above observations suggest that the cellular concentrations of copper and zinc may play a critical role in regulating the rate of the protein translocation process and mitigating toxic effects on cell growth.

### Overexpression of *CUP1* prevents the copper-induced protein translocation defect

The *CUP1* gene in *Saccharomyces cerevisiae* is essential in preventing copper-mediated cell death. However, *CUP1* is not required for cell growth without exogenous copper. As we observe the inhibition of Sec61 channel activity in the presence of exogenous copper, overexpression of *CUP1* should restore the channel's activity. The WT and the histone mutants were transformed with an empty vector and *CUP1* overexpression vector to examine it. The growth of transformed cells was tested in the presence of exogenous copper. Finding a concentration of exogenous copper that does not inhibit the growth of *CUP1* overexpressing cells was challenging. In another words, it was very challenging to find the concentration of exogenous CuCl_2_ whose effect on protein translocation process can be tolerated by the “*CUP1* overexpressing cells.” The idea here was to test the effect of *CUP1* expression on copper-induced protein translocation defect. To test it, the “*CUP1* overexpressing cells” were grown in the presence of increasing concentration of CuCl_2_. The careful analysis of the results suggests that growth retardation defect at 0.75 mM and 1.0 mM of exogenous copper chloride can be suppressed by the overexpression of *CUP1* (Fig. S25*A*). The expression of the *CUP1* protein was examined by Western blotting in the WT and the mutant cells, and it was found to be very similar in all the strains (Fig. S25, *B*, *C*, *E* and *F*).

**Figure 10:**
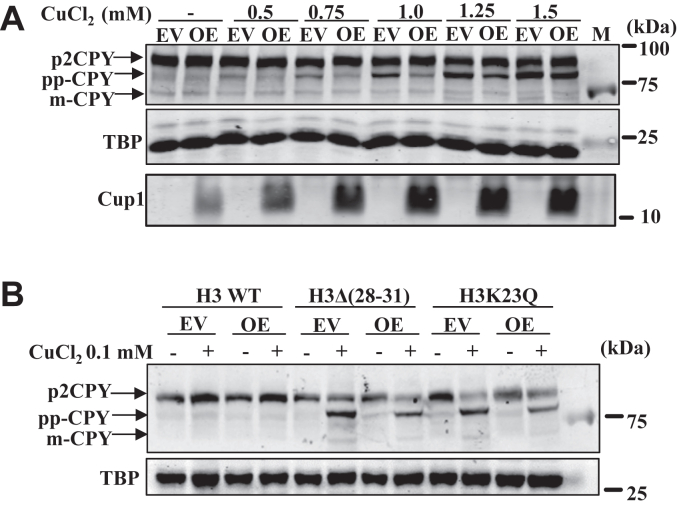
***CUP1* overexpression suppresses copper-induced protein translocation defects.***A*, immunoblot of *CUP1* overexpression (OE) or empty vector (EV) transformed CPY myc-tagged WT cells treated with increasing concentrations of CuCl_2_.2H_2_O (0.5 mM, 0.75 mM, 1.0 mM, 1.25 mM, 1.5 mM). *B*, immunoblot of *CUP1* overexpression or empty vector transformed CPY myc-tagged histone H3 mutants as indicated and WT cells treated with 0.1 mM CuCl_2_.2H_2_O. TBP Western blot served as a protein loading control. The untreated (−) or treated (+) with indicated concentrations of copper chloride. CPY, carboxypeptidase Y; TBP, TATA-binding protein.

Further, to investigate the impact of *CUP1* overexpression on the copper-mediated inhibition of the Sec61 protein translocation channel, the *CUP1* transformed CPY-myc tagged WT cells, and a few copper-sensitive histone mutants were treated with different concentrations of copper chloride for 2 hours. Interestingly, in the *CUP1*-transformed WT cells, the intensity of the premature form of the CPY protein band (pp-CPY) upon copper treatment (0.75 and 1.0 mM) was decreased significantly in comparison to the cells transformed with empty vector (Figs. 10*A*, S25, *B* and *C*, and S26*A*). However, we could not observe a noticeable decrease, or mild effect was observed in the premature forms of CPY protein bands upon *CUP1* overexpression in the mutant cells (Figs. 10*B*, S25, *D*–*F*, and S26*B*). Probably in these mutants, *CUP1* overexpression is insufficient as the inhibition of the translocation process is more (intense pp-CPY protein band). *CUP1*-overexpressing mutant cells as well as WT cells should contain less intracellular protein-free labile copper because they do not show copper-induced protein translocation defect or the defect was very low as compared to the *CUP1* untransformed cells.

Altogether, based on microscopic images with CS1 fluorescent dye, we believe that the mutant cells contain more intracellular labile copper pool which may associate with proteins such as Sec61 but with low affinity. However, the total cellular copper content in most of the mutant cells probably similar to the WT cells except for few mutants as suggested by the ICP-MS analysis (Fig. S27*N*).

Altogether, the above observations further suggest that the labile copper present in copper-sensitive histone mutants targets the activity of the Sec61 protein translocation channel.

### Sec61-mediated protein translocation process can be inhibited by cuprous (Cu^+1^) and cupric (Cu^+2^) forms of copper

**Figure 11:**
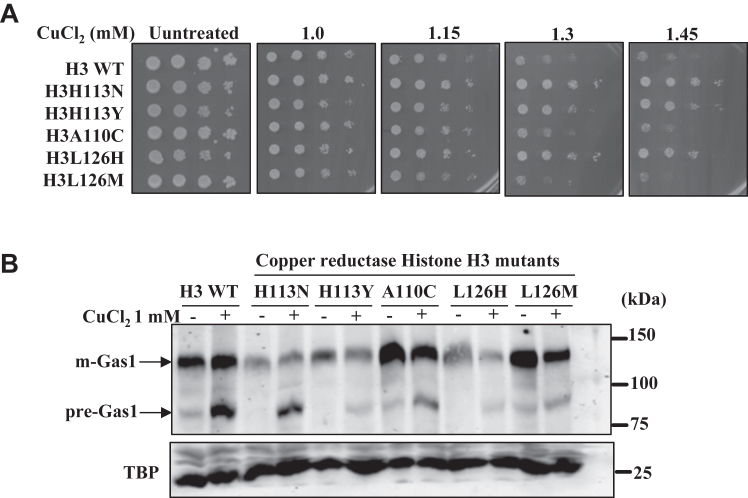
**Copper reductase mutants do not show any effect on copper-induced inhibition of protein translocation process.***A*, spot test assay of copper reductase H3 mutants and WT to test the growth phenotype in plates (spots from *left* to *right*, 10-fold serially diluted) containing different concentrations of CuCl_2_.2H_2_O (1.0, 1.15, 1.3, and 1.45 mM). UT means copper-untreated growth of cells in normal media. Cells were grown at 30 °C, and plates were scanned on the fourth day. *B*, immunoblot of Gas1-GFP–transformed copper reductase mutants and WT cells treated with 1 mM of CuCl_2_.2H_2_O for 2 h. TBP Western blot serves as a protein loading control. Gas1, glycophospholipid-anchored surface protein; TBP, TATA-binding protein.

Studies have shown that nucleosomal histone octamer acts as a copper reductase and converts the cupric form of copper to cuprous. The cuprous form of copper is utilized inside cells by the proteins of cytoplasm, mitochondria, and nucleus as cofactor, suggesting that copper must be available at the optimum concentration in the cuprous form. As we present evidence that copper inhibits the translocation activity of the Sec61 channel, we decided to test the effect of cupric and cuprous forms on the translocation process. To this end, we utilized the WT and copper reductase mutants of Histone H3. The mutants convert cupric to cuprous form at different rates; hence, they should contain different levels of cupric and cuprous forms. First, the growth of the cells was tested at different concentrations of exogenous copper chloride. The growth phenotype of WT and the H3 copper reductase mutants in the presence of copper correlates with their copper reductase activity, which means the mutants with better reductase activity are more sensitive to copper chloride and mutants that display weak reductase activity are less sensitive to copper (Fig. 11*A*). To gain more insight, we cotreated the cells with cysteine, histidine, glutathione, or BCS and tested their growth by spot assays. As expected, the growth development of all the strains was restored upon cotreatments (Fig. S27*A*).

Next, we wanted to test the effect of reductase mutants on the Sec61 protein translocation activity. WT and the copper reductase histone H3 mutants (H113N, H113Y, A110C, L126H, and L126M) were transformed with a plasmid to express Gas1 protein. Transformed cells were grown to 0.8 to 1.0 A_600_ and treated with different concentrations of copper chloride (1.0, 1.5, and 2.0 mM). Extracts were prepared, and Western blotting was performed to analyze the mature and premature forms of the Gas1 protein. Analysis of Gas1 protein bands suggests that the degree of inhibition of the protein translocation process upon exogenous supply of copper in the reductase mutants is not very different from the WT cells (Fig. 11, *B*–*F*). However, for some reasons, we observed significant downregulation of Gas1 protein in the mutants that have lower copper reductase activity (H113N, H113Y, and L126H) in both the absence and presence of exogenous copper chloride. This observation suggests that probably cuprous form of copper may be required for optimum protein stability, translation, and translocation processes. Since the inhibitory effect of exogenous copper on the protein translocation process is very similar between reductase mutants and WT cells, we believe that both forms of copper could bind with Sec61 translocon channel but may be with different affinity. These observations may also suggest a shallow or no difference between the ratio of cuprous and cupric forms among mutants in the media in which cells were grown. Hence, the effect on the translocation process is similar in WT and reductase mutants. The ratio between mature and premature Gas1 is almost equal in all the mutants with lower reductase activity (H113N, H113Y, L126H) and better reductase activity (A110C and L126 M). Altogether, the above results suggest that an increase in intracellular labile copper pool (cuprous or cupric) may prevent the Sec61-mediated maturation of secretory proteins.

### Stress conditions can modulate the copper-induced protein translocation defect

To further identify the significance of copper-specific inhibition of the protein translocation process, WT yeast cells were grown in the presence of 1 to 2 mM of exogenous copper chloride at different stress conditions such as higher temperature, oxidative stress, DNA damage, fermentative, and nonfermentative growth phases. Yeast cells adapt and survive under various stress conditions by inducing specific gene expression programs. Certain stress conditions can epigenetically regulate the intracellular metal content, including copper, by affecting the expression of proteins that play role in the cellular metabolism of metals. To this end, we first tested the growth of cells in the above conditions in the presence of copper chloride. The growth in the above-stress conditions in the presence of copper chloride was severely compromised (Fig. S29*A*). Next, we tested the protein translocation process in the above treatments. We observed that temperature and oxidative stress conditions (menadione) do not increase or decrease the copper-induced effect on the protein translocation process (Fig. S29, *B*–*D*). The inhibition of protein translocation by copper in the absence and presence of these stresses was found to be the same. However, cotreatment with hydrogen peroxide (Copper + hydrogen peroxide) slightly decreased the copper-induced premature form of Gas1, increasing the mature form (Fig. S29, *B*–*D*). On the other hand, co-treatment with DNA damage condition (MMS treatment) slightly increased the copper-dependent inhibition (increase in premature Gas1) of the protein translocation process (Fig. S29*E*), suggesting that under DNA damage conditions, copper suppresses the growth of yeast cells by preventing the protein translocation process.

**Figure 12:**
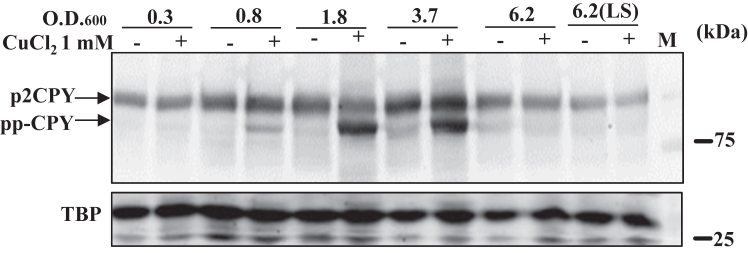
**Growth phases of yeast cells modulate copper-induced protein translocation defect.** CPY myc-tagged WT cells were grown from exponential to stationary phase. As indicated, cells were treated at different OD with 1 mM of copper chloride for 2 h. After treatment of cells, with CuCl_2_.2H_2_O (1 mM) at different A_600_ 0.3, 0.8, 1.8, 3.7, 6.2, 6.2 (LS) for 2 h, cells were harvested, extracts were prepared, and immunoblotting of CPY myc-tagged was performed. Minus (−) means untreated, and (+) means copper chloride treated. Western blotting with TBP served as a protein loading control. CPY, Carboxypeptidase Y; LS, later stationary; TBP, TATA-binding protein.

Next, we investigated the impact of copper on the protein translocation process during different growth phases. Yeast cells undergo various physiological changes, starting from the fermentation phase to nonfermentation and finally stationary due to drastic nutritional shifts. Interestingly, we observed that copper explicitly inhibits the maturation of secretory proteins (for example, CPY) during the fermentation phase (∼A_600_ 0.8–5.0) of the growth (Figs. 12*A* and S28*A*). However, after the fermentation phase beyond the A_600_ 5.0 (towards respiration phase), cells encounter multiple stress conditions such as osmotic, ethanol, pH, thermal, oxidative, and starvation which probably suppressing the effect of copper on cytosolic protein translocation process. To gain more insight, we grew the cells in media containing nonfermentative carbon sources and tested the effect of copper on the growth (Fig. S30*A*) and protein translocation process (Fig. S30*B*). Analysis suggests that copper treatment does not induce the protein translocation defect during the nonfermentative respiratory growth phase (Fig. S30*B*). To confirm the growth phases (fermentation and respiration) of WT yeast cells at different A_600_ (0.3–6.2), we examined the expression of two of the gluconeogenic genes, *FBP1* and *CAT8* (Fig. S30, *C*–*F*). We observed upregulation of these two genes only after A_600_ 6.0. This observation suggests that copper specifically prevents the protein translocation process during the fermentation growth phase and not during the respiration phase of the cells. Several factors probably contribute to the copper-dependent inhibition of the protein translocation process during the fermentation phase, such as intracellular bioavailability of copper, expression of chaperones and metallothionein, copper reductases, amino acid homeostasis, availability of zinc, *etc.*

## Discussion

Metal homeostasis is critical for many cellular processes. The export and import of metal ions through cell membranes are regulated through the specific protein transporters (44). Copper is an essential micronutrient trace element required by the metabolic enzymes as a cofactor to regulate crucial biochemical reactions in living organisms. As copper is malleable, ductile, durable, heat conductive, and exhibit antimicrobial activity, we use it in daily life in many forms: medicine, utensils, jewellery, and devices (45, 46, 47, 48). Multiple genetic and epigenetic factors govern cellular copper metabolism by regulating its export/import, sequestration, and compartmentalization to avoid toxicity. Other essential metal ions like zinc can influence cellular copper metabolism. While copper is vital for regulating life processes, deficiency or excess of copper can cause human diseases (49). For example, epigenetic and genetic dysregulation of copper metabolism results in pathological conditions such as Wilson's and Menkes's syndromes (50, 51, 52).

Moreover, a mouse model of Wilson's disease (Atp7b−/−) was found with metabolic dysregulations leading to increased glycolytic intermediates and components of the tricarboxylic acid cycle (15). Therefore, the cellular metabolism of copper must be tightly regulated. Epigenetic mechanisms are vital in maintaining health by regulating gene expression programs. Disruptions in epigenetics cause a variety of infectious and noninfectious diseases. Fundamentally epigenetic mechanisms include posttranslational modifications in histone proteins, chromatin remodeling, histone variants, and truncation of histone protein tails (53, 54, 55). However, the role of epigenetic mechanisms in copper metabolism is not entirely understood.

This study was carried out to understand the role of epigenetics and copper metabolism in the Sec61-mediated protein translocation process. Sec61 is a heterotrimeric, conserved protein complex located on the ER membrane, responsible for translocating secretory proteins from the cytosol to the ER lumen. After translation, secretory pathway proteins enter the ER through the Sec61 channel. Many of these proteins are synthesized in the cytosol in their inactive form. They are translocated to the ER, where they undergo folding and posttranslational modifications before they are transferred to the Golgi apparatus for further changes. Subsequently, the destination of the mature active form takes place. For example, the glycophospholipid-anchored surface protein (Gas1), a β (1, 3)-glucan elongase, is posttranslationally modified by the attachment of a glycolipid moiety called glycosylphosphatidylinositol at its C-terminal end. This modification facilitates the anchoring of the protein to the outer leaflet of the plasma membrane’s lipid bilayer (56, 57).

Similarly, CPY is synthesized as a pre-proenzyme, which enters the ER lumen through the Sec61 translocon, where the signal sequence is cleaved, producing proCPY (58, 59). The enzyme then folds *via* disulfide bond formation and glycosylation to form p1CPY, which is transported to the Golgi, where additional mannose residues are added, resulting in p2CPY mature form. Mutations in Sec61 have been identified causing cancerous growth. Inhibitors targeting the Sec61 channel activity have been developed; for example, combining mycolactone and bortezomib effectively kills patient-derived multiple myeloma cells by blocking Sec61 channel activity (60). In addition, apratoxin A and cotransin have been identified as specific inhibitors of the Sec61 channel that help to understand the mechanism of the protein translocation process (61). There is a significant demand for developing novel molecules that can specifically target the Sec61 channel activity.

Previously, by screening a library of yeast histone mutants with copper chloride, we identified the role of the N-terminal tail domains of histones in regulating *CUP1* expression (29, 62). We observed significant downregulation of *CUP1* and growth retardation upon exogenous supplementation of copper in yeast cells containing point mutations or truncations in the tail regions of histone H3 and H4. Another study provides evidence that copper can target the Sec61-mediated protein translocation process. Therefore, we hypothesized that copper-sensitive yeast histone mutants in which *CUP1* copper metallothionein is downregulated may contain higher concentrations of intracellular labile copper pool which can inhibit the Sec61-mediated protein translocation process. The ICP-MS analysis suggest that most of the copper-sensitive histone mutants contain same level of intracellular total copper concentrations as that of WT cells, indicating they may contain more labile copper pool. If this hypothesis is correct, the supplementation of exogenous copper in the growth media may increase the labile copper concentration in the mutants as the expression of *CUP1*, a high affinity copper-specific metallothionein, is downregulated and inhibit the Sec61 channel activity. The inhibition of Sec61 prevents the maturation of secretory proteins and causes a cytosolic increase in the accumulation of nonglycosylated immature forms in the mutants than WT cells.

Interestingly, we observed a significant increase in immature forms and a decrease in mature forms of Gas1 and CPY proteins in the mutants compared to WT cells upon exogenous supplementation of copper chloride. This observation suggests that epigenetic mechanisms play a vital role in copper metabolism because disruptions in histone proteins due to mutations or environmental factors can cause symptoms similar to Wilson's or Menkes's diseases (63). Supplementing a copper chelator, a few amino acids (histidine, cysteine), and glutathione in the growth media suppressed the copper toxicity, rescued the growth, and restored the Sec61-dependent protein translocation process. This observation indicates that amino acid homeostasis may play a critical role in the metabolism of trace elements, including copper. In addition, supplementation of zinc and overexpression of *CUP1* also restored the growth of cells because zinc may compete with copper for binding with proteins and overexpression of *CUP1* which encodes a Cup1 protein which can bind with the extra copper and hence not available to block the Sec61 channel. We also observed protein translocation defects in sod1- and cup2-deleted yeast cells upon exogenous copper treatment (data not shown), which further suggests that higher labile copper pool can target the Sec61 channel activity.

Utilizing available yeast mutants of Sec61 and subunits, we have demonstrated earlier that copper can inhibit the translocation activity of Sec61 channel. However, further studies to decipher the detailed mechanism of copper binding to the Sec61 channel are yet to be conducted. Several intriguing questions need to be answered to understand the mechanism. First, copper exists in cupric and cuprous forms, which one target the Sec61 channel or both the forms could bind but may be with different affinity. We tried to address this question by utilizing copper reductase mutants of histone H3. Recently, a study has shown the noncanonical role of histone H3 and H4 tetramer in copper reductase (26). The Cys110 residue located at the dimerization site of H3-H3 is required for the copper reductase activity of histone H3. To this end, we observed the same level of protein translocation defect upon exogenous copper chloride supplementation in the reductase mutants and WT cells. Investigations with other copper reductase enzymes, such as Fre1, may provide more insight about the mechanism. Second, Sec61 is an essential protein; hence, genetic manipulations are challenging. Third, as Sec61 is a complex of proteins, strategies to purify the complex need to be designed more carefully, as the addition of a suitable tag for protein purification may not be possible because it can disrupt the complex integrity and cause loss of translocation activity. Fourth, the low cellular abundance and transmembrane association of yeast Sec61 is another challenge to the purification of the complex. We tried to purify his-tagged Sec61 from yeast cells but could not succeed. Fifth, one could purify the recombinant Sec61 protein complex, which may or may not work due to the lack of posttranslational modifications and complex stability.

To gain more insight and to find the significance of the study, we thought to identify the impact of other stress forms: temperature, oxidative, DNA damage, fermentative, or nonfermentative growth phases on the copper-targeted protein translocation defects. To this end, we observed a very mild effect of temperature and oxidative stress conditions on the translocation process. On the other hand, DNA damage conditions significantly increased the copper-mediated inhibition of the protein translocation process. The above observations suggest that genomic instability can enhance copper toxicity by impairing the protein translocation process. Yeast cells undergo a massive reprogramming of gene expression and encounter a variety of stress forms: osmotic, pH, temperature, ethanol, and nutritional during fermentation, nonfermentation/respiration, and stationary growth phases. This information inspired us to examine the effect of copper on the protein translocation process during these growth phases. We observed that copper specifically inhibits the maturation of CPY protein during the fermentation phase, indicating that multiple stress conditions like osmotic, ethanol, pH, thermal, oxidative, and starvation can modulate the inhibitory effect of copper on the cytosolic protein translocation process.

**Figure 13:**
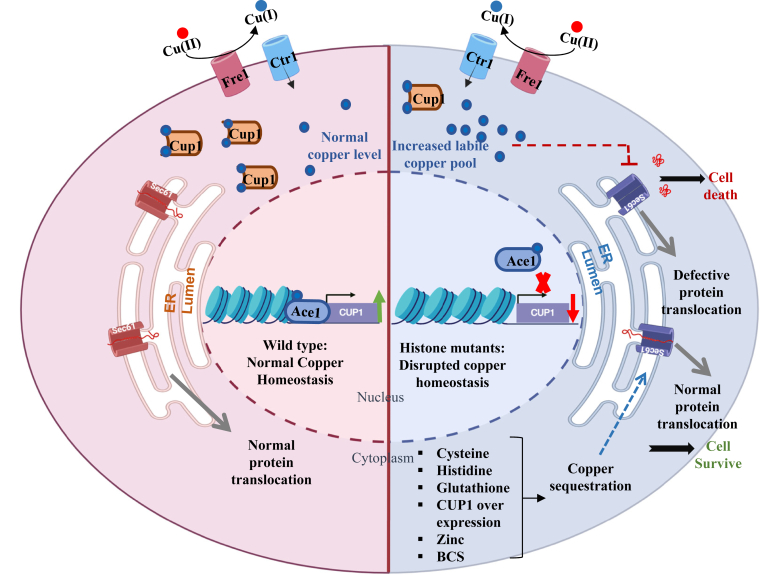
**Schematic representation for copper-mediated targeting of Sec61 translocation in copper-sensitive histone mutants and WT.** The proposed model shows the disruption of copper homeostasis due to mutations in histone tails, leading to defects in the Sec61-mediated protein translocation process. The WT cells can tolerate excess copper by increasing *CUP1* expression. Whereas the mutants with low levels of *CUP1* expression contains increased level of labile pool of copper inside the cells, accumulating precursor/immature forms of secretory proteins and causing cell death. The sequestration of intracellular excess labile copper by supplementation of cysteine/histidine/glutathione/zinc or upon over-expression of *CUP1* metallothionein restores the protein translocation process and rescues cell growth.

In summary (Fig. 13), this study, for the first time, provides a comprehensive mechanistic insight into epigenetic and metabolic regulation of copper homeostasis impacting Sec61-dependent protein translocation processes and has the potential to explore further novel ways for the treatment of human disorders of copper metabolism.

## Experimental procedures

### Yeast strains and growth conditions

The yeast mutants used in this study were grown in synthetic complete (SC) media (0.18 g of all amino acid mix with nutrients, 0.17 g of YNB (HiMedia), 0.5 g of ammonium sulfate (Sigma Aldrich), and 2 g of glucose with 2 g of agar [solid medium] or without it [liquid medium]) in 100 ml of distilled water or drop-out media, *that is*, SC-Leu/SC-His/Sc-Ura media at 30 °C under optimal growth conditions. The complete list of strains, plasmids, and primers used in this study can be found in the supplementary material, Table S1, *A*–*C*.

### Spot test assays and growth curve analysis

Saturated yeast primary cultures grown overnight were taken in equal amounts (absorbance at 600 nm = 1) and serially diluted four times by a factor of 10. These different dilutions were spotted on the Petri dishes containing solid growth medium (SC + Agar) with or without other compounds for treatments. After two to four days of incubation at the optimal temperature of 30 °C, the growth of cells that appeared on the plates was photographed using an HP ScanJet Scanner.

Growth curve analysis of cells in untreated and treatment conditions was carried out in the SC liquid medium. Briefly, a 96-well plate (Nunc Omnitray) was treated with different compounds and seeded with an equal number of cells (absorbance at 600 nm = 0.2) from a saturated overnight-grown yeast culture that had been used for secondary inoculation. Growth was measured using an automated BioTek plate reader, recording the absorbance at 600 nm every 30 min for 2 days.

### Protein extraction

The total cell protein from yeast cells was extracted using the TCA protein extraction method, as described earlier (30). Briefly, the yeast cells were harvested and stored at −80 °C after being washed with 20% TCA taken for total protein extraction. Cells were resuspended in 20% TCA for precipitation, an equal volume of glass beads was added, and vigorously vortexed to lyse the cells. The precipitated protein extract was centrifuged at high speed for 10 min at 4 °C. After discarding the supernatant, the pellet was washed using 0.5 M Tris–Cl (pH 7.5), followed by resuspension in loading buffer and heating at 100 °C for 10 min. To separate the insoluble debris from the supernatant, it was then centrifuged for 5 min at maximum rpm.

### Immunoblotting

The whole cell extract proteins were resolved using SDS-PAGE and transferred onto the nitrocellulose membranes using Bio-Rad transfer apparatus from Bio-Rad in a standard transfer buffer [Tris–Cl (pH 7.5), glycine, methanol, and SDS] for 90 min at 4 °C. Following the transfer, the membrane was blocked for 45 min at room temperature using 2.5% bovine serum albumin (catalog no.: MB083; HiMedia). The membrane was incubated in primary antibody for 90 min at room temperature, followed by subsequent washes with 1X TBST solution (Tris-buffered saline and Tween-20) containing Tris–Cl (pH 7.5), NaCl, and Tween-20. After washing, the membrane was incubated for 45 min at room temperature with a secondary antibody tagged with IR dye, followed by subsequent washes with 1X TBST solution to remove the nonspecific binding of the secondary antibody, and blots were scanned by utilizing the LI-COR infrared imaging machine. The primary antibodies used for immunoblotting were α-GFP (catalog no.: G1544; Sigma), α-TBP polyclonal antiserum raised in rabbit, α-Myc monoclonal antibody (catalog no.: MA1-980; Invitrogen), α-HA (catalog no.: 26183; Invitrogen), α-6X His (catalog no.: AB9108; abcam), α-Sec61 (gifted by Randy Schekman) and secondary antibodies were goat anti-rabbit IgG secondary antibody (catalog no.: A32734; Invitrogen) and goat anti-mouse IgG secondary antibody (catalog no.: 926–32210; Odyssey). Molecular weight protein loading markers (10–250 kDa) were loaded (BioRad catalog no. 1610374) in each western blots. Quantification of Gas1 and CPY bands was done by normalizing the signals of the mature and immature bands, and the ratio of Gas1 or CPY immature to mature forms were determined. The normalized intensities were added to obtain the total protein level of either CPY or Gas1. The formula used to evaluate the percentage of immature or mature protein relative to total protein level was {(normalized intensity of immature or mature protein/intensity of total protein) ∗ 100}.

### Transformation

pFA6a-13myc plasmid and primers specific for CPY C-terminal Myc tagging, a two-step PCR was used to create the template for tagging CPY at the C-terminus. Yeast cells were transformed using the purified PCR product as a template, and positive colonies were identified using SC-Trp plates (64). Briefly, yeast cells were made competent for transformation. The cells from the mid-log phase were washed with distilled water and 1× LioAc-TE buffer. This was followed by the transformation of the template into the cells using plate buffer (50% PEG, 1× LioAc-TE) and salmon ss-DNA. The cells were incubated at 30 °C for 45 min, followed by 42 °C for 15 min. This was followed by washing the cells with distilled water and spreading them onto the selection plate. The cell tagging was verified using a Western blot utilizing the α-Myc antibody. The competent cells were also transformed with the other plasmids using the same procedure described above. The same buffer (50% PEG, 1× LioAc-TE) and ss-DNA were used and incubated at 30 °C for 45 min, followed by 42 °C for 15 min. The cells were washed and spread onto the selection plates. The plasmid-transformed cells were maintained on selection media (SC-His/SC-Leu/SC-Ura). All the plasmids used in this study are listed in Table S1*A*.

### Microscopy

To visualize the labile copper pool inside cell, CS1 (Copper sensor-1; MedChemExpress, Catalog No.: HY-141511), a copper-specific fluorescent dye was used. To measure the intracellular labile pool of copper, overnight grown primary yeast cultures were set to 0.2 A_600_ for secondary culture. Cells were grown till A_600_ ∼1, harvested, washed twice with 1× PBS, and stained with membrane-permeable 2.4 μM of CS1 in the dark for 10 min at 30 °C. After incubation, cells were washed thrice with 1× PBS and resuspended in ∼25 μl of same and images were taken with Olympus FV-3000 confocal microscope. The ex/em wavelength used is 473−491/513−533 nm; DIC and CS1-fluorescence images were captured and analyzed using ImageJ software. Intensity measurements and plot analysis were performed on raw data processed with ImageJ. Controlled experiments were performed with CS-1 dye–unstained cells. Quantification of CS1 signals from images were done with two biological repeats (N = 2). The integrated density values of the fluorescent signals from two biological replicates were obtained from 50 cells from three different fields of images captured of samples prepared from each mutant, representing the relative fluorescent intensities. Graphs were plotted of relative fluorescent signals of each mutant and WT cells with mean ± SD.

### Inductively coupled plasma mass spectrometry

ICP-MS (iCAPQ-ICP-MS; Thermo Fisher Scientific) was used to measure the amounts of copper content inside the cells. Samples were prepared to assess copper content using established techniques with some modifications (65, 66, 67). The yeast cells were grown exponentially and harvested. The cells were extracted and washed thrice with ice-cold distilled water. The samples were boiled at 100 °C for 15 min, after which the supernatants were collected. To create a calibration curve for the measurement of copper content in samples, the stock solution of copper (Cu concentration: 1000 ppm) was purchased from Sigma Aldrich and was diluted serially to prepare calibration standards covering a wide range of concentration (0.1–150 ppb, n = 6). All of the samples and standards had 2.5% nitric acid and 5 ppb rhodium purchased from Sigma Aldrich as an internal standard. One of the calibration standards was monitored at regular intervals to check the overall stability of the instrument’s performance over the run time. For every batch of sample analysis, 10% of the samples were reanalyzed to check the precision of the analysis. A Certified Reference Materials for trace elements in water (TMDA-64.3, Environment Canada) was always analyzed after the calibration and just before the end of the run to check the accuracy of the analysis. Data were accepted when deviation in precision and accuracy were ≤±5% and ≤±10%, respectively.

### Statistical analysis

Every experiment was performed through at least three biological replicates. The student's *t* test was utilized to perform the statistical analysis and significance for yeast mutants with respect to WT. Images of western blots and microscopy were quantified by using ImageJ software. The measurements and calculations were done using M.S. Excel and Graphpad prism 8.

## Data availability

The data supporting the findings of this study are openly available within the article and supporting information.

## Supporting information

This article contains supporting information (37, 68, 69, 70, 71, 72).

## Conflict of interest

The authors declare that they have no conflicts of interest with the contents of this article.
