# Supplementary material for: Mutations in histones dysregulate copper homeostasis leading to defect in Sec61-dependent protein translocation mechanism in Saccharomyces cerevisiae
Source: J Biol Chem. 2025 Jan 8;301(2):108163. doi: 10.1016/j.jbc.2025.108163 (PMC11847117; doi:10.1016/j.jbc.2025.108163)
Supplement: Supplementary file [file mmc1.docx]

**Supplementary information**

**Figure S1**


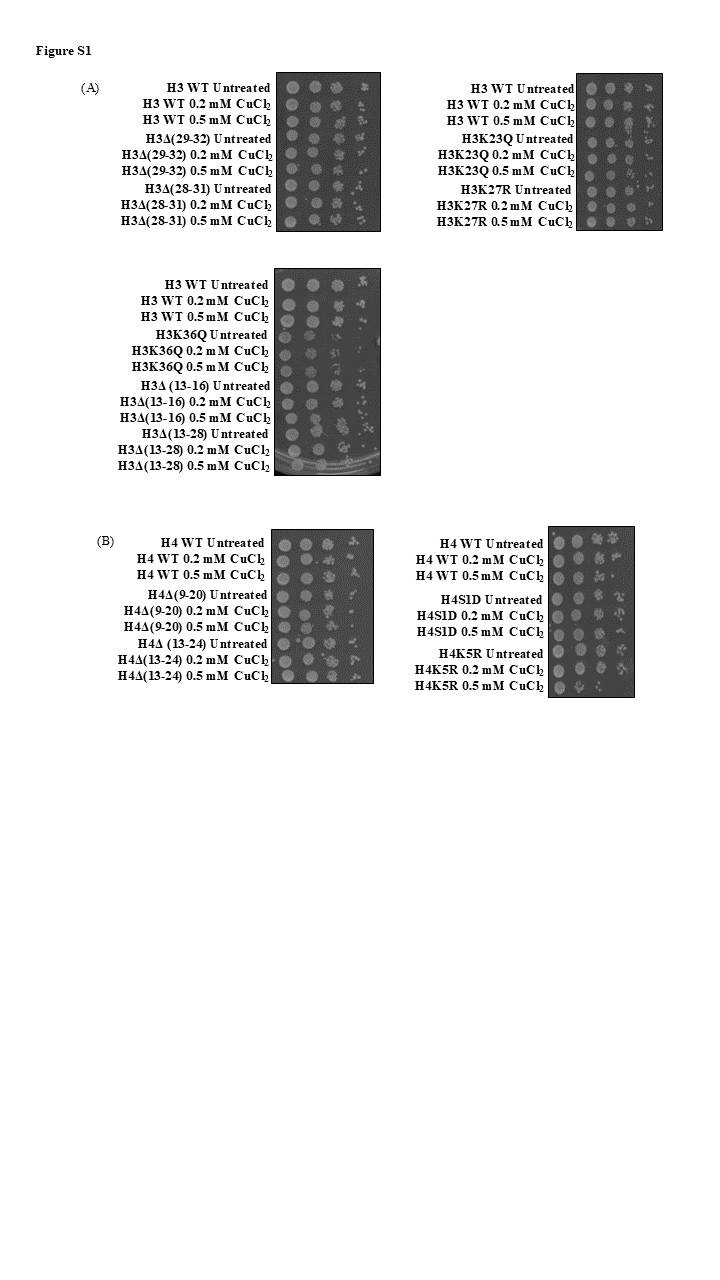


**(B)**

**(A)**

**
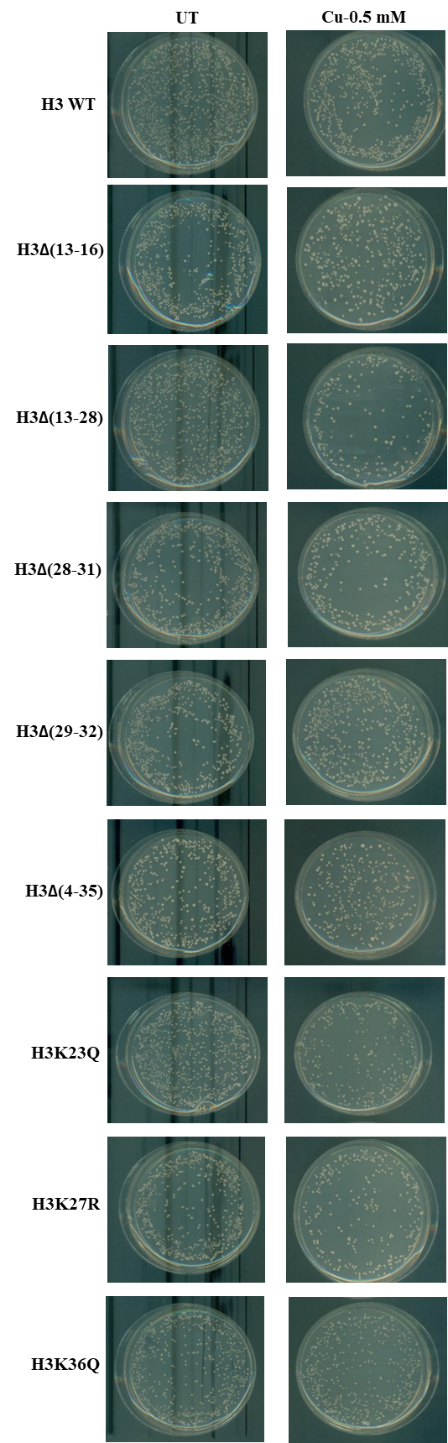

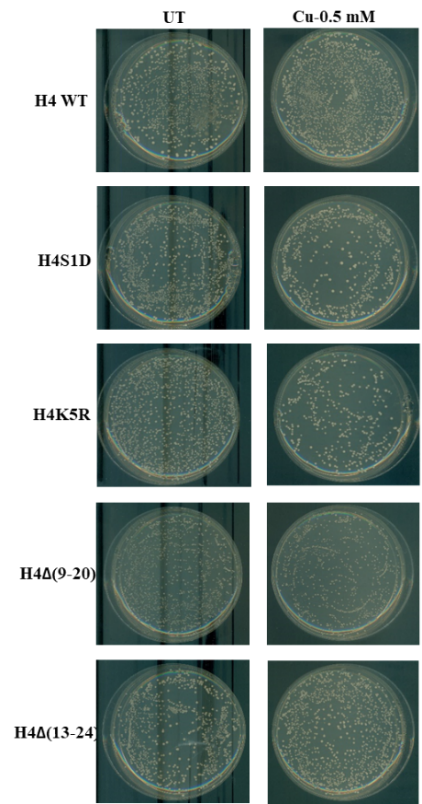
**

**(C)**

**(D)**

**Figure S1**


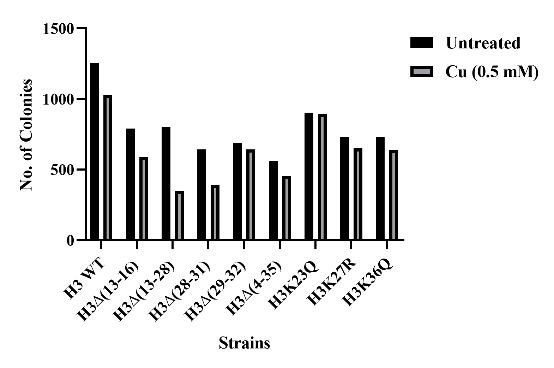


**(E)**


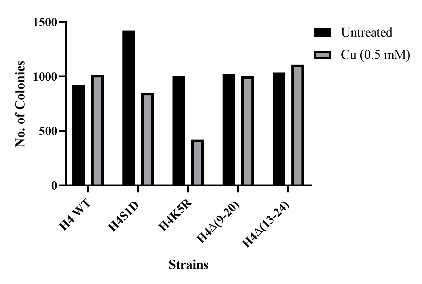


**(F)**

**Figure S1(A-F): Growth phenotype of copper-sensitive histone mutants in growth medium containing copper for 2 hours**: (A-B) Spot test assay of copper-sensitive H3 mutants and H4 mutants, respectively, along with wild-type. Cells were grown for 2 hours in liquid media without copper (UT, untreated) and in the presence of CuCl_2_.2H_2_O (0.2 and 0.5 mM), harvested, washed, and 10-fold serially diluted cells were spotted containing solid SC+Agar medium but without CuCl_2_.2H_2_O, incubated and images were taken after 72 hours. (C-D) CFU (colony forming units) of copper-sensitive histone H3 and H4 mutants, respectively, along with their wild types. In brief, cells were grown for 2 hours in liquid media without copper (UT, untreated) or in the presence of 0.5 mM of CuCl_2_.2H_2_O as indicated, harvested, washed, and equal OD_600_ of cells were spread in plates containing solid SC+Agar medium but without CuCl_2_.2H_2_O and kept at 30^0^C incubator. Images were taken after 72 hours. (E and F) Data indicates the number of colony-forming units appeared on the plates shown in ‘C’ and ‘D’ of wild type and mutants were counted and plotted. CFU experiments conducted minimum two times, images from only one of the repeats are presented here.

**Figure S1**

**(G)**


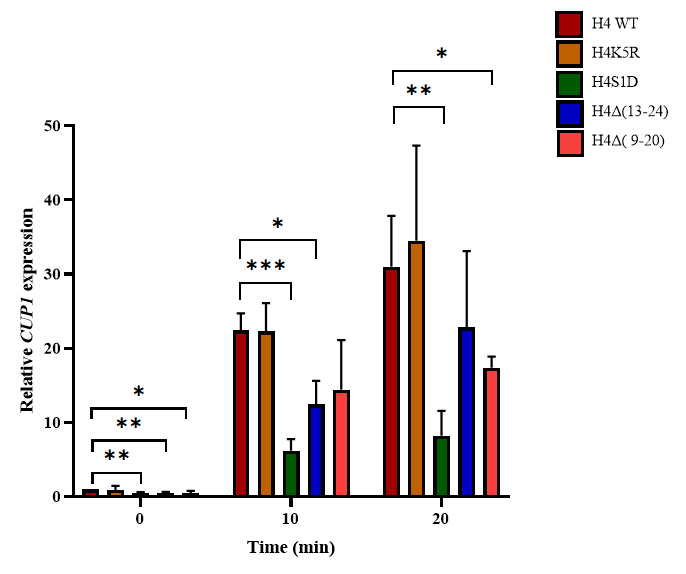


**Figure S1(G): The *CUP1* is downregulated in copper-sensitive histone H4 mutants:** Relative expression of *CUP1* in copper-sensitive histone H4 mutants. As indicated in the diagram above, secondary culture cells of wild type and the mutants were grown till the mid-log phase, harvested, and total RNAs were isolated.{Schmitt, 1990 #1}{Schmitt, 1990 #1} cDNAs were prepared upon normalization of RNA. Real-time PCR was performed to measure the *CUP1* levels, and *CUP1* transcript levels were normalized to internal control *ACT1* levels. Data are the means and standard deviations (SD) from three independent biological repeats (n = 3). Student's t-test statistical analyses were performed, and significant values are indicated as follows: *, P ≤ 0.05; **, P ≤ 0.005; ***, P ≤ 0.001.

**
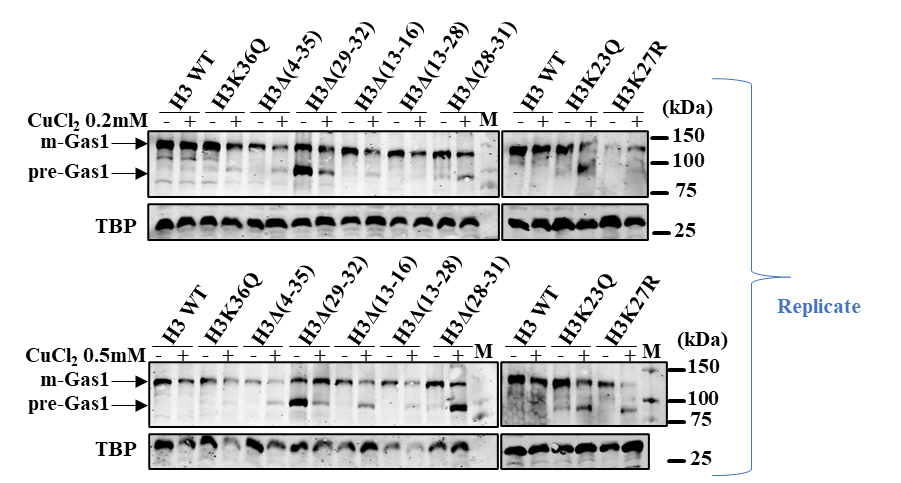

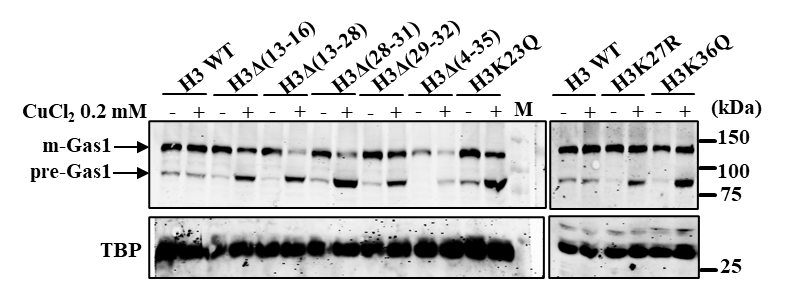
**

**(E)**

**(A)**

**Figure S2**


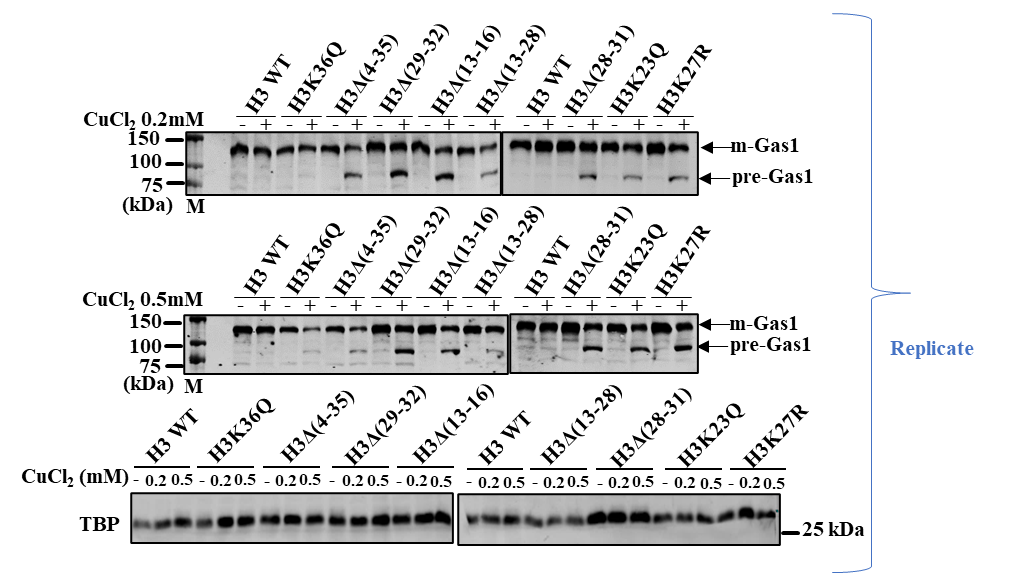


**(F)**

**(B)**

**(C)**

**
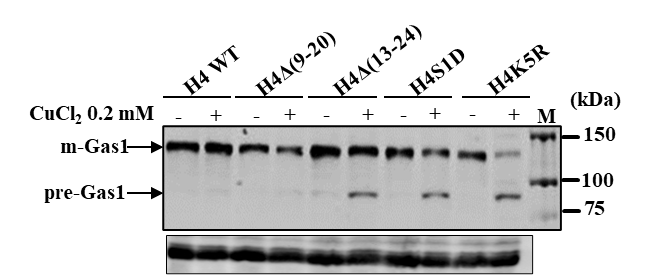
**

**(G)**

**(D)**

**
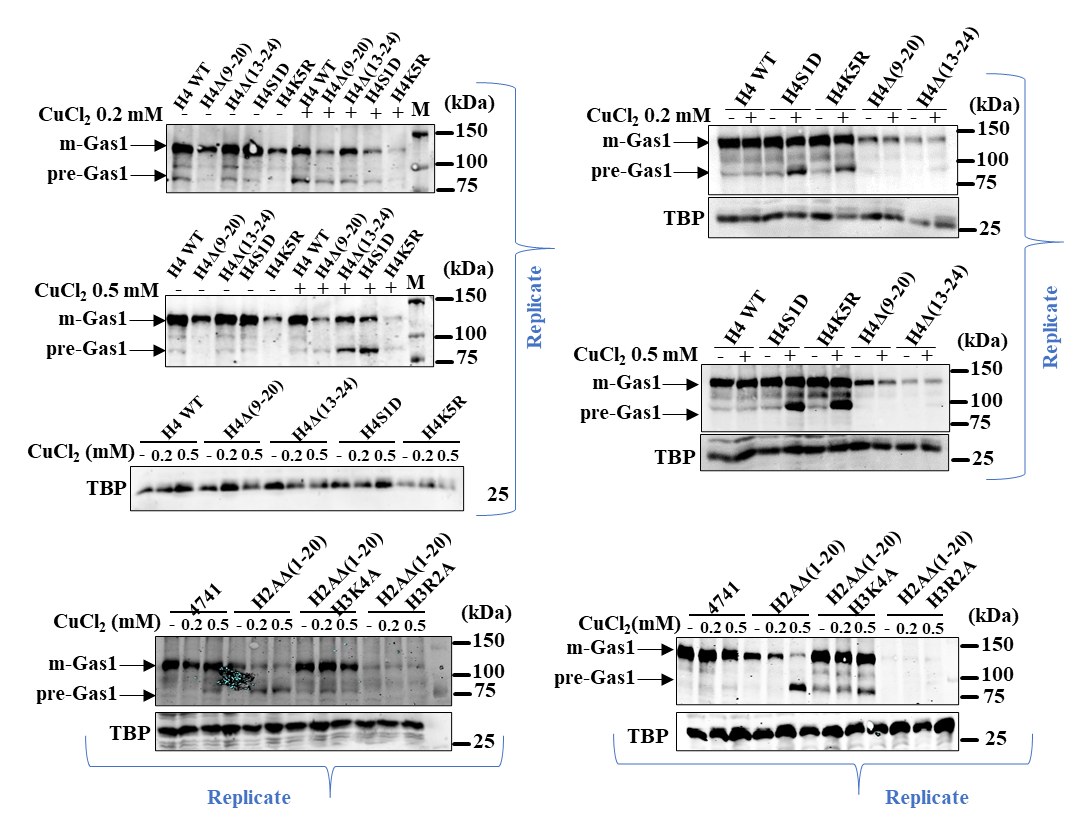
**

**(H)**

**(K)**

**(I)**

**(L)**

**(J)**

**(N)**

**(M)**

**Figure S2:** **Copper homeostasis dysregulation leads to accumulation of precursor Gas1.** Replicates of western blot data presented in Figure 1A-C. (A-F) Immunoblots of Gas1-GFP transformed copper-sensitive histone H3 mutants and wild-type cells treated with 0.2 mM and 0.5 mM of CuCl_2_.2H_2_O, respectively. (G-L) Immunoblots of Gas1-GFP transformed copper-sensitive histone H4 mutants and wild-type cells treated with 0.2 mM and 0.5 mM of CuCl_2_.2H_2_O, respectively. (M-N) Immunoblots of Gas1-GFP transformed copper-sensitive histone H2A mutants and wild-type cells treated with 0.2 mM and 0.5 mM of CuCl_2_.2H_2_O. TBP western blotting served as the protein loading control.


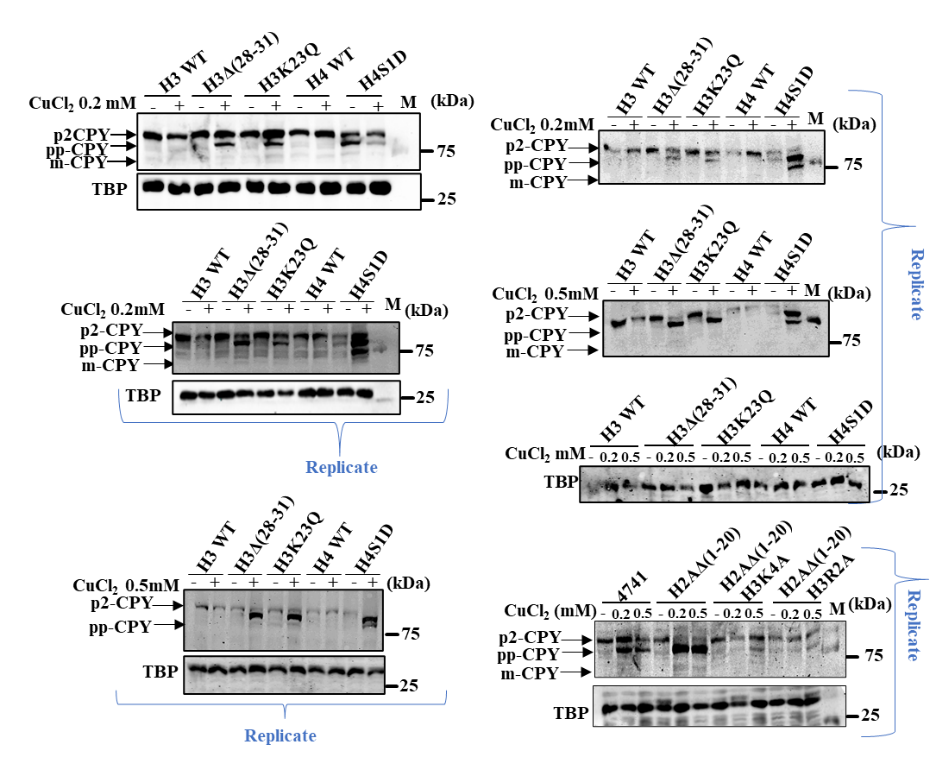
**
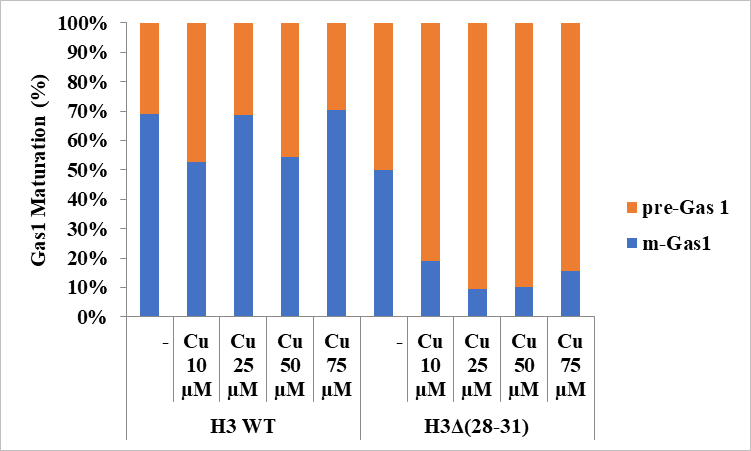
**

**(A)**

**(I)**

**(D)**

**Figure S3**

**(B)**

**(E)**

**
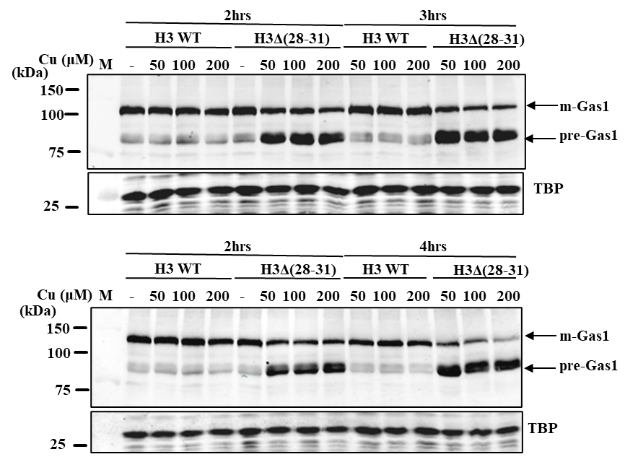
**

**(J)**

**(C)**

**(F)**

**(K)**

**
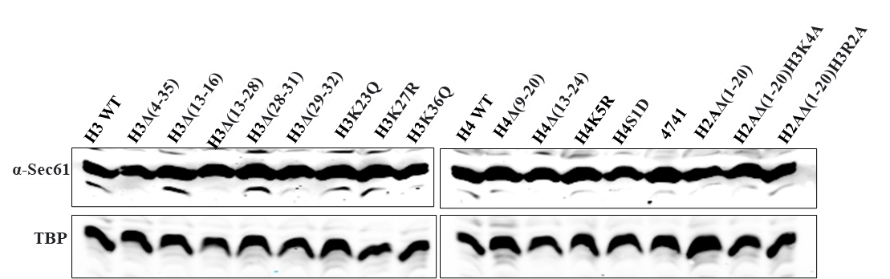
**

**(G)**

**
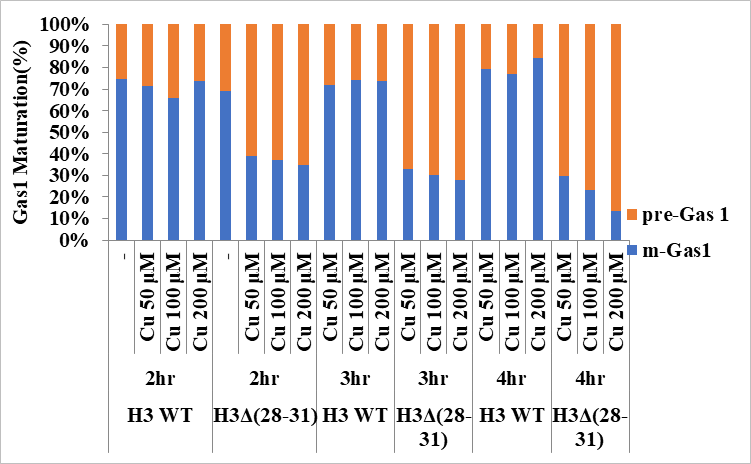
**

**(L)**

**
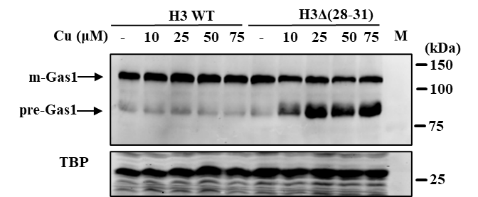
**

**(H)**

**Figure S3.** **Copper homeostasis dysregulation leads to the accumulation of precursor forms of Gas1 and CPY secretory proteins.** Replicates of western blot data presented in Figures 2A, and 2B. (A-E) Immunoblots of CPY Myc-tagged protein in copper sensitive histone H3 and H4 mutants and their wildtype cells treated with 0.2 mM and 0.5 mM of CuCl_2_.2H_2_O, respectively. (F) Immunoblot of CPY Myc-tagged in copper-sensitive histone H2A mutants and wildtype cells treated with 0.2 and 0.5 mM of CuCl_2_.2H_2_O, respectively. (G) Sec61 protein expression analysis by western blotting in wild type and the copper-sensitive histone mutants. (H) Immunoblot of Gas1-GFP transformed wild type and histone H3Δ(28-31) treated with different concentrations of CuCl_2_.2H_2_O (10, 25, 50, and 75 µM). (I) Quantification of western signals of blot ‘H’ data indicates the ratio of precursor and mature form of Gas1 in H3Δ(28-31) mutant w.r.t. wild type. (J-K) Immunoblots of Gas1-GFP transformed wild type and Histone H3Δ(28-31) mutant treated with different concentrations of CuCl_2_.2H_2_O (50, 100, and 200 µM) for 2-4 hrs. TBP western blotting served as the protein loading control. (L) Quantification of western signals of blots ‘J and K’ data indicates the ratio of precursor and mature forms of Gas1 in H3Δ(28-31) mutant w.r.t. wild type.

**Figure S4**


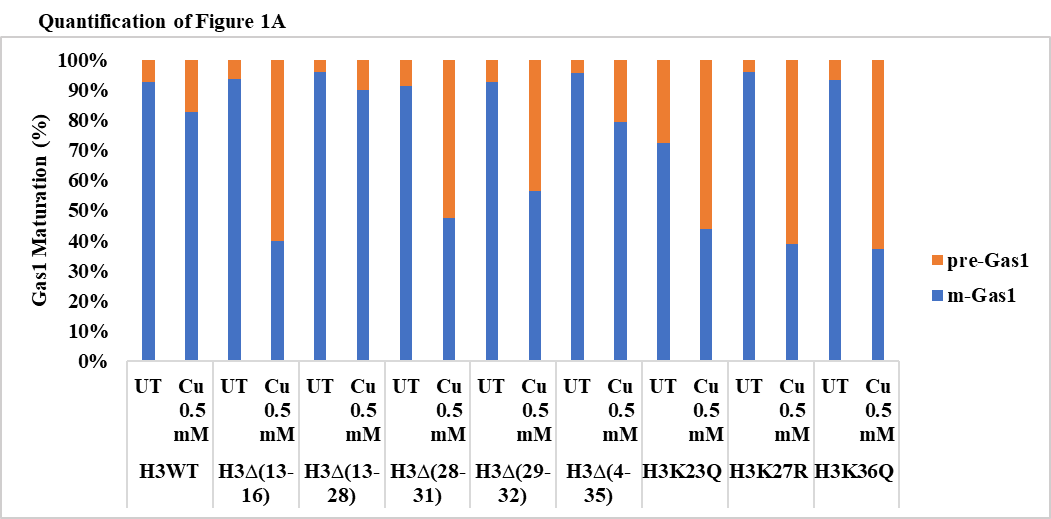


**(A)**

**Figure S4.** **Quantification of Gas1 in copper-sensitive histone mutants.** (A) Quantification of the western blots presented in Figure 1. Data indicates the ratio of precursor and mature form of Gas1 in histone H3 mutants w.r.t. wild type.

**Figure S5**


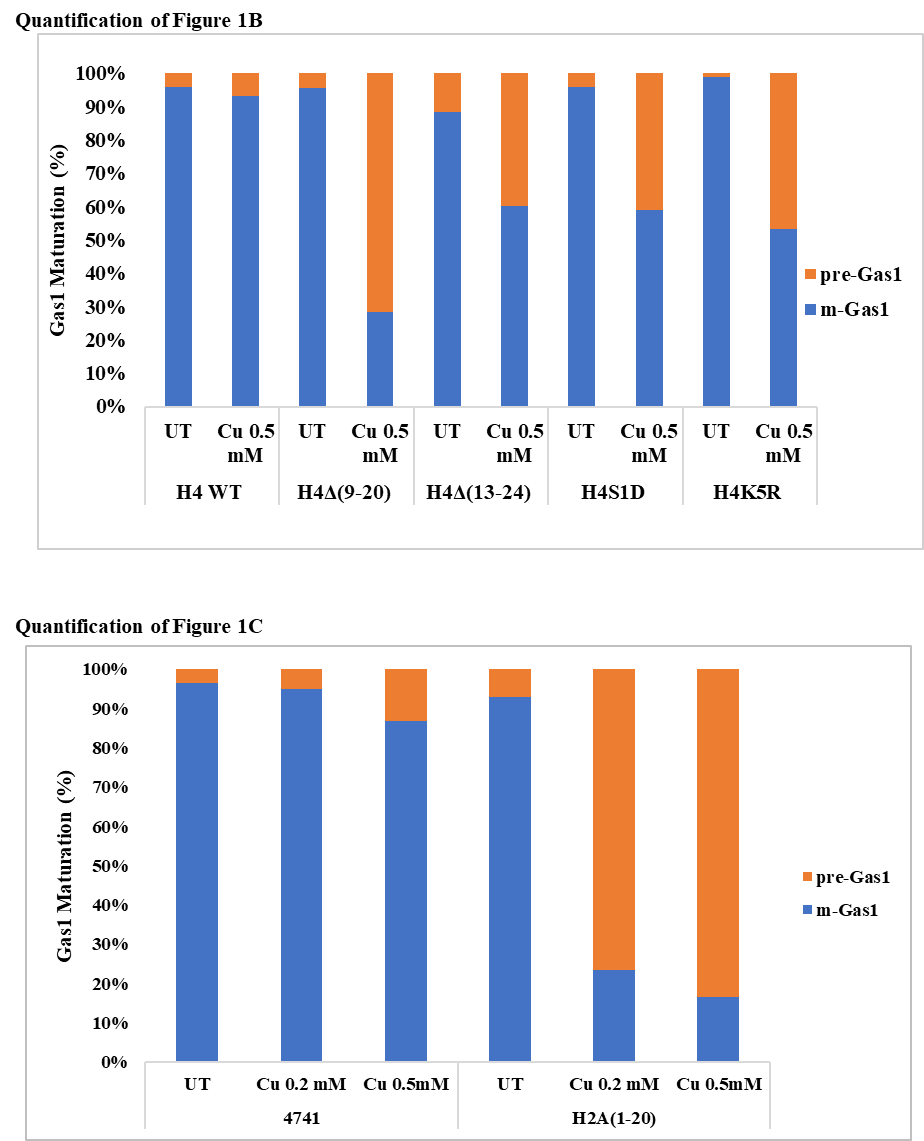


**(B)**

**(A)**

**Figure S5.** **Quantification of Gas1** **in copper-sensitive histone mutants.** (A-B) Quantification of the western blots presented in Figure 1. Data indicates the ratio of precursor and mature form of Gas1 in histone H4 and H2A mutants w.r.t. wild type.


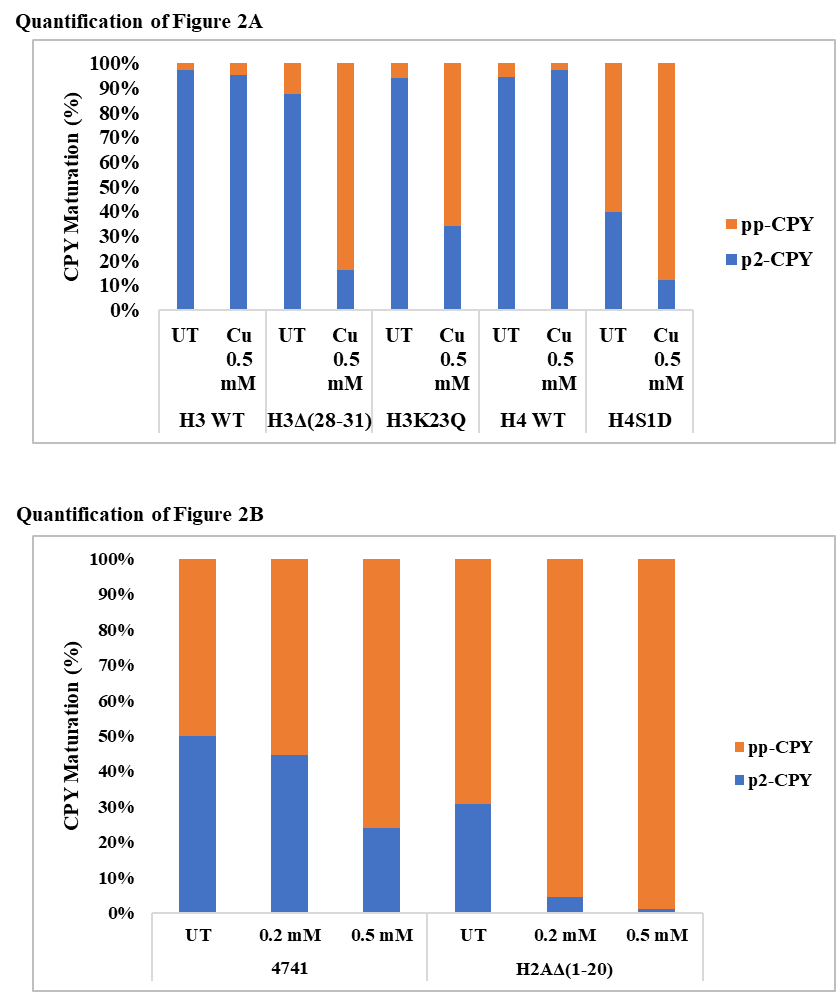


**(B)**

**(A)**

**Figure S6**

**Figure S6.** **Quantification of CPY in copper sensitive histone mutants.** (A-B) Quantification of the western blots presented in Figure 2. Data indicates the ratio of p2 and pp-form of CPY in histone H3, H4, and H2A mutants w.r.t. wild type.


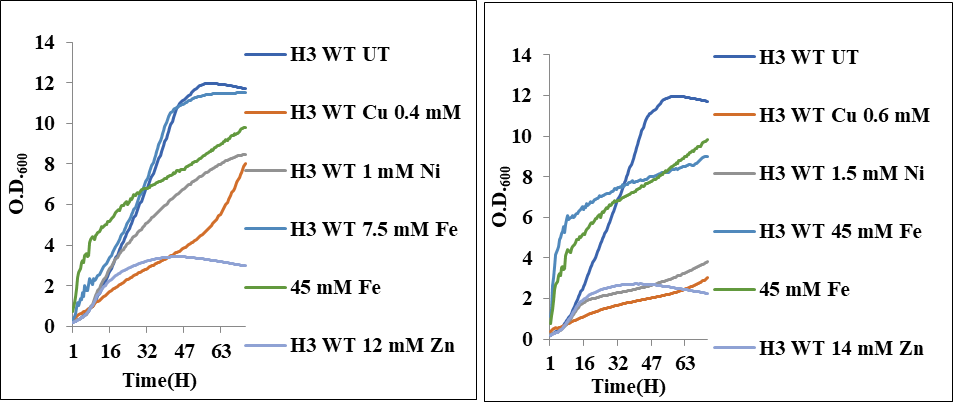

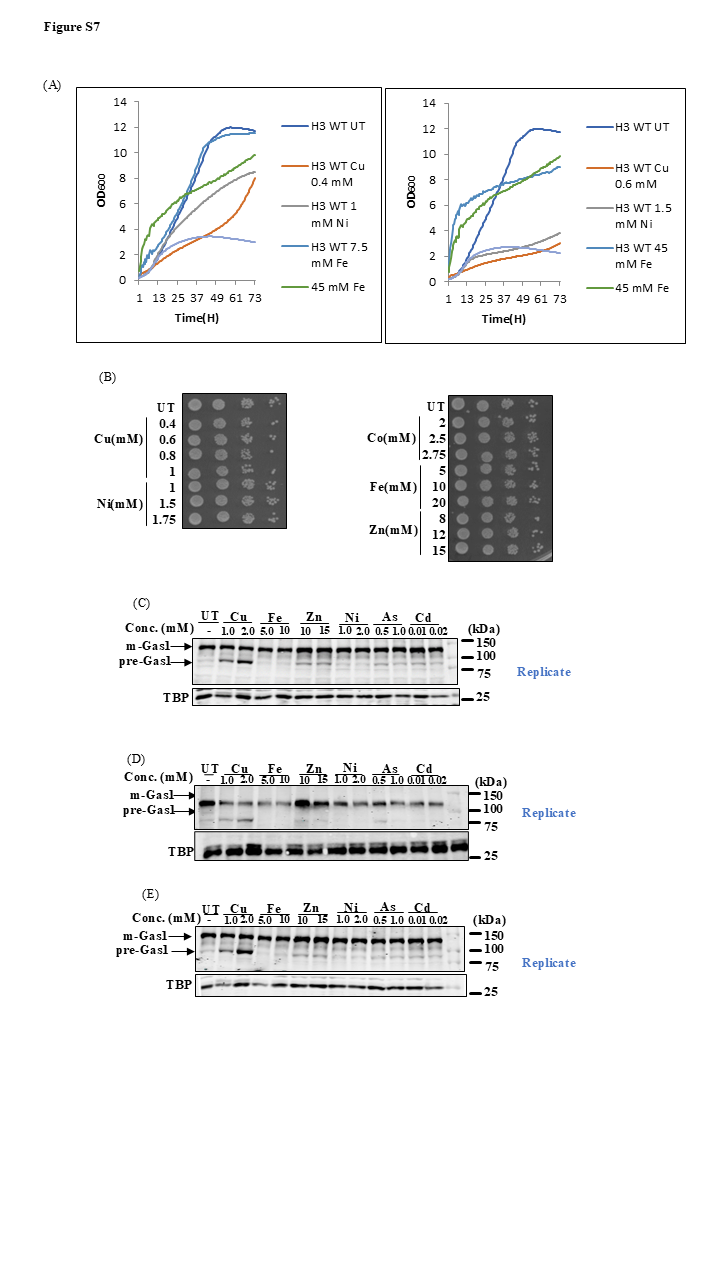


**(A)**

**Figure S7**


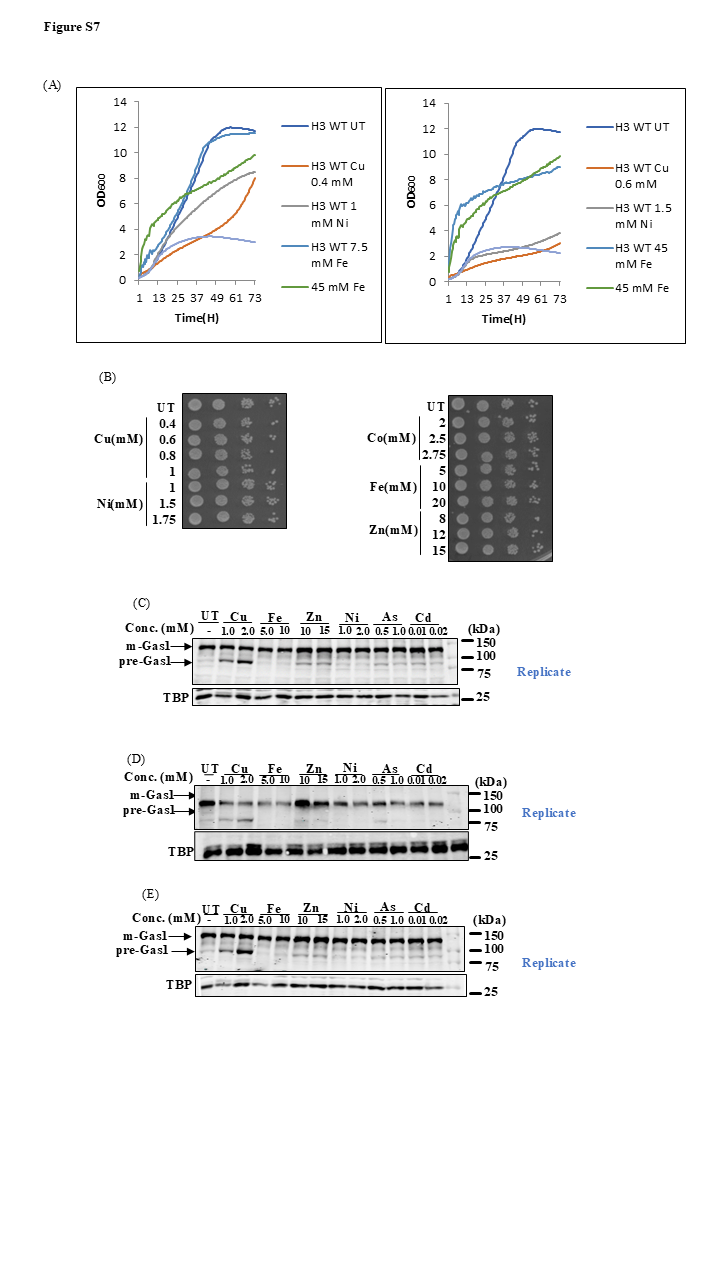

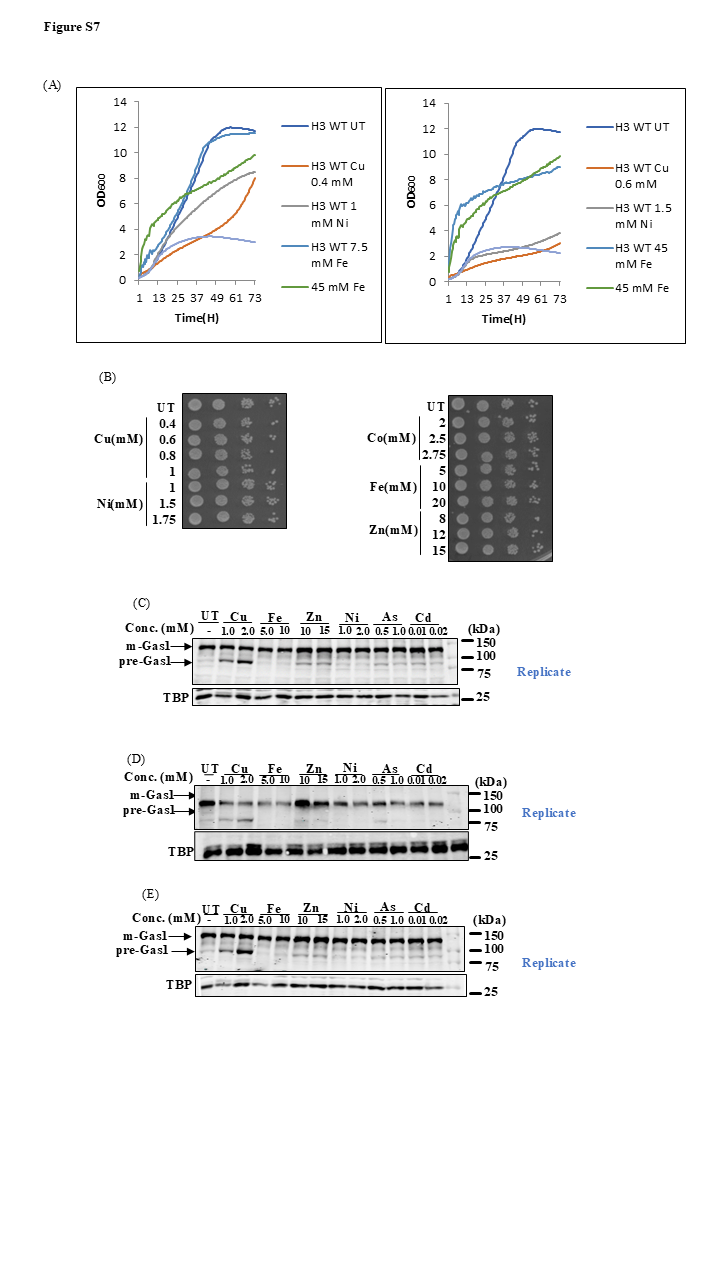


**(B)**

**
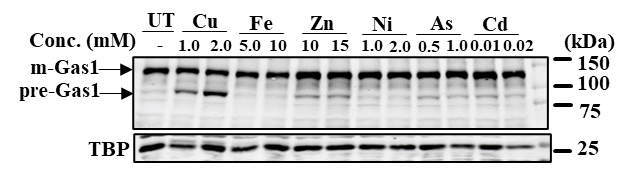
**

**(C)**

**
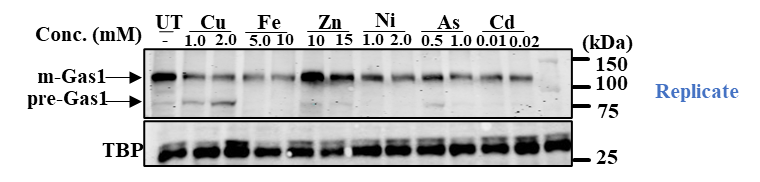
**

**(D)**

**(E)**

**
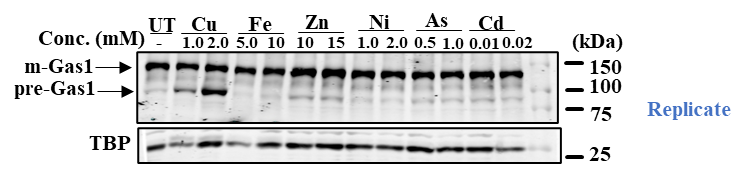
**

**Figure S7(A-E). Accumulation of precursor Gas1 is specific to copper homeostasis disruption.** (A) Growth curve analysis of wild-type yeast strain upon treatment with different metals. (B) Spot test assay of wild-type to test growth in plates containing SC+Agar solid medium, 10-fold serially diluted cells were spotted from left to right. First, cells were treated with different metals for 2 hrs in SC liquid media and then spotted onto plates having SC+Agar solid medium but without any exogenous metals. UT means metal untreated, growth of cells in normal media. (C-E) Immunoblots of Gas-GFP transformed wild-type cells treated for 2 hours with indicated concentrations of different metals (Copper, Iron, Zinc, Nickel, Arsenic, and Cadmium). TBP western blotting served as the protein loading control. H means hours.

**
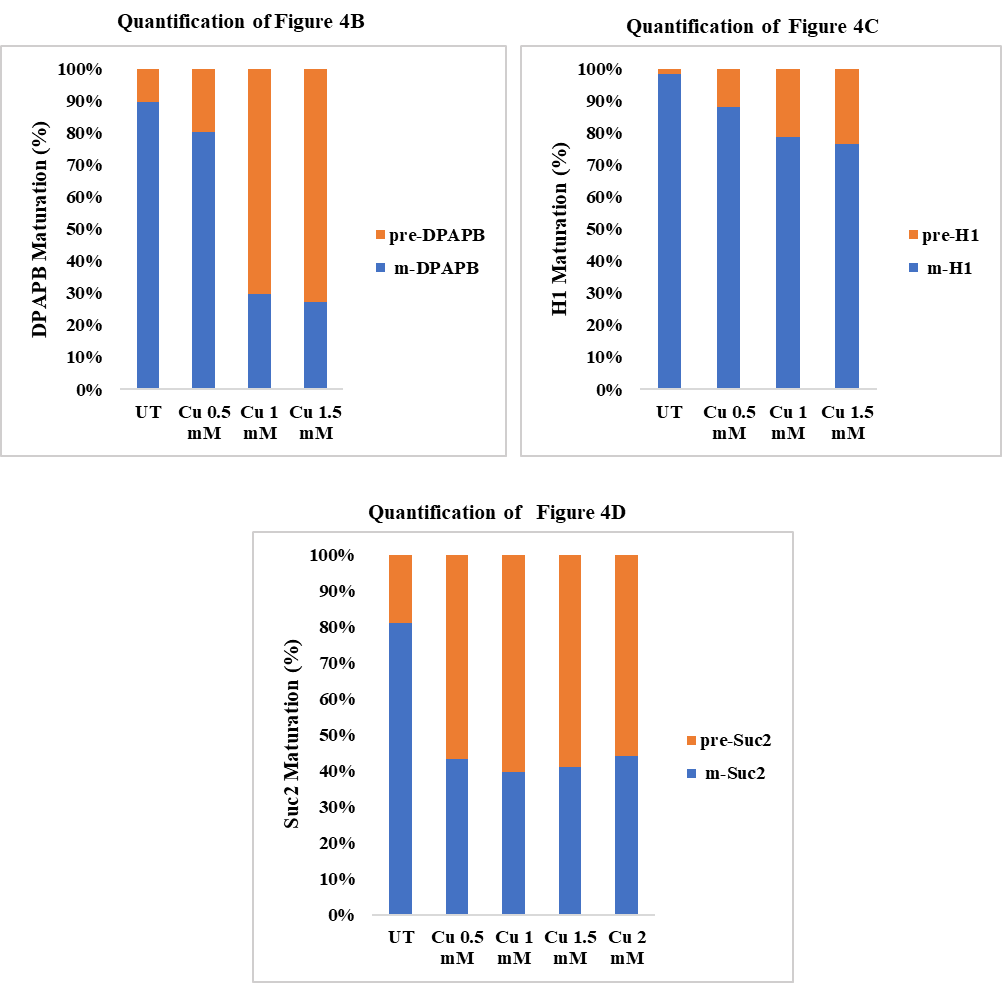
**

**Figure S7**

**(G)**

**(F)**

**(H)**

**(I)**

**
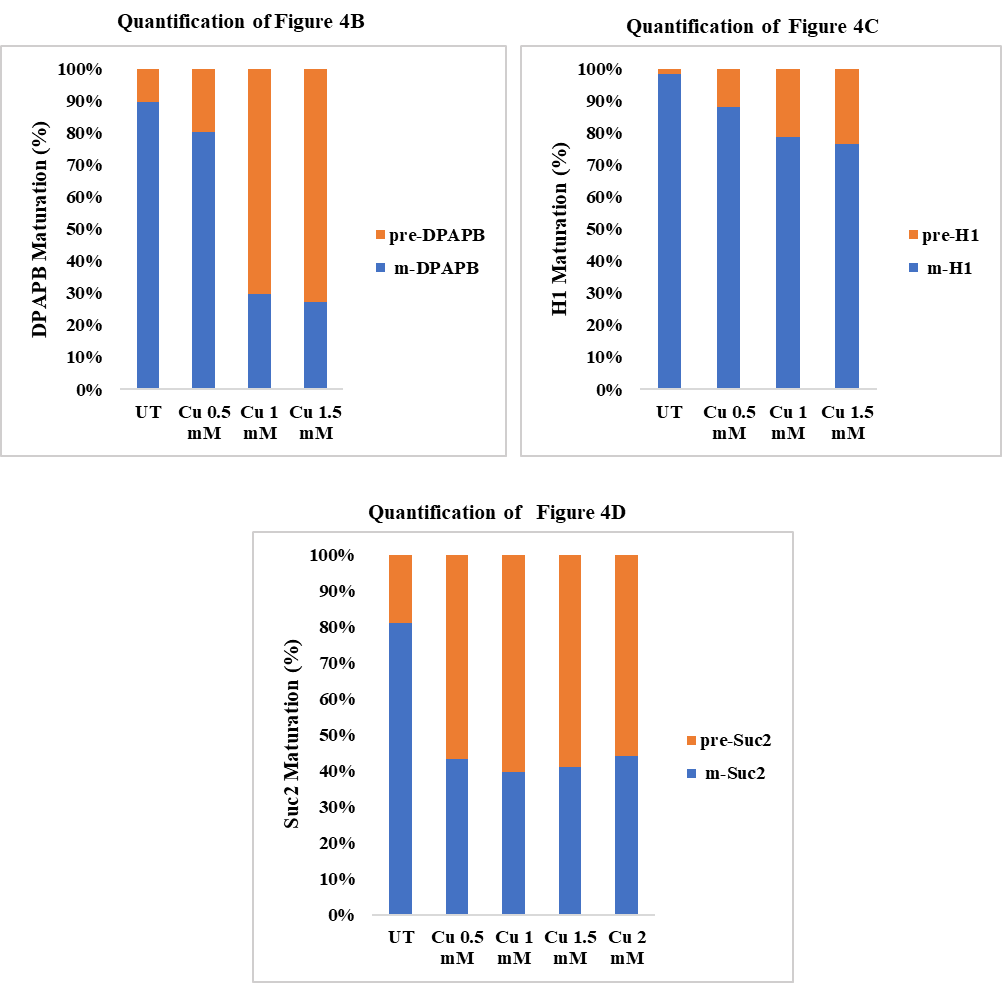

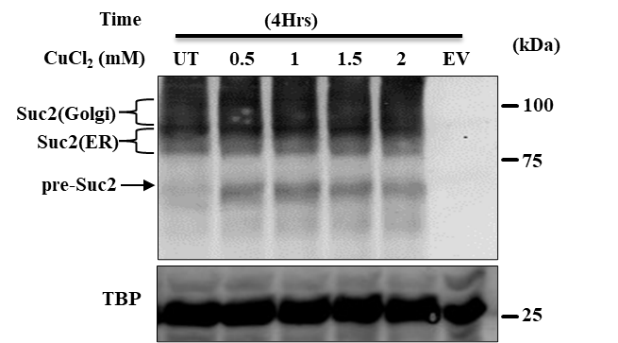
**

**Figure S7(F-I). Quantification of** **DPAPB, H1, and Suc2 in wild type and accumulation of precursor Suc2 upon treatment with copper.** Quantification of DPAPB, and H1 immunoblots presented in Figure 4B, and 4C, respectively. (F) Data indicates the ratio of m- and pre-form of DPAPB in wild type upon treatment with different concentrations of copper (0.5 mM. 1 mM, and 1.5 mM) (G) Data indicates the ratio of m- and pre-form of human asialoglycoprotein receptor H1 in wild type upon treatment with different concentrations of copper (0.5, 1.0, and 1.5 mM). (H) Immunoblot of Suc2-HA transformed wild type cells treated with different concentrations of CuCl_2_.2H_2_O (0.5, 1, 1.5 and 2 mM) for 4 hrs. TBP western blotting served as the protein loading control. (I) Quantification of western signals of blot ‘H’ data indicates the ratio of m (mature) and pre-form of Suc-2 in the wild type upon treatment with different concentrations of copper (0.5, 1.0, 1.5 and 2.0 mM) for 4 hrs.


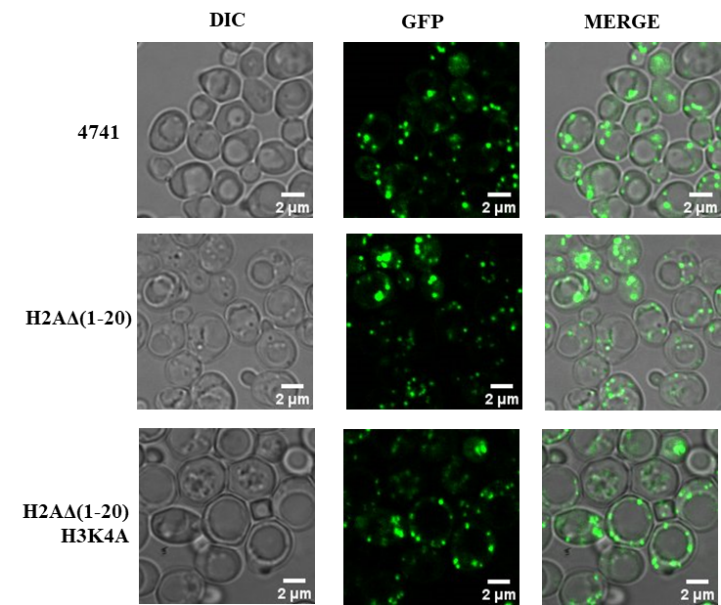

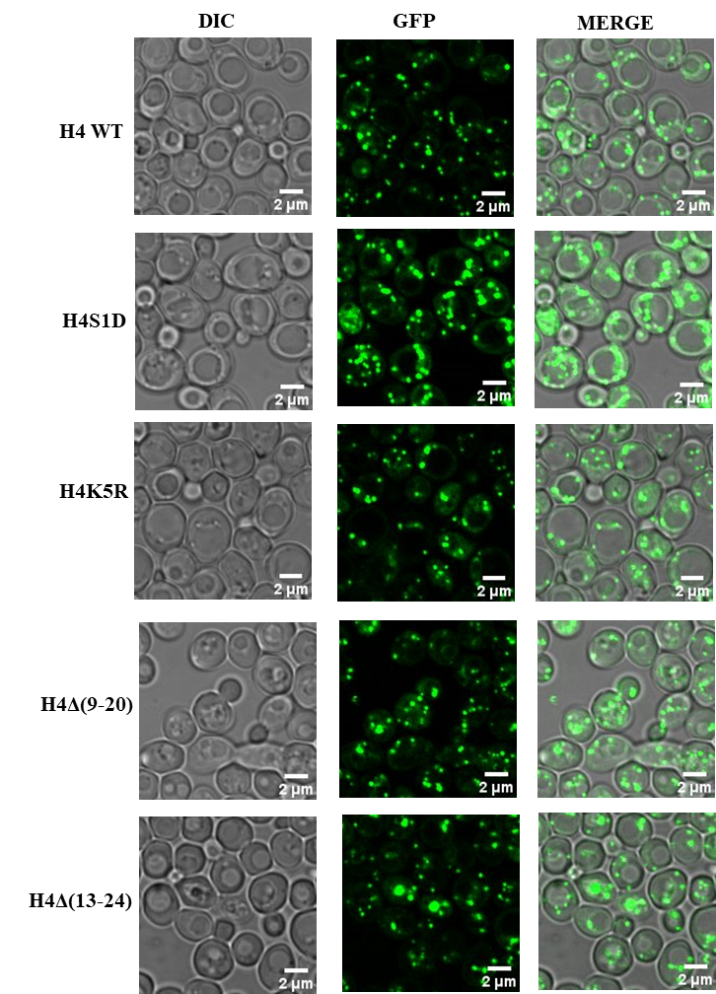


**(L)**

**Figure S7**

**(J)**

**(M)**


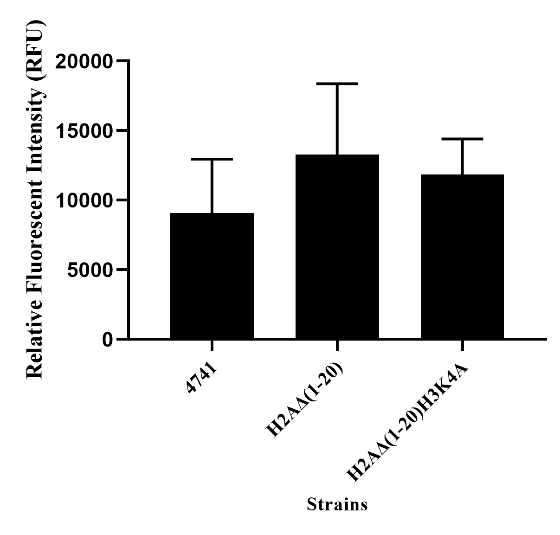


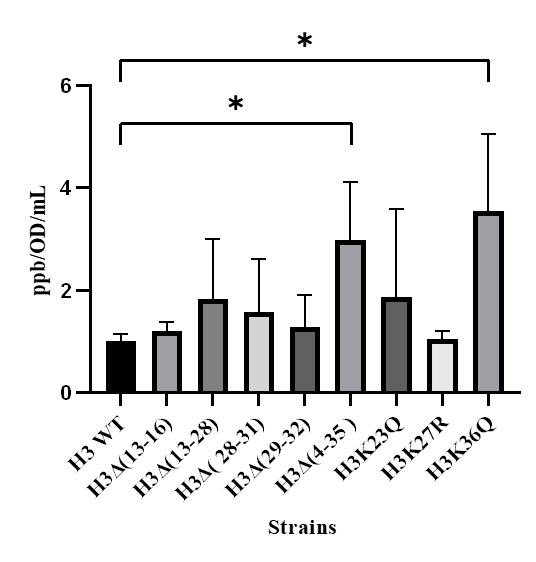


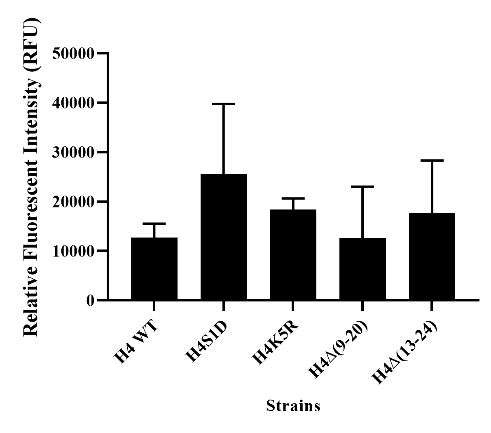


**(N)**

**(K)**

**Figure S7(J-N) Measurement of labile copper pool by CS1 fluorescent dye and total intracellular copper content by ICP-MS in mutants w.r.t. wild type yeast cells.** (J and L) Confocal microscopy images of yeast cells, histone H4 mutants, H2A mutants and wild type yeast cells stained with CS1 (coppersensor-1) probe. For control, CS1 unstained cells were used. (K and M) Data indicates the relative fluorescent intensity units (RFU) of images of H4 mutants shown in ‘J’ panel, and H2A mutants shown in ‘L’ panel w.r.t. wild type cells. Data represents the mean ± SD (from two independent biological repeats (n = 2). Fluorescent intensities were quantified from both the replicates by using ImageJ software from three different regions each consisting of 50 cells. (N) The bar graph represents the intracellular copper content in histone H3 mutants [H3Δ(13-16), H3Δ(13-28), H3Δ(28-31), H3Δ(29-32), H3Δ(4-35), and H3K23Q, H3K27R, and H3K36Q] and wild-type cells through ICP-MS analysis. Cells were grown till an O.D.600 ~ 1.0, harvested, washed twice with distilled water, boiled in distilled water for 15 min, centrifuged, and supernatants were collected to measure the copper contents by ICP-MS. Graphs plotted as mean ± SD (n=3). Statistical analysis was done using student's t-test of mutants with respect to wild-type (*P < 0.05).


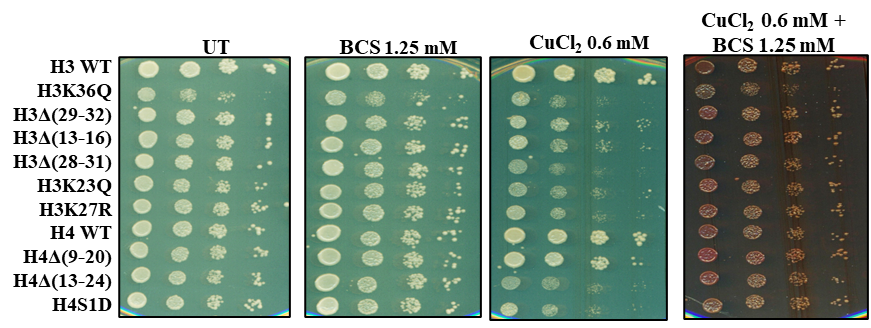


**(A)**

**Figure S8**

**
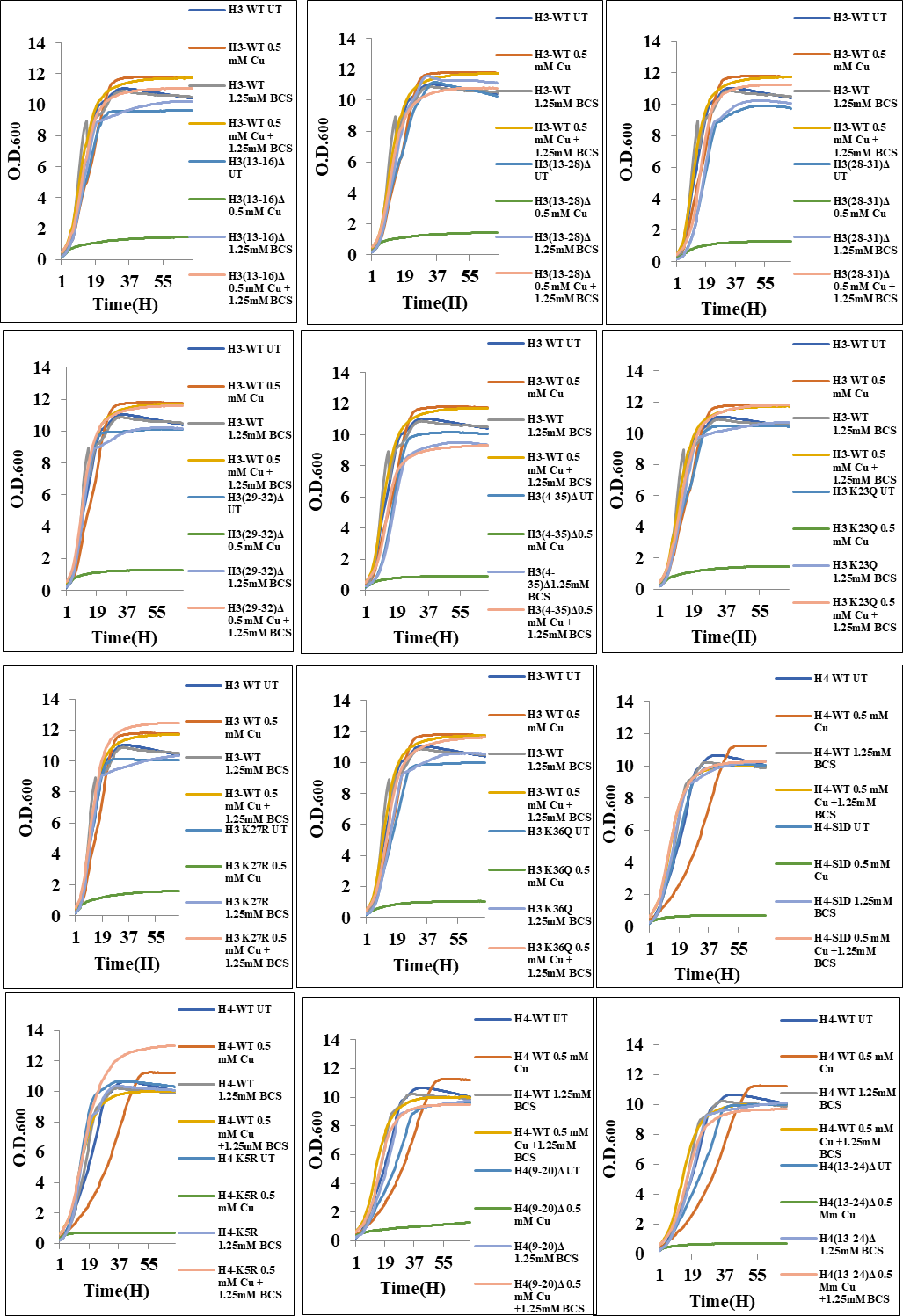
**

**(B)**

**Figure S8. Copper chelator, BCS suppresses the effect of copper on the growth of wild type and histone mutants.** (A) Spot assay of copper-sensitive histone H3 and H4 mutants, respectively, along with wild-type to test growth (spots from left to right, 10-fold serially diluted) upon treatment with copper, BCS, and co-treatment. UT means untreated, growth of cells in normal media. (B) Growth curve analysis of copper-sensitive histone H3 mutants and H4 mutants, respectively, along with wild-type to test their growth in liquid media in presence of 0.5 mM of copper, 1.25 mM BCS, and co-treatment.


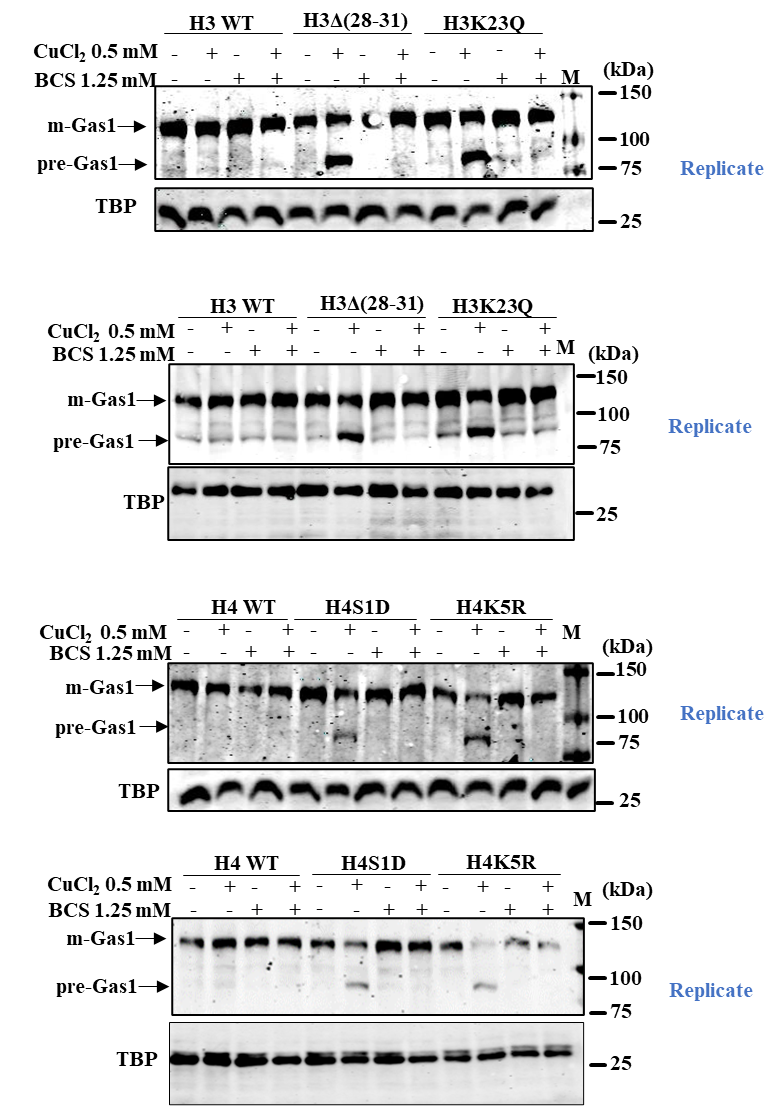

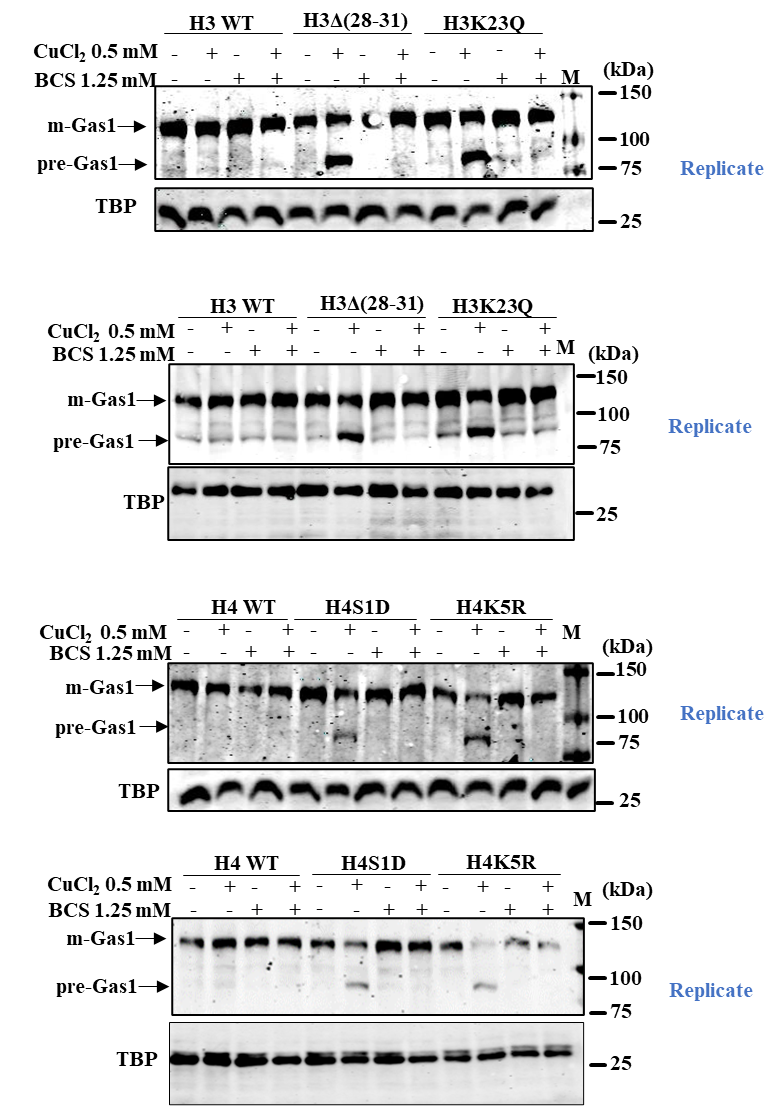


**(A)**

**(B)**

**Figure S9**

**
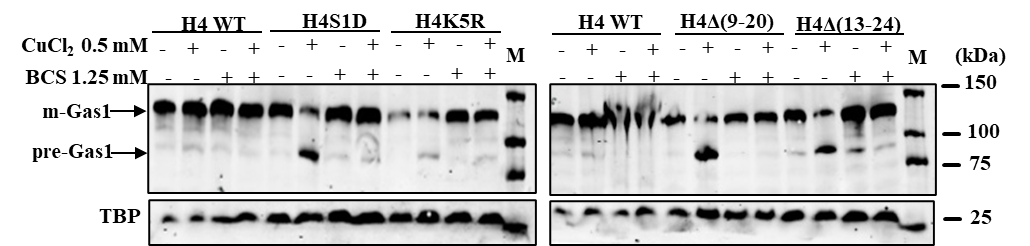
**

**(C)**


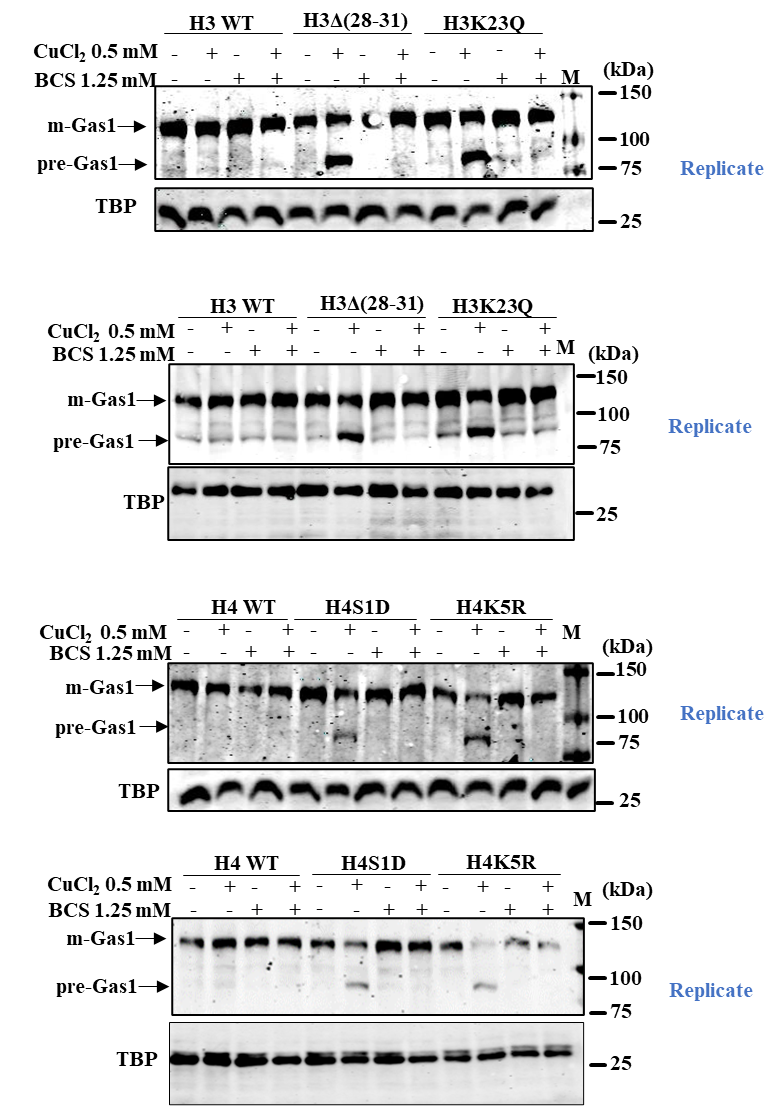


**(E)**

**(D)**


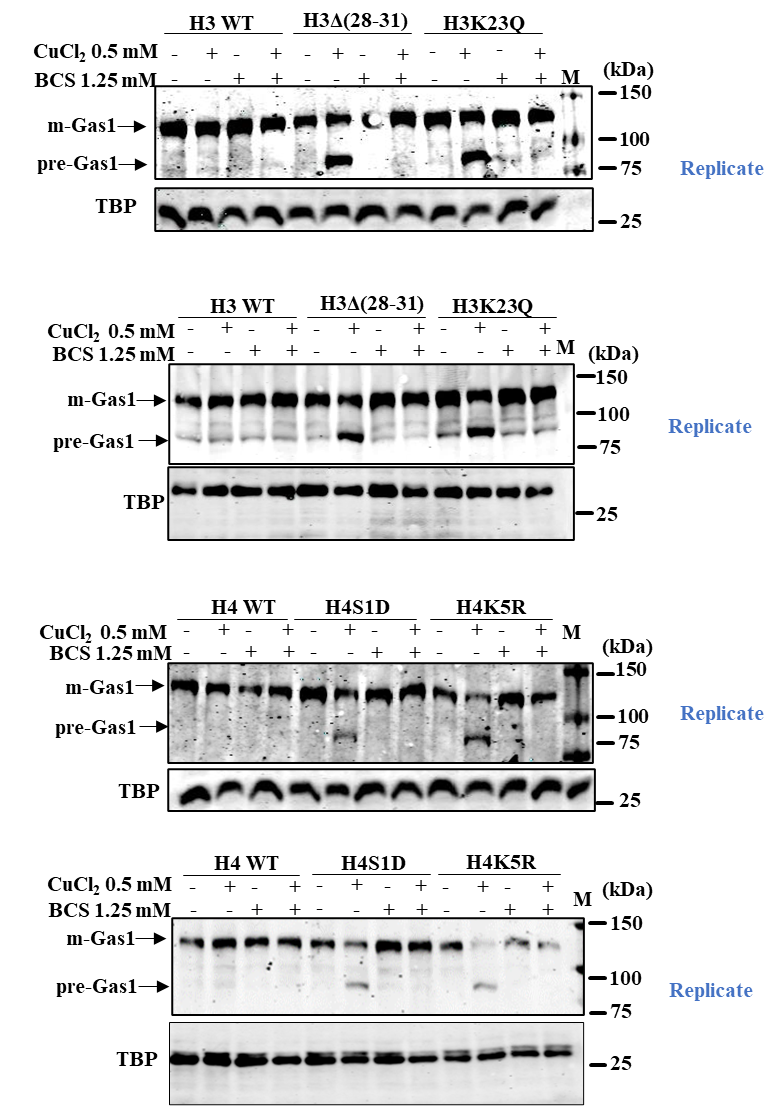


**Figure S9.** **BCS supplementation suppresses the copper effects on protein translocation process:** Replicates of western blots presented in Figure 5. (A-B) Immunoblot of Gas1-GFP in copper sensitive histone H3 mutants [H3Δ(28-31) and H3K23Q] and wildtype cells treated for 2 hours with CuCl_2_.2H_2_O (0.5 mM), BCS (1.25 mM) and co-treatments. (C-E) Immunoblot of Gas1-GFP in copper sensitive histone H4 mutants and wildtype cells treated with CuCl_2_.2H_2_O (0.5 mM), BCS (1.25 mM) and co-treatments. TBP western blotting served as protein loading control.


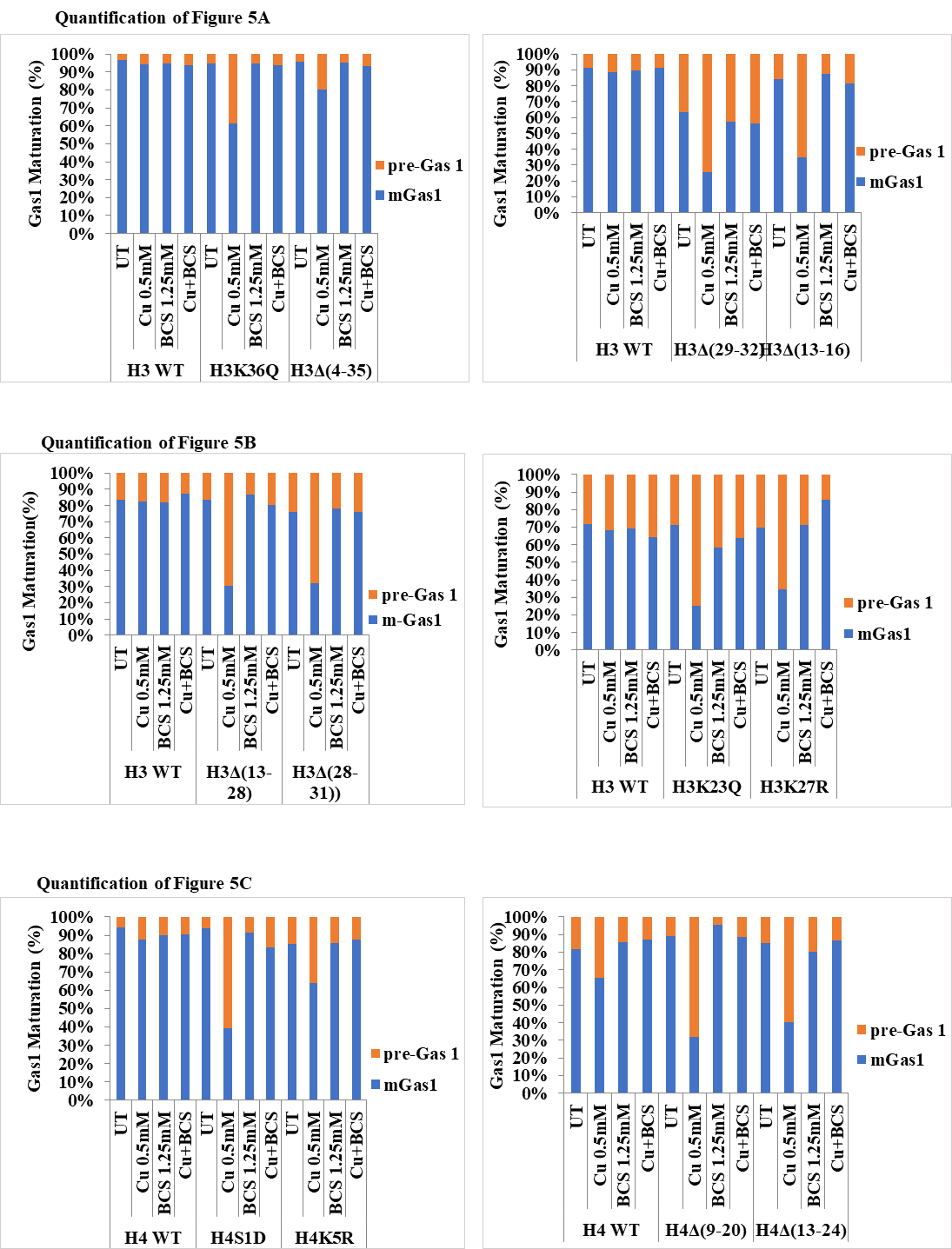


**(B)**

**(A)**

**Figure S10**

**Figure S10.** **Quantification of Gas1 secretary protein accumulation in copper-sensitive histone mutants of yeast cells.** (A-B) Quantification of the blots presented in Figure 5. Data indicates the ratio of precursor and mature forms of Gas1 in copper-sensitive histone H3 mutants w.r.t. wild type cells.


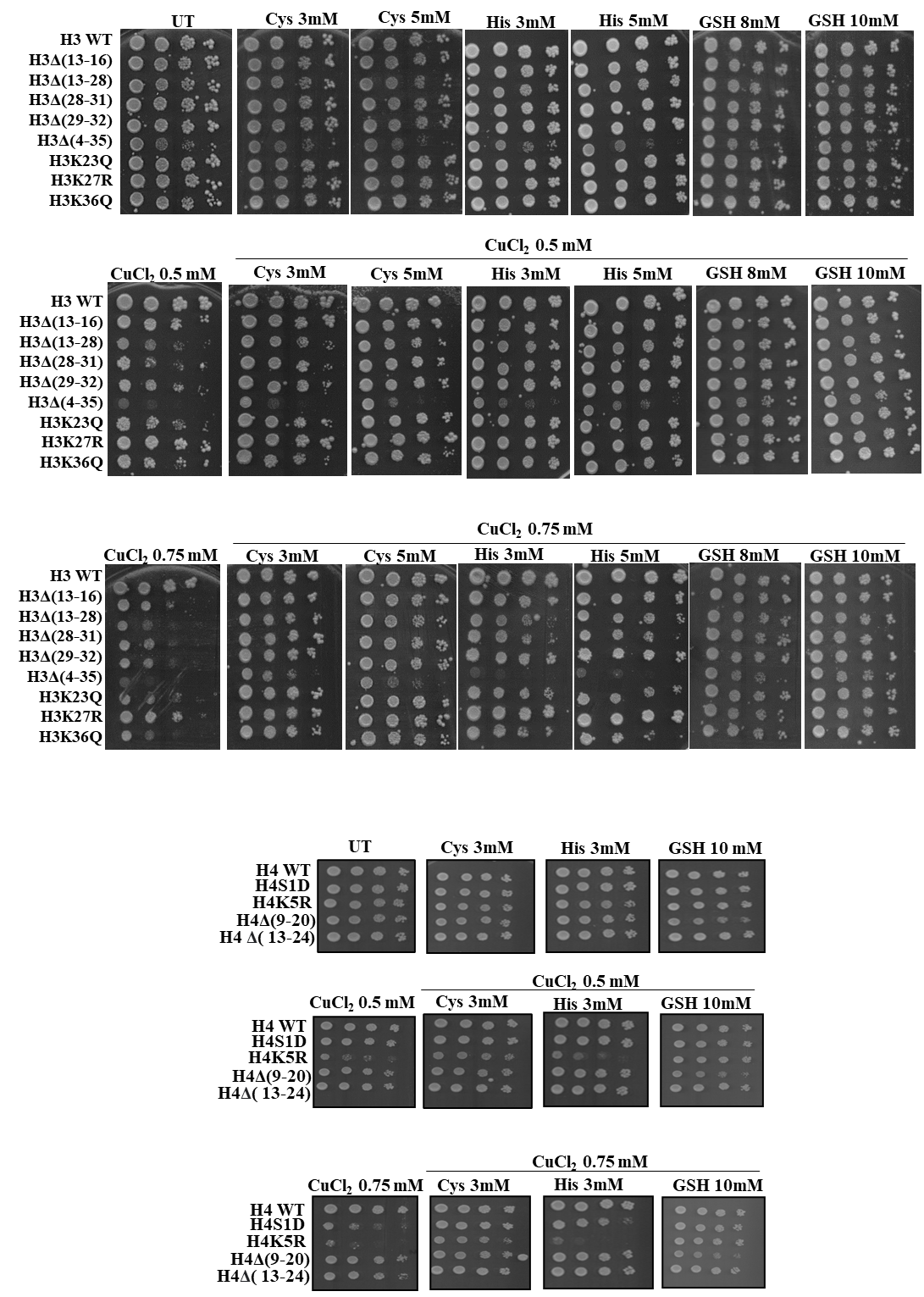


**(C)**

**(A)**

**(B)**

**(D)**

**Figure S11**

**Figure S11. The effect of copper on the growth of copper sensitive histone H3 and H4 yeast mutants is suppressed by supplementation of Cysteine, Histidine and GSH.** (A-D) Spot test assay of copper-sensitive histone H3 and H4 mutants, respectively, along with wild-type cells to test their growth in solid SC growth medium. The cells were spotted from left to right, 10-fold serially diluted on the plates containing copper, cysteine, histidine, GSH, and co-treatments as indicated above. UT means untreated, growth of cells in normal growth media.


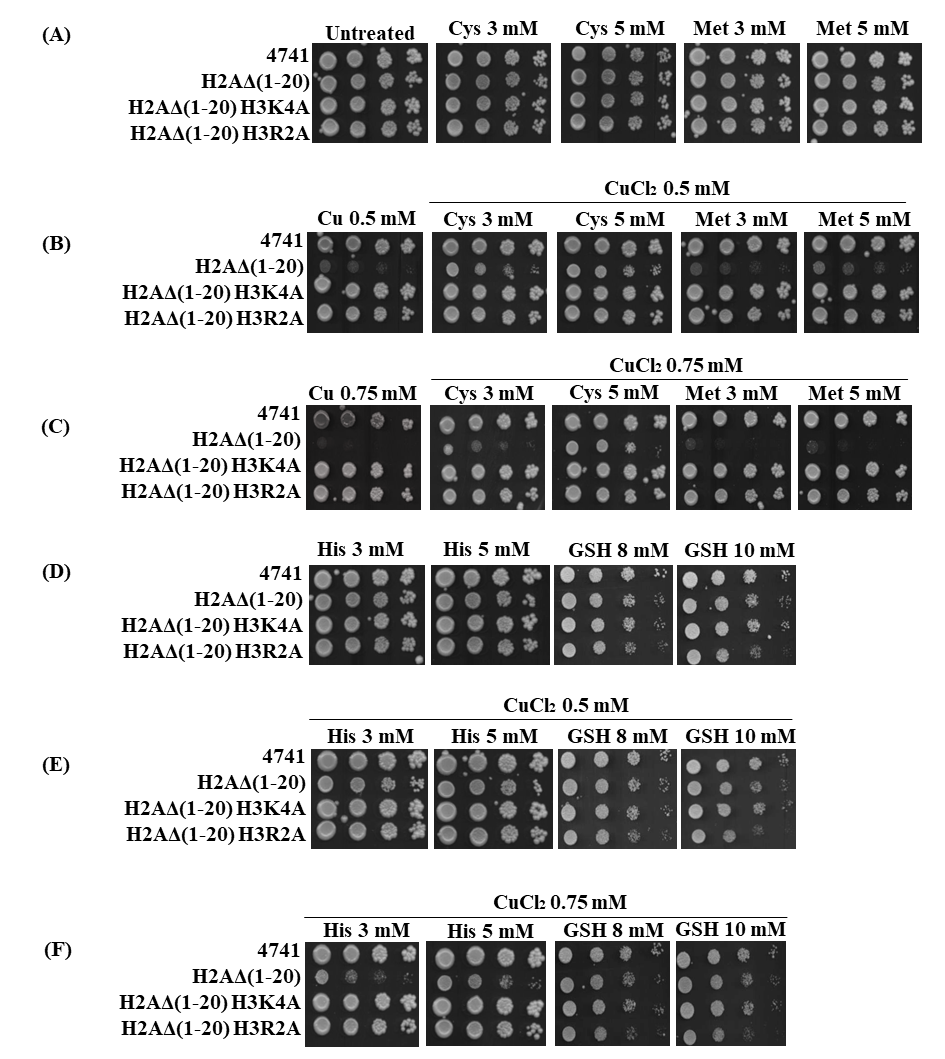


**(C)**

**(F)**

**(E)**

**(D)**

**(B)**

**(A)**

**Figure S12**

**Figure S12. The effect of copper on the growth of histone H2A mutants and wild type yeast cells is suppressed by supplementation of Cysteine, Histidine, Methionine and GSH.** (A-F) Spot test assay of histone H2A mutants along with wild-type cells to test their growth in solid SC growth medium. The cells were spotted from left to right, 10-fold serially diluted on the plates containing copper, cysteine, histidine, GSH, methionine and co-treatments as indicated above. UT means untreated, growth of cells in normal SC growth media.

.


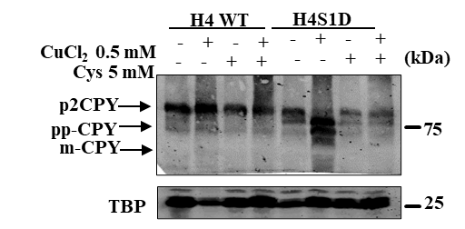

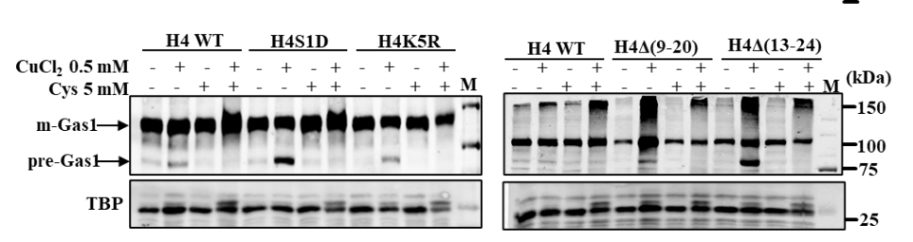


**(A)**

**(D)**

**Figure S13**

**(B)**


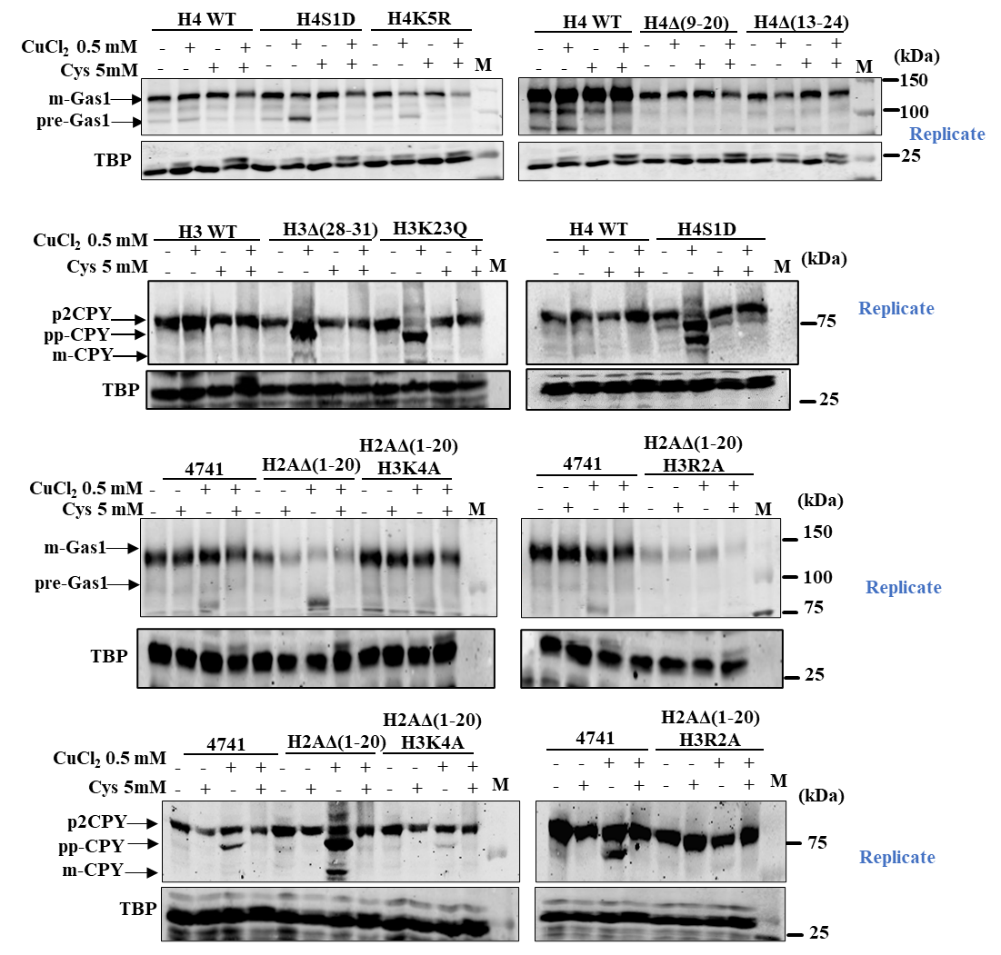

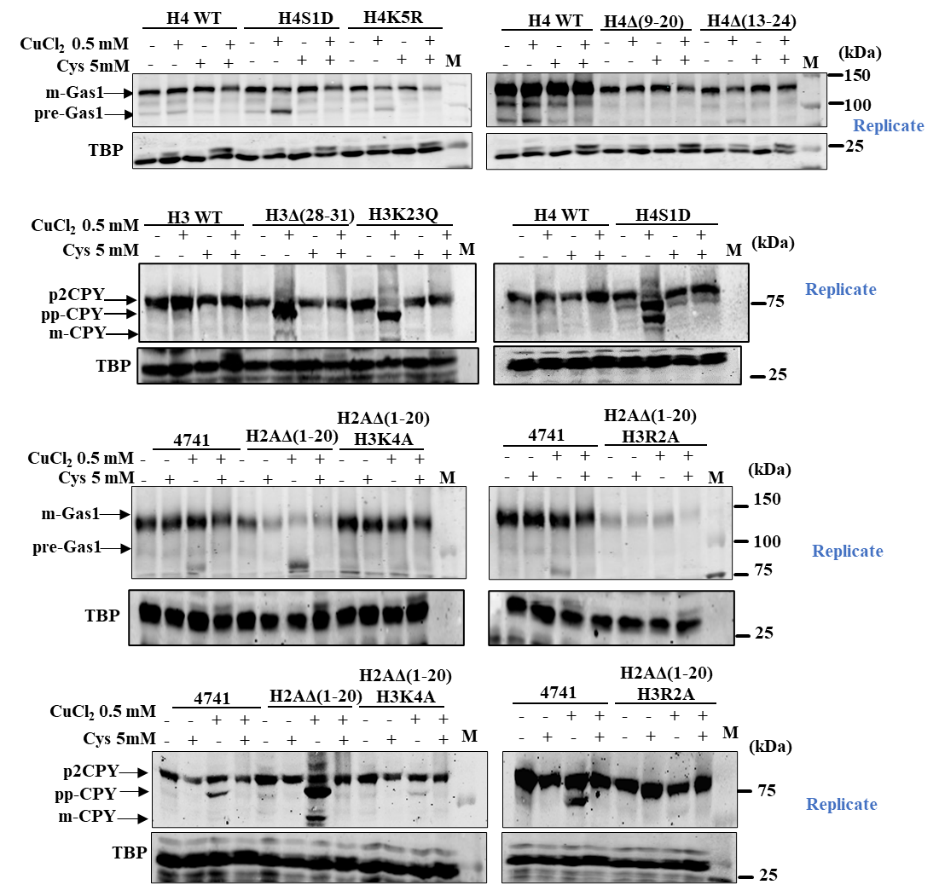


**(E)**


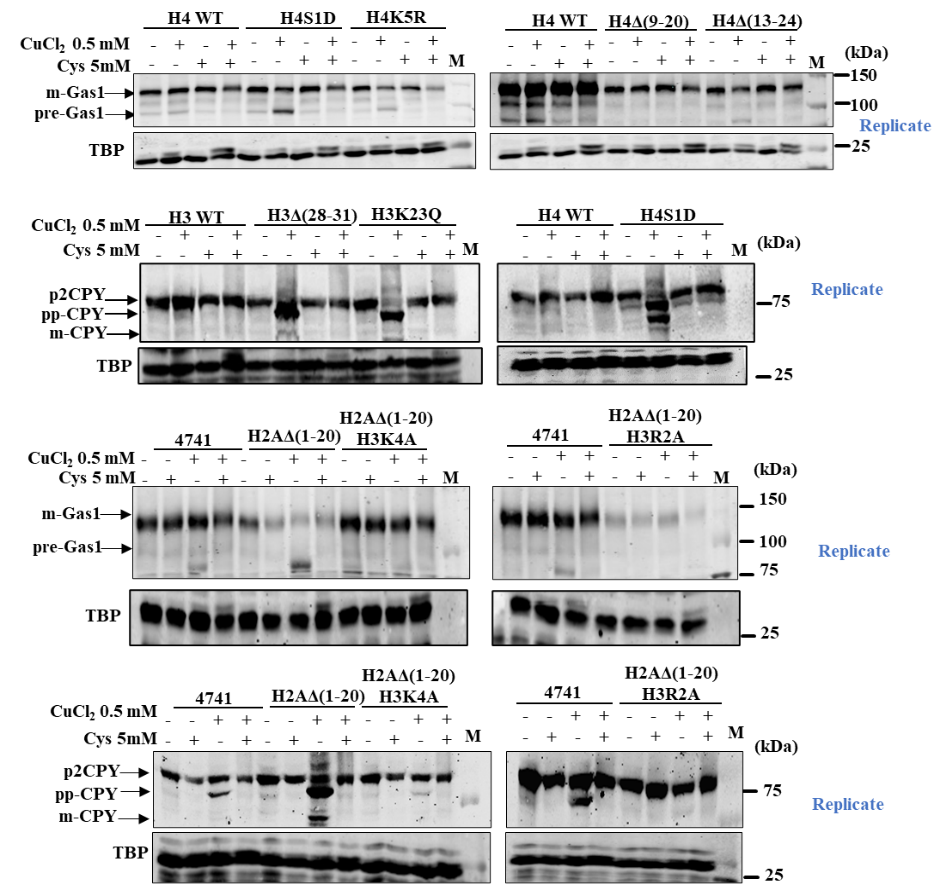


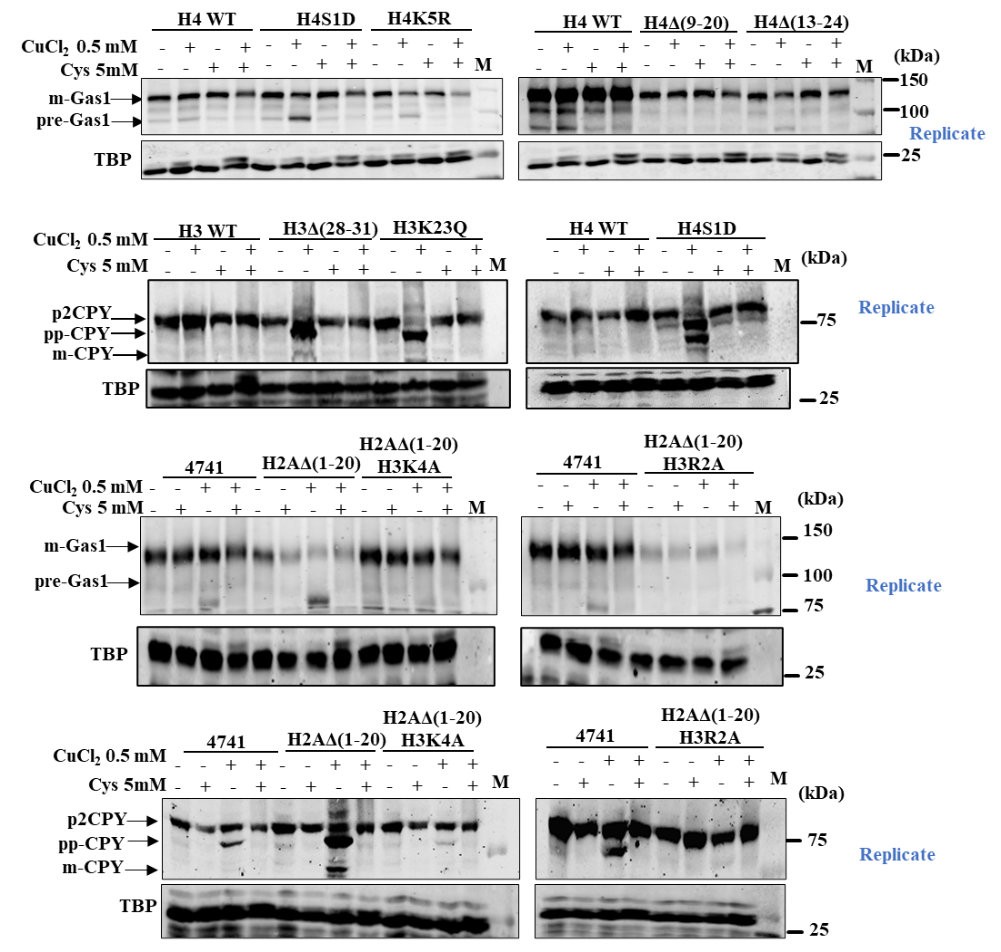
**
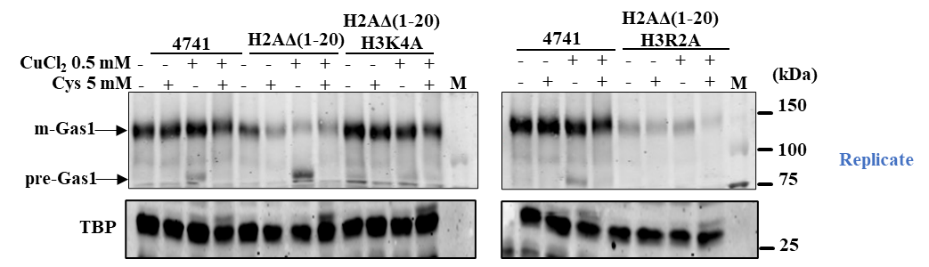
**

**(C)**

**(F)**


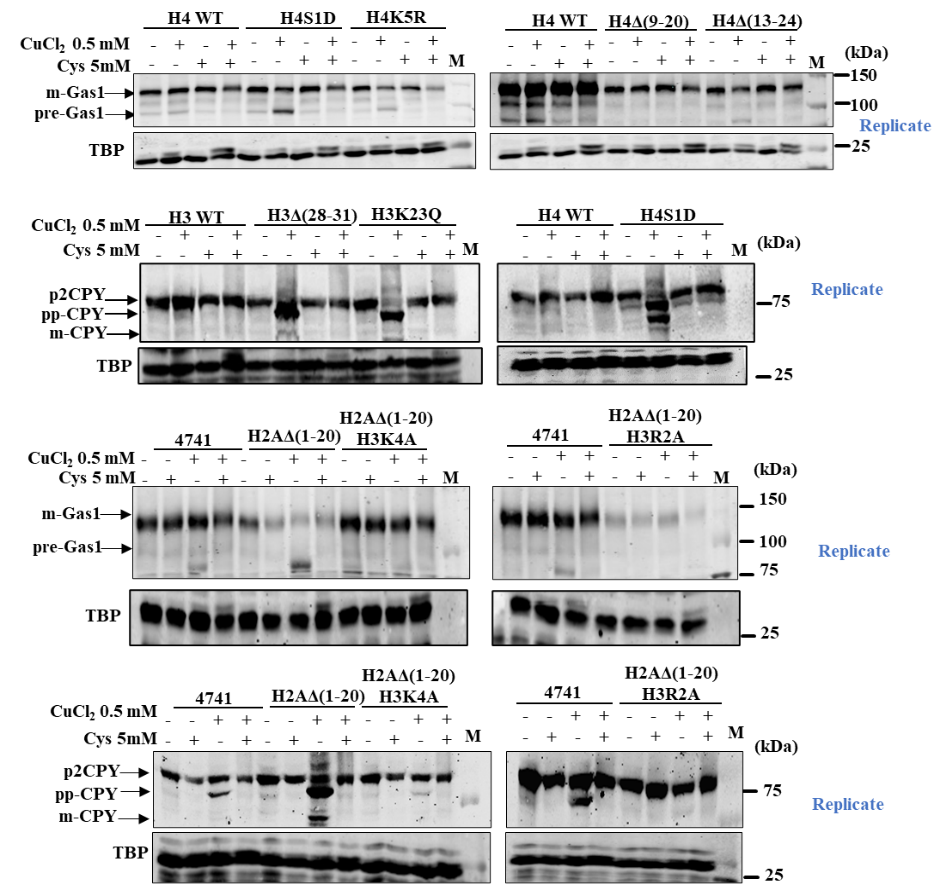


**
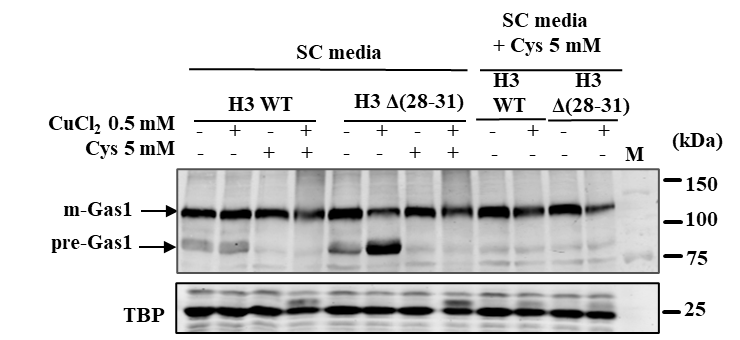
**

**(H)**

**(G)**

**
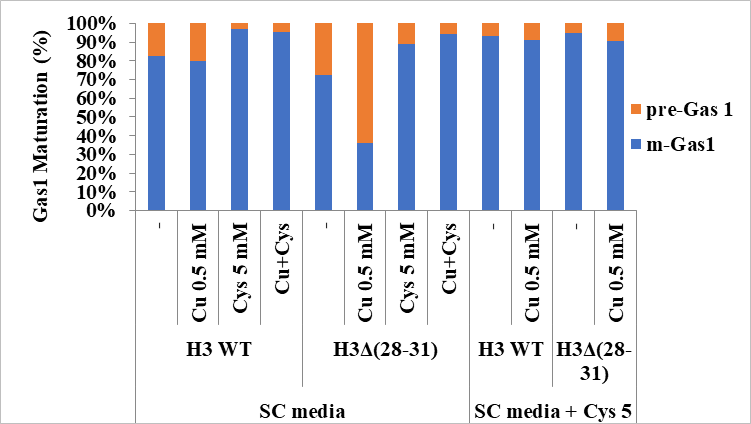
**

**Figure S13.** **Cysteine supplementation suppresses the effect of copper on protein translocation:** Replicates of western blots presented in Figure 6. (A-B) Immunoblot of Gas1 in copper sensitive histone H4 mutants and wildtype cells treated with CuCl_2_.2H_2_O (0.5 mM), cysteine (5 mM), and co-treatments. (C) Immunoblot of Gas1 in copper sensitive histone H2A mutants and wildtype cells treated with CuCl_2_.2H_2_O (0.5 mM), cysteine (5 mM), and co-treatments. (D-E) Immunoblot of pp-CPY in copper sensitive histone H3 and H4 mutants (H3Δ(28-31), H3K23Q, and H4S1D) and wildtype cells treated with CuCl_2_.2H_2_O (0.5 mM), cysteine (5 mM), and co-treatments. (F) Immunoblot of pp-CPY in copper-sensitive histone H2A mutants and wildtype cells treated with CuCl_2_.2H_2_O (0.5 mM), cysteine (5 mM), and co-treatments. (G) Immunoblot of Gas1 in a copper sensitive histone H3 mutant, H3Δ(28-31) and wildtype cells. Cells were first grown in presence of cysteine (5 mM) till mid-log phase, harvested, washed and resuspended in normal medium, grown for 2 hours in absence (-) or presence (+) of 0.5 mM of CuCl_2_.2H_2_O. TBP western blotting served as a protein loading control. (H) Quantification of above blot ‘G’, data indicates the ratio of precursor and mature forms of Gas1 in histone H3Δ(28-31) w.r.t. wild type cells.

**
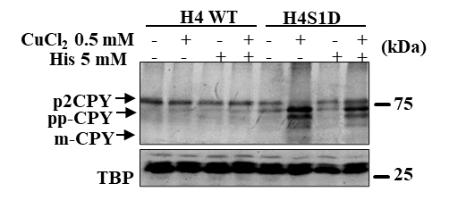
**

**(A)**

**Figure S14**


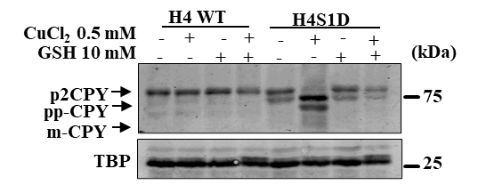


**(F)**

**
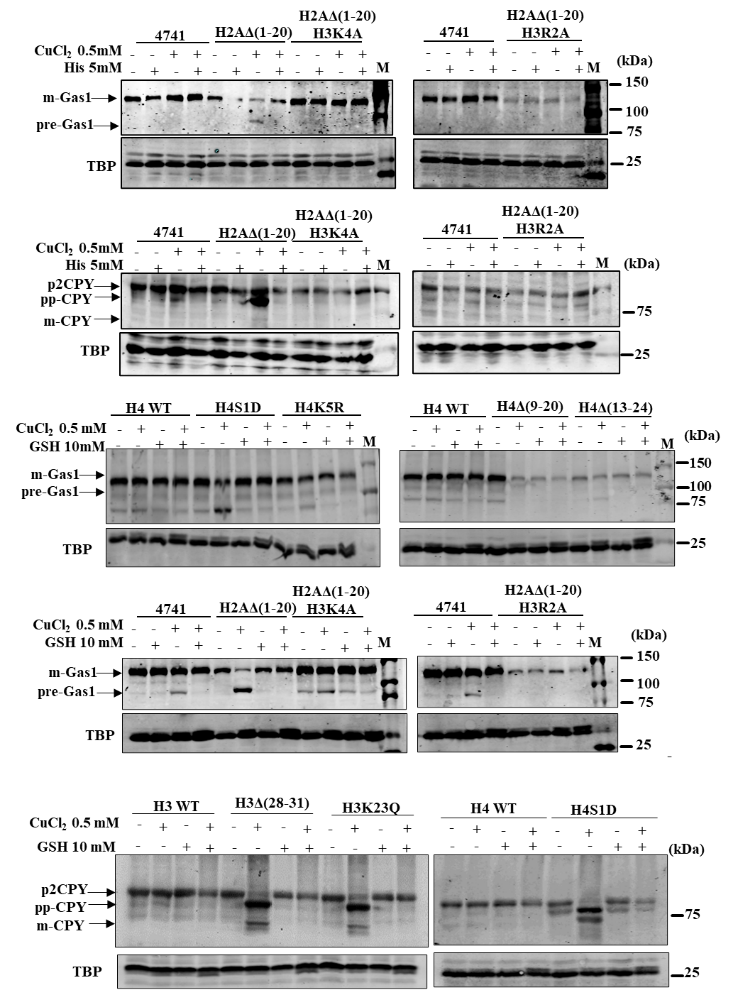
**

**(B)**

**(G)**

**
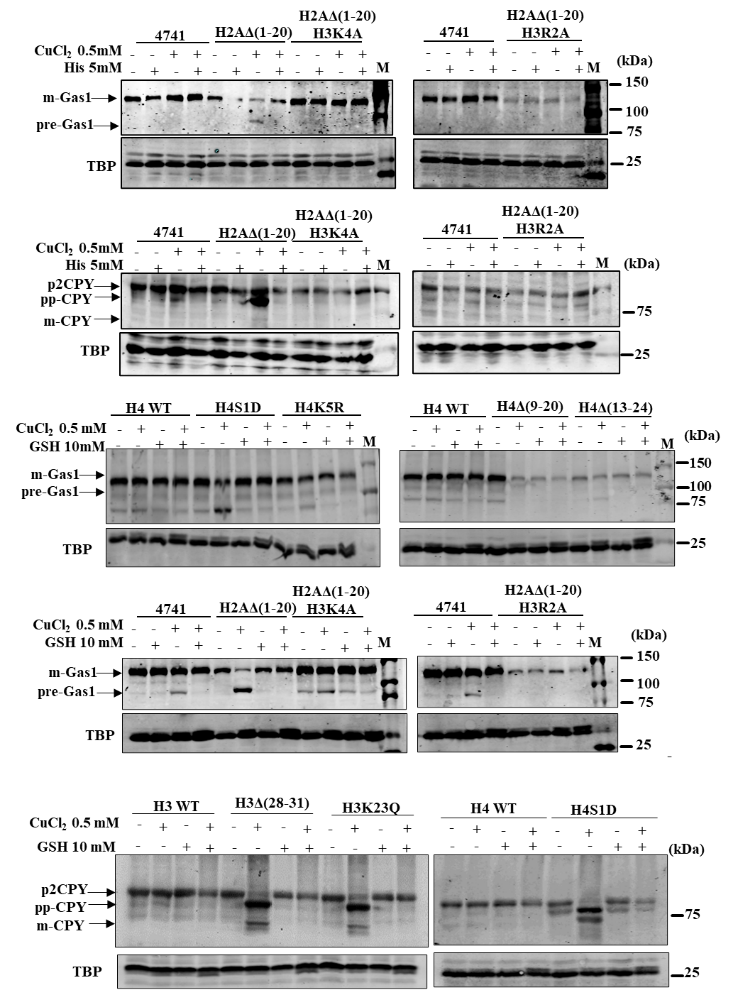
**

**
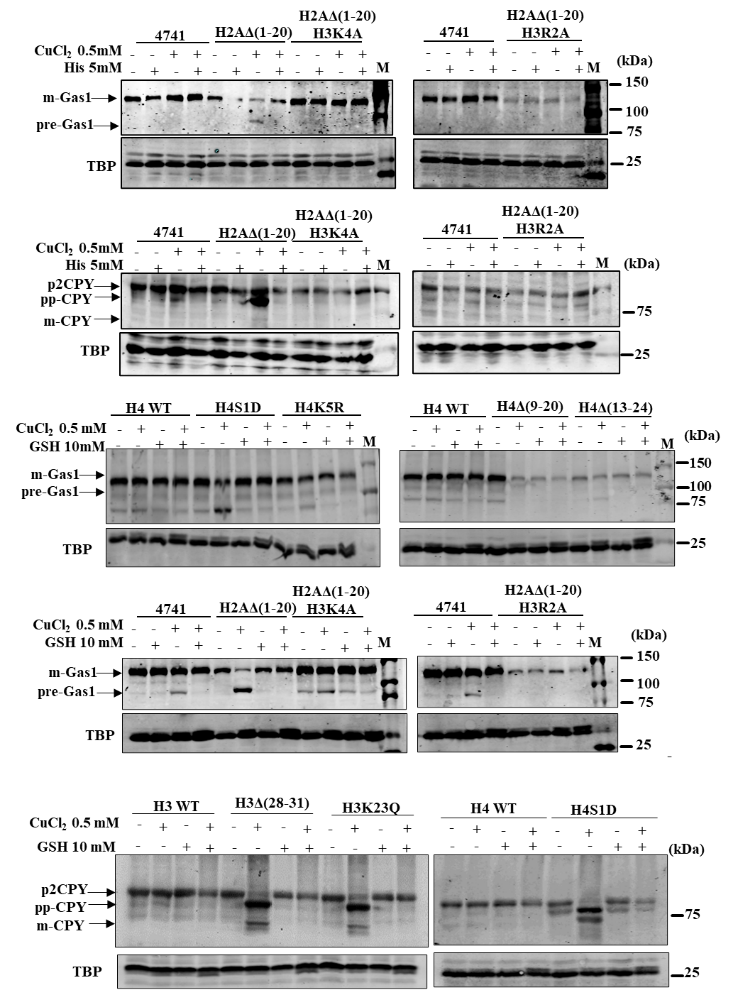
**

**(C)**


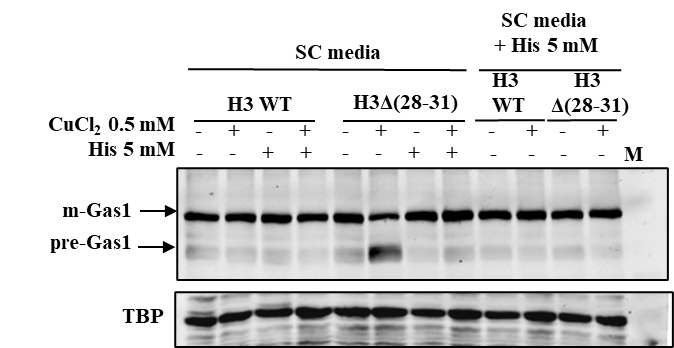


**(H)**

**(D)**


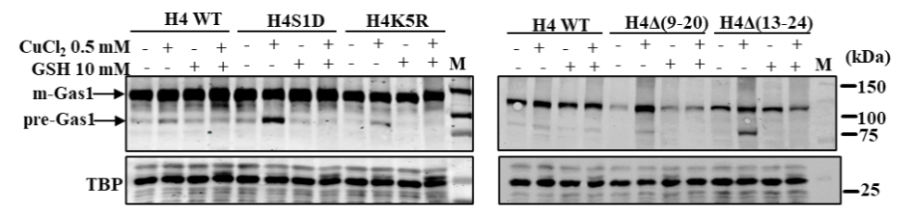


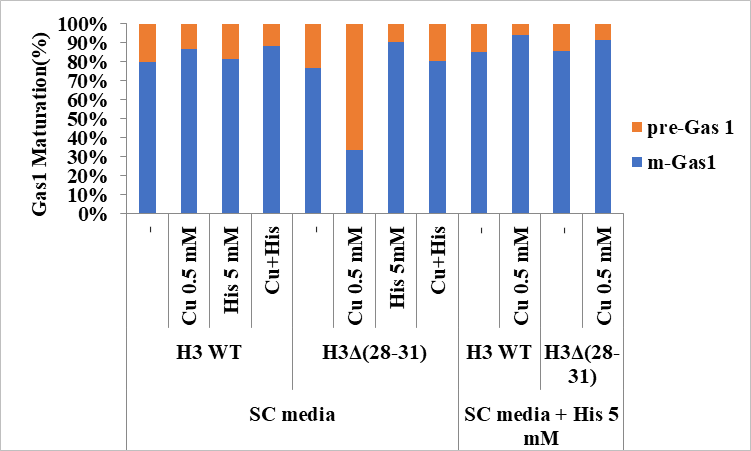


**(I)**

**(E)**

**
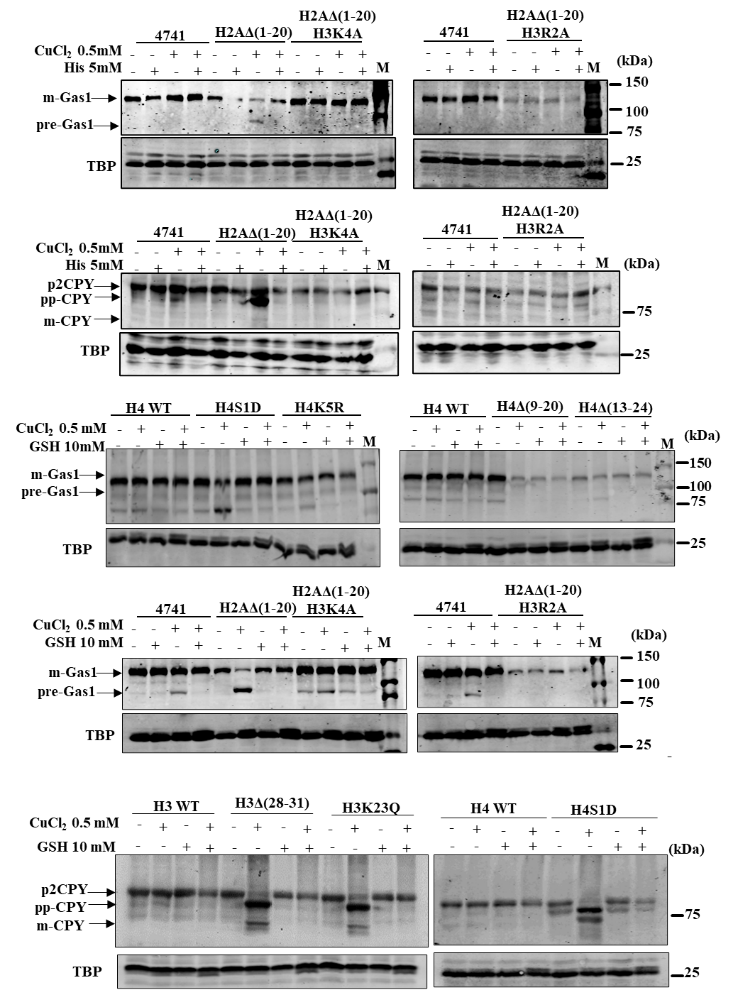
**

**
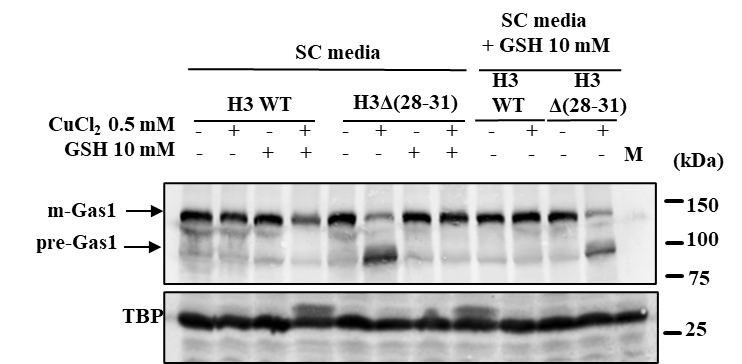
**
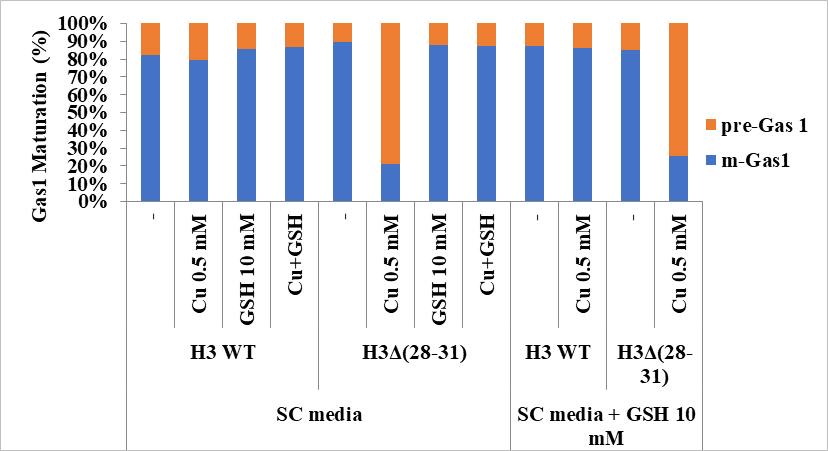


**(K)**

**(J)**

**Figure S14.** **Histidine and GSH supplementation suppresses the effect of copper on protein translocation process:** Replicates of western blots presented in Figure 7 and 8. (A-C) Immunoblot of Gas1 and CPY in copper sensitive histone H4 and H2A mutants along with wildtype cells treated with CuCl_2_.2H_2_O (0.5 mM), histidine (5 mM), and co-treatments. (D-G) Immunoblot of Gas1 and CPY in copper sensitive histone H3 and H4 mutants along with wildtype cells treated with CuCl_2_.2H_2_O (0.5 mM), GSH (10 mM), and co-treatments. (H) Immunoblot of Gas1 in copper sensitive histone H3Δ(28-31) and wildtype cells treated with CuCl_2_.2H_2_O (0.5 mM) for 2 hours. Cells were first grown in SC medium in presence of histidine (5 mM) till mid-log phase, harvested, washed and resuspended in normal SC medium, grown for 2 hours in absence and presence of 0.5 mM of CuCl_2_.2H_2_O. (I) Quantification of above immunoblot ‘H’, data indicates the ratio of precursor and mature forms of Gas1 in histone H3Δ(28-31) w.r.t. wild type cells. (J) Immunoblot of Gas1 in copper sensitive histone H3Δ(28-31) and wildtype cells treated with CuCl_2_.2H_2_O (0.5 mM) for 2 hours. Cells were first grown in SC medium in presence of GSH (10 mM) till mid-log phase, harvested, washed and resuspended in normal SC medium, grown for 2 hours in absence (-) or presence (+) of 0.5 mM of CuCl_2_.2H_2_O. TBP western blotting served as a protein loading control. (K) Quantification of above immunoblot ‘J’, data indicates the ratio of precursor and mature form of Gas1 in histone H3Δ(28-31) w.r.t. wild type.

**
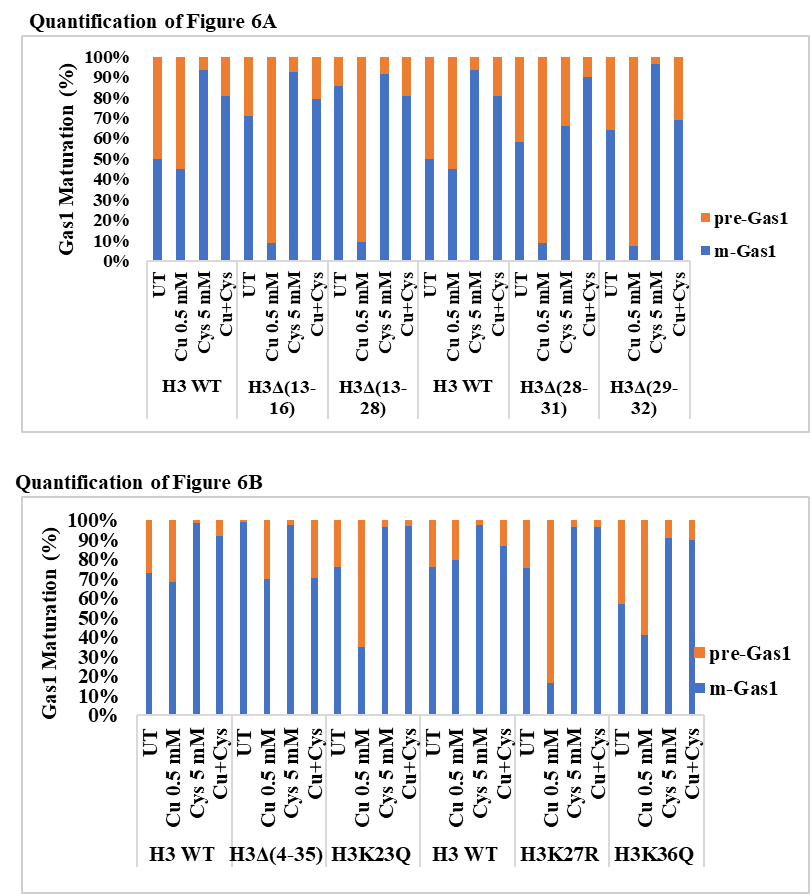
**

**Figure S15**

**(B)**

**(A)**

**Figure S15.** **Quantification of Gas1 in copper-sensitive histone mutants.** (A-B) Quantification of the western blots presented in Figure 6. Data indicates the ratio of precursor and mature form of Gas1 in histone H3 mutants w.r.t. wild type cells.


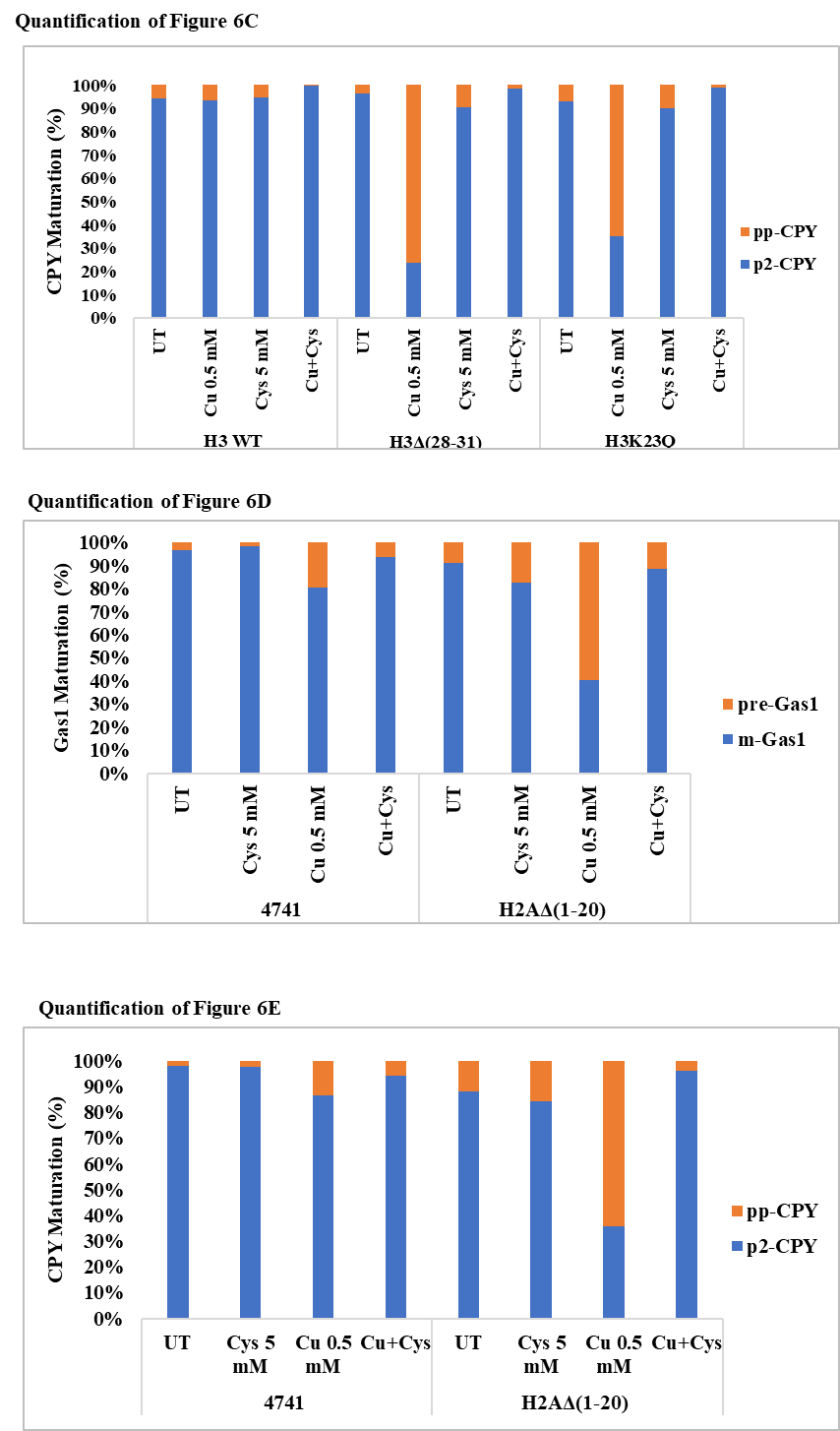


**(B)**

**(C)**

**(A)**

**Figure S16**

**Figure S16.** **Quantification of Gas1 and CPY** **in copper-sensitive histone mutants.** (A-C) Quantification of the western blots presented in Figure 6. Data indicates the ratio of precursor and mature forms of Gas1 and the ratio of p2 and pp forms of CPY in histone H3, and H2A mutants w.r.t. wild type cells.


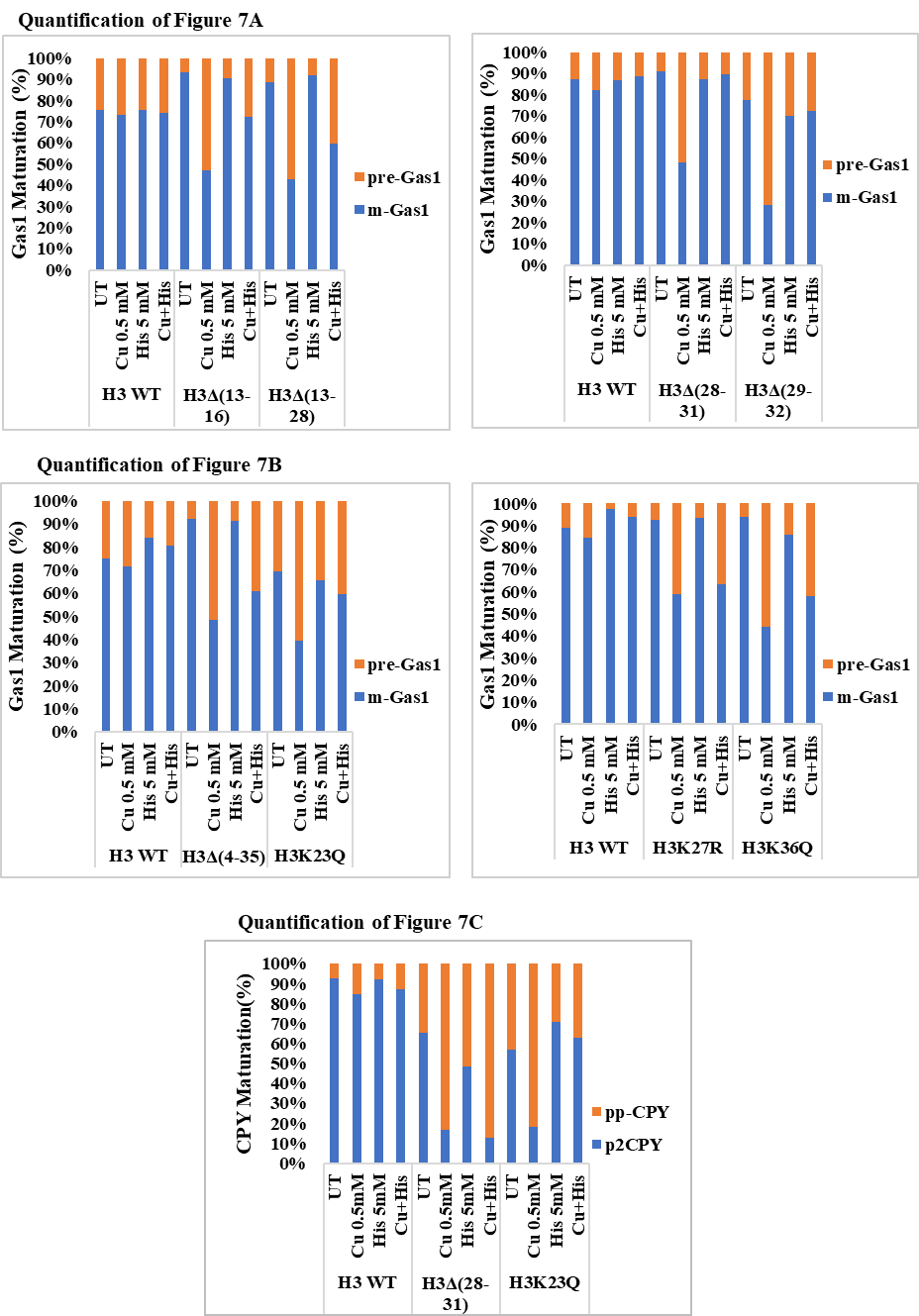


**(C)**

**Figure S17**

**(A)**

**(B)**

**Figure S17. Quantification of Gas1 and CPY in copper-sensitive histone mutants.** (A-C) Quantification of the western blots presented in Figure 7. Data indicates the ratio of precursor and mature forms of Gas1 and p2 and pp forms of CPY in copper-sensitive histone H3, mutants w.r.t. wild type.

**Figure S18**


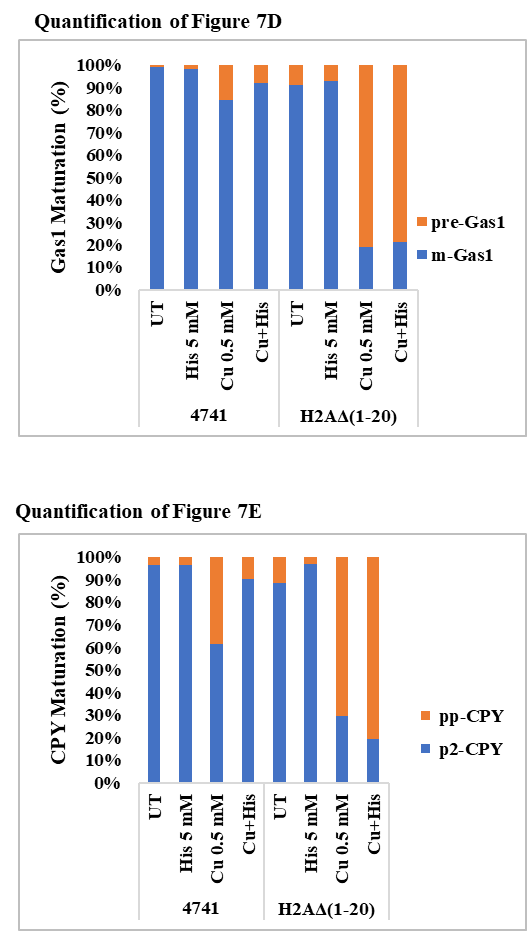


**(B)**

**(A)**

**Figure S18.** **Quantification of Gas1 and CPY** **in histone H2A mutants.** (A-B) Quantification of the western blots presented in Figure 7. Data indicates the ratio of precursor and mature forms of Gas1 and p2 and pp forms of CPY in histone H2A mutants w.r.t. wild type cells.

**
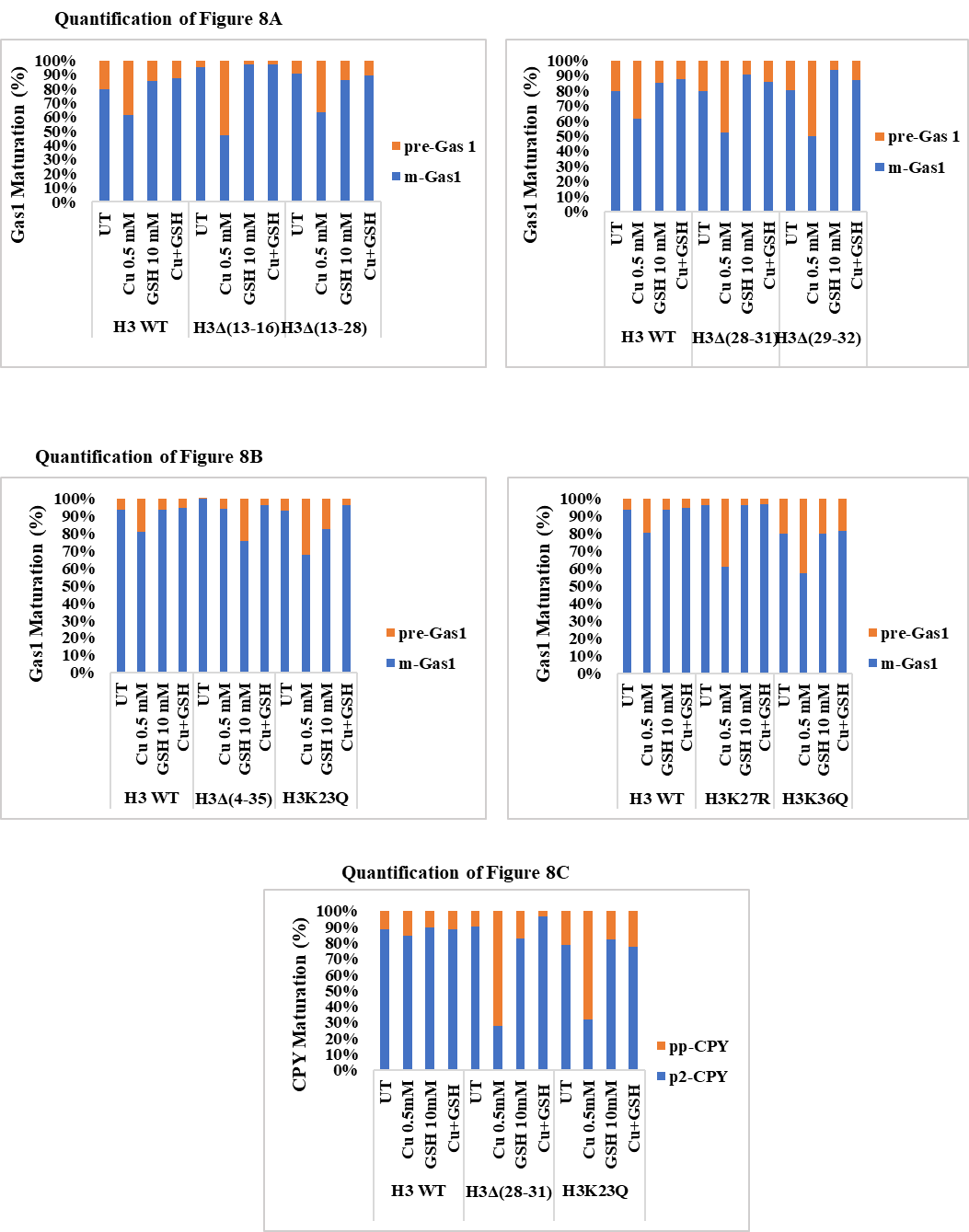
**

**(B)**

**(C)**

**(A)**

**Figure S19**

**Figure S19.** **Quantification of Gas1 and CPY in copper-sensitive histone mutants.** (A-C) Quantification of the western blots presented in Figure 8. Data indicates the ratio of precursor and mature forms of Gas1 and the ratio of p2 and pp forms of CPY in histone H3 mutants w.r.t. wild type.

**Figure S20**


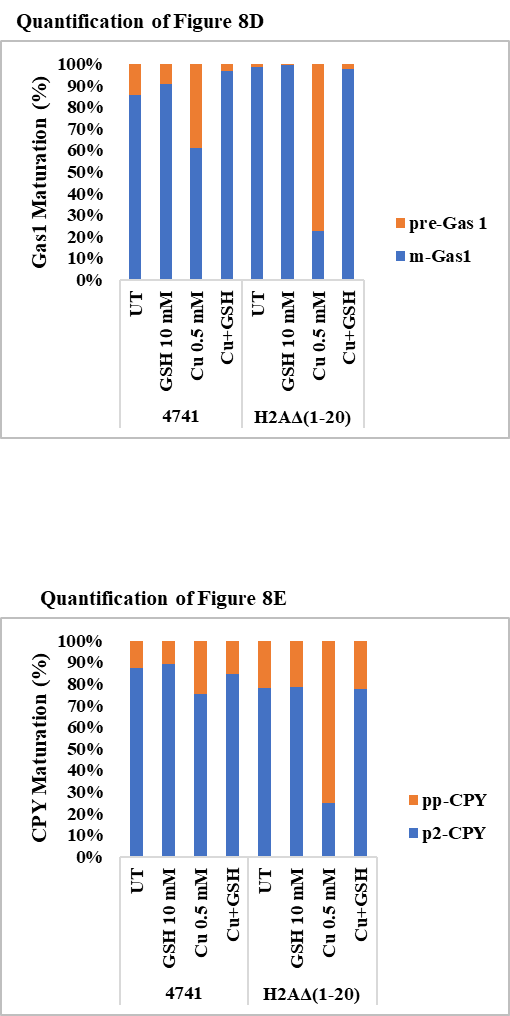


**(A)**

**(B)**

**Figure S20.** **Quantification of Gas1 and CPY in copper-sensitive histone mutants.** (A-B) Quantification of the western blots presented in Figure 8. Data indicates the ratio of precursor and mature forms of Gas1 and the ratio of p2 and pp forms of CPY in histone H2A mutants w.r.t. wild type.

**
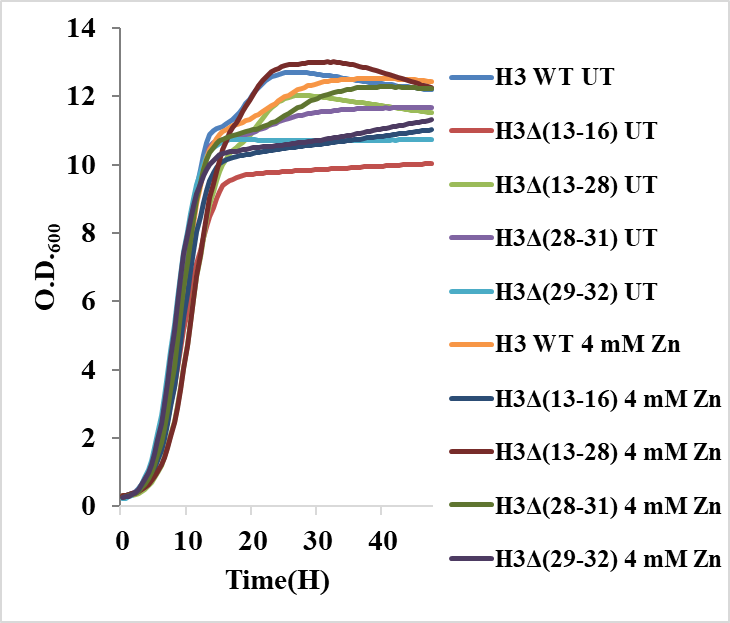

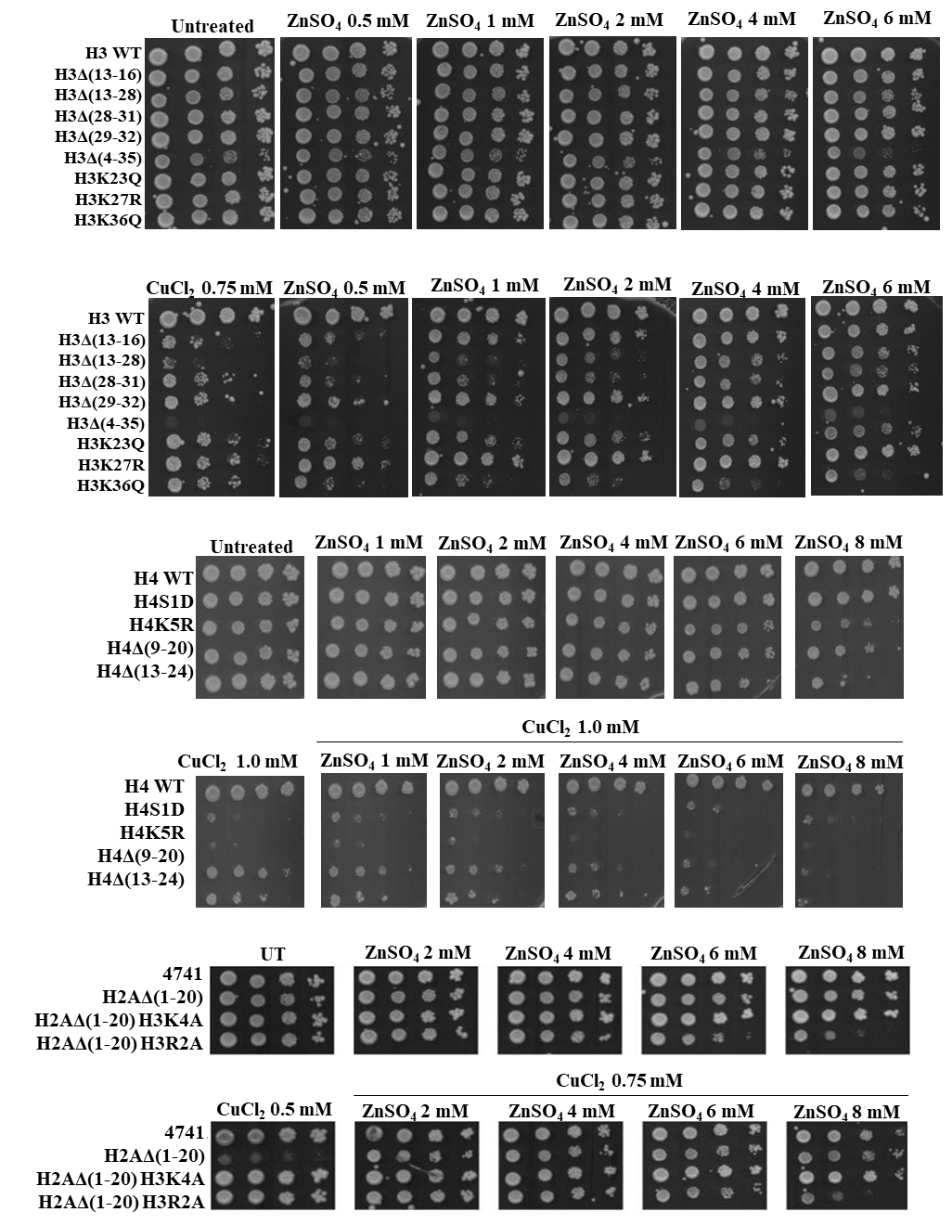
**

**(F)**

**(A)**

**Figure S21**

**
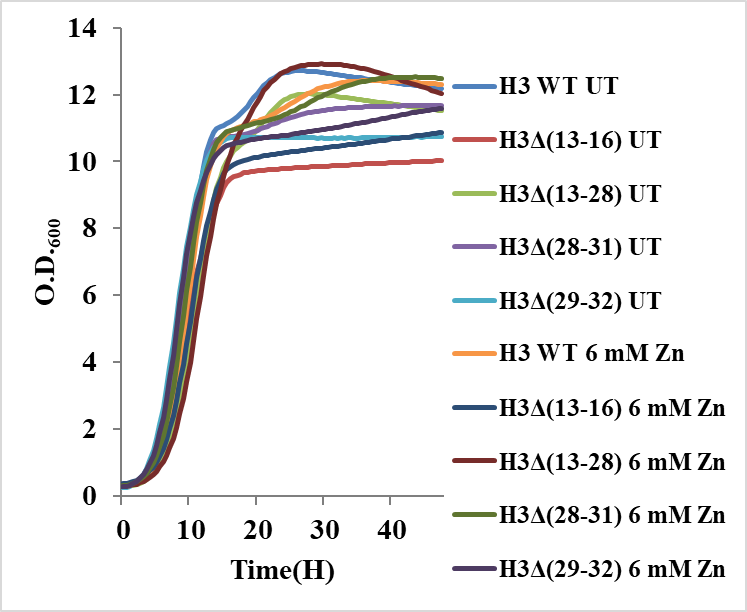
**

**(G)**

**
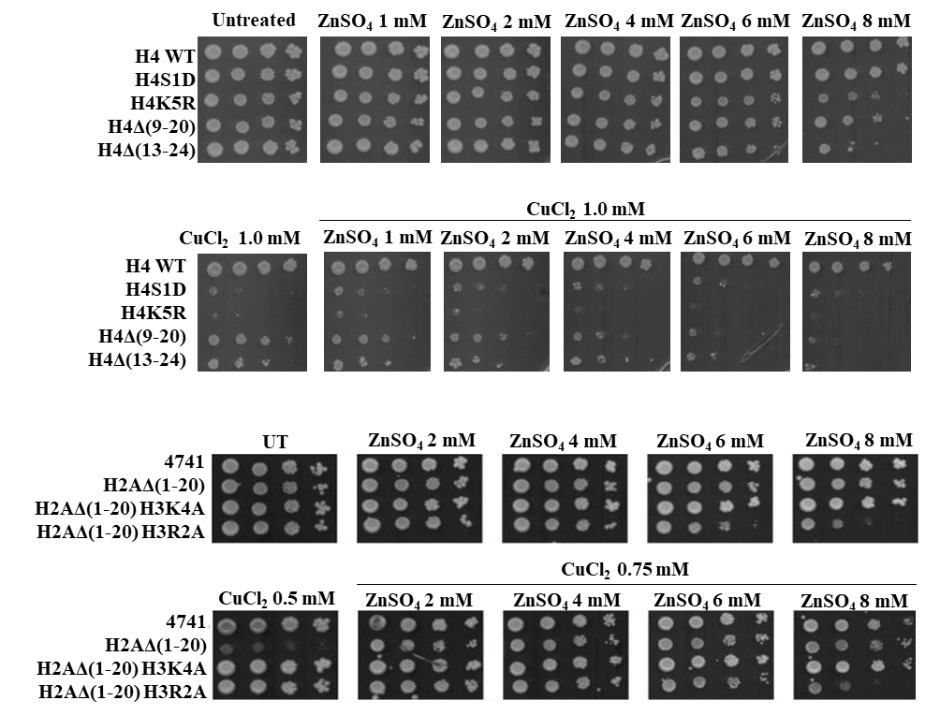
**

**(B)**

**
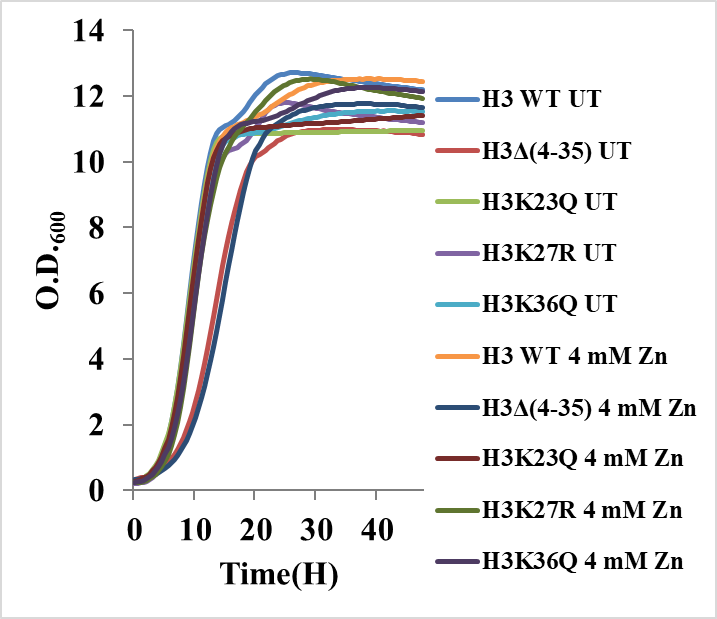
**

**(H)**

**(C)**

**
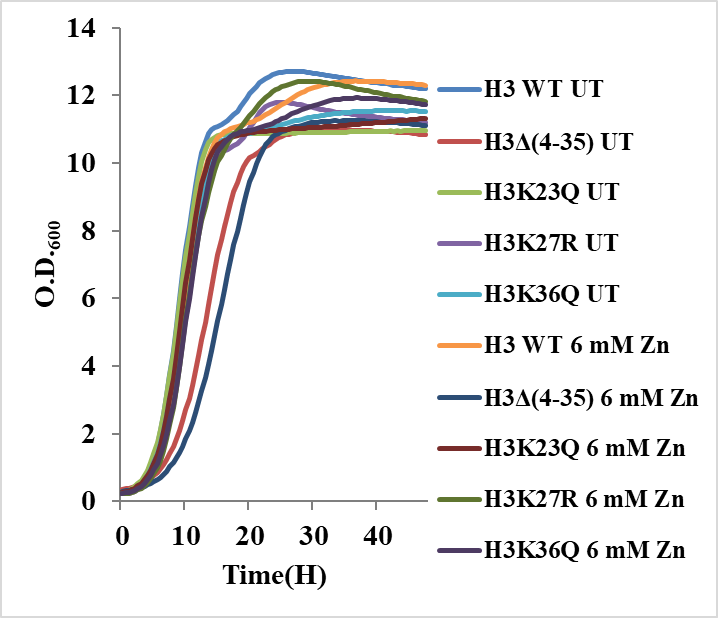
**

**(E)**

**(D)**

**(I)**

**
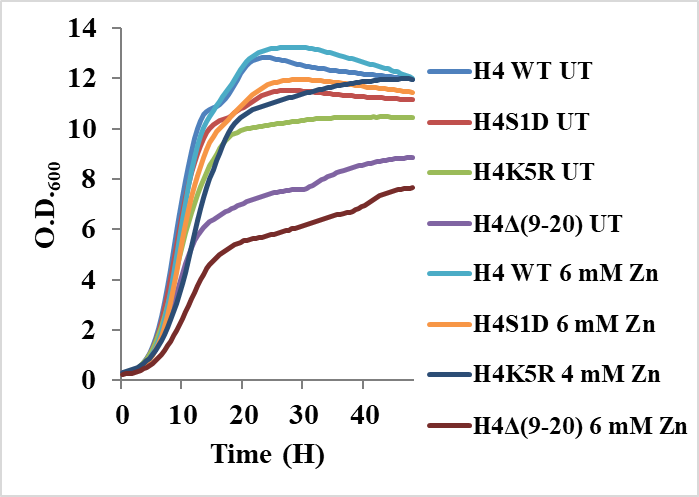

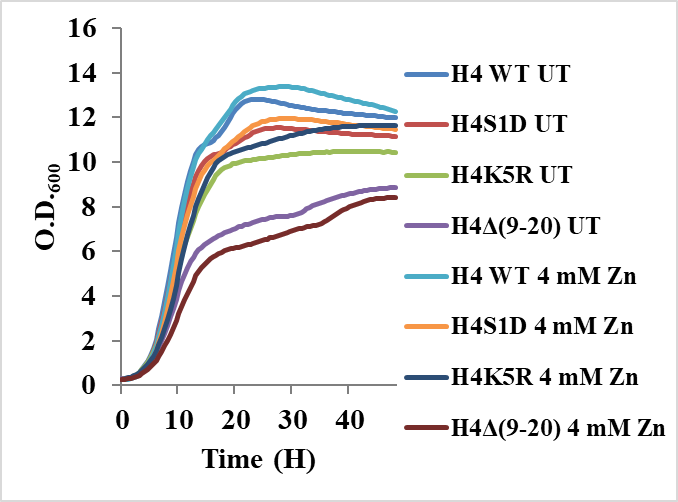
**

**Figure S21. Effect of copper and zinc on the growth of histone mutants.** (A-C) Spot assay of copper-sensitive histone H3, H4, and H2A mutants, respectively, along with wild-type to test their growth. The 10-fold serially diluted cultures of cells were spotted from left to right on SC+agar solid medium in absence (untreated) or presence of different concentrations of copper, zinc, and co-treatment as shown above. UT means untreated, growth of cells in normal media. (D-I) Growth curve analysis of copper sensitive histone H3 and H4 mutants in 4 mM and 6 mM zinc.

**(A)**

**Figure S22**

**
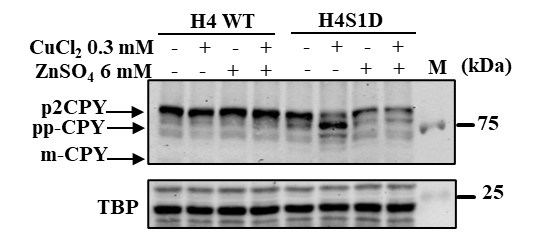
**

**
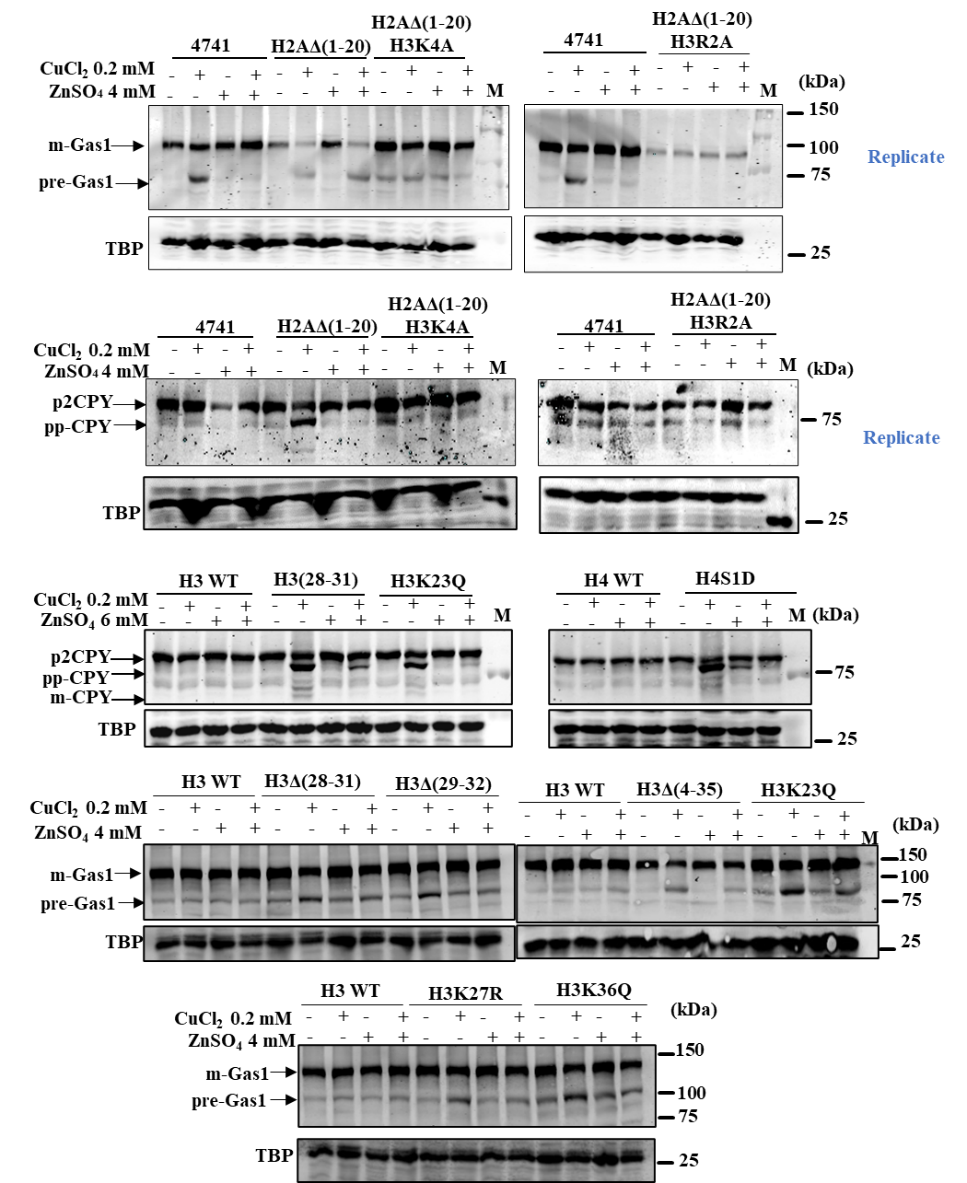
**

**(B)**

**(C)**

**(D)**

**(E)**

**(F)**

**Figure S22. Suppression of copper-induced protein translocation defect by zinc:** (A) Immunoblot of CPY in copper-sensitive histone H4S1D mutant and wildtype cells treated with CuCl_2_.2H_2_O (0.3 mM), ZnSO_4_.7H_2_O (6 mM), and co-treatment. (B) Immunoblot of Gas1 in copper sensitive histone H2A mutants along with wildtype cell treated with CuCl_2_.2H_2_O (0.2 mM), ZnSO_4_.7H_2_O (4 mM), and co-treatment. (C) Immunoblot of CPY in copper-sensitive histone H2A mutants and wildtype cells treated with CuCl_2_.2H_2_O (0.2 mM), ZnSO_4_.7H_2_O (4 mM), and co-treatment. (D) Immunoblot of CPY in copper-sensitive histone H3 and H4 mutants and wildtype cells treated with CuCl_2_.2H_2_O (0.2 mM), ZnSO_4_.7H_2_O (6 mM), and co-treatment. (E-F) Immunoblot of Gas1 in copper-sensitive histone H3 mutants and wildtype cells treated for 2 hours with CuCl_2_.2H_2_O (0.2 mM), ZnSO_4_.7H_2_O (4 mM), and co-treatment. TBP western blotting served as a protein loading control.

**
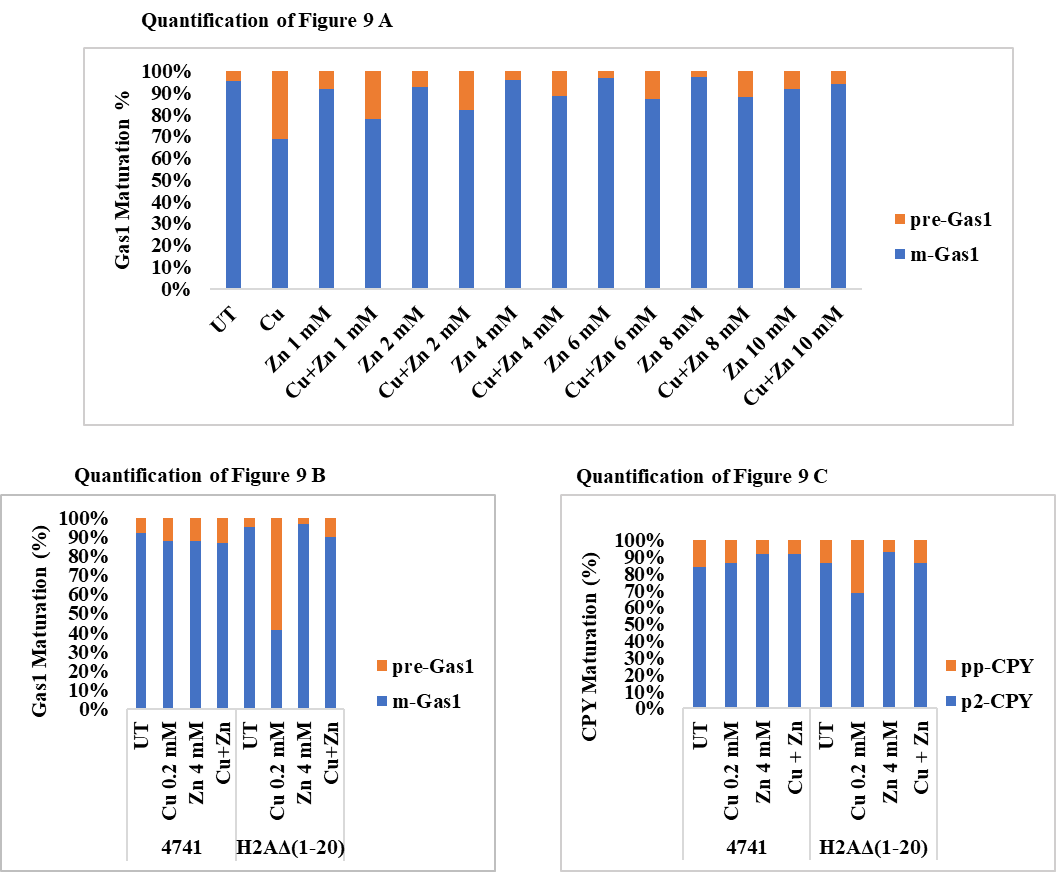
**

**(A)**

**Figure S23**

**(B)**

**(C)**

**Figure S23.** **Quantification of Gas1 and CPY secretary proteins in copper sensitive H2A histone mutants.** (A-C) Quantification of the western blots presented in Figure 9. Data indicates the ratio of precursor and mature forms of Gas1 and the ratio of p2 and pp forms of CPY in H2A mutants and wild type yeast cells.

**Figure S24**

**
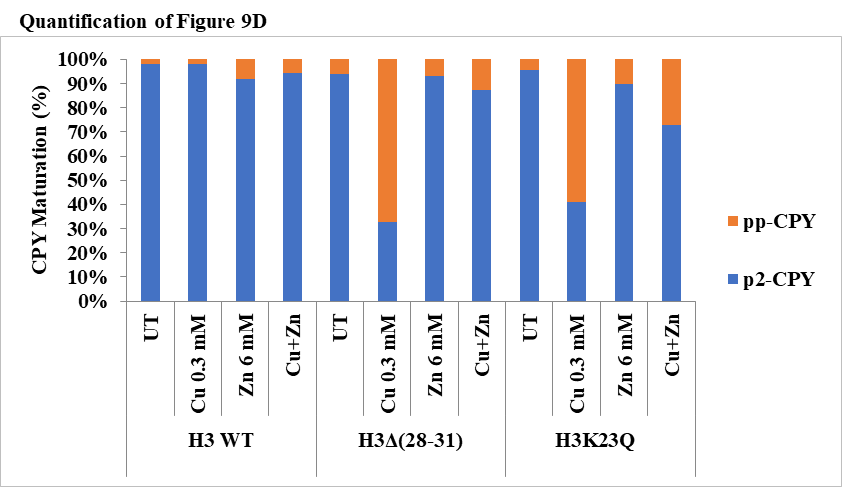
**

**(A)**

**Figure S24.** **Quantification of CPY in copper sensitive histone mutants.** (A) Quantification of the western blot presented in Figure 9. Data indicates the ratio of p2 and pp forms of CPY in histone H3 mutants as compared to wild type cells.


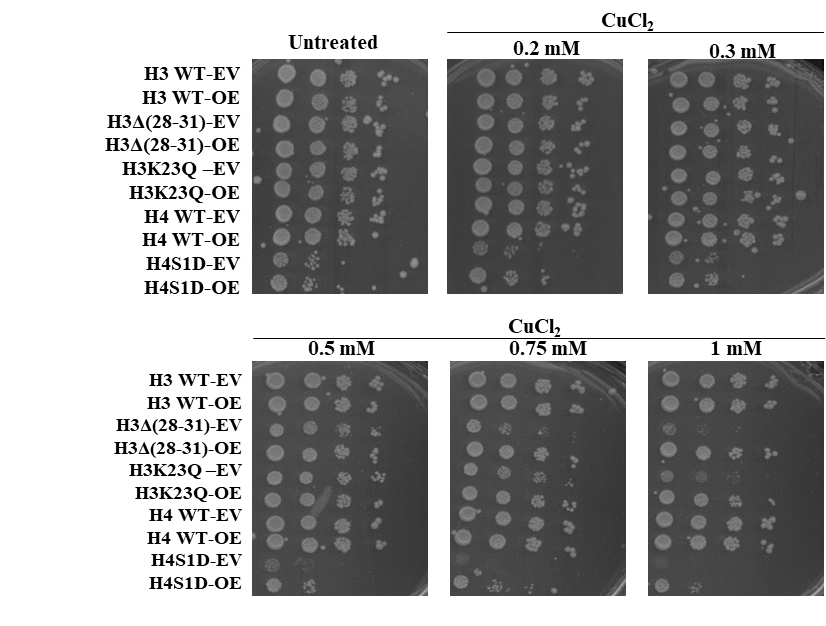


**Figure S25**

**
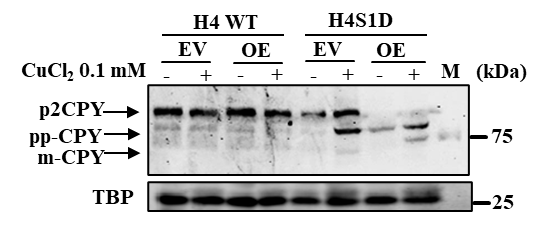
**

**(D)**

**(A)**

**(E)**

**
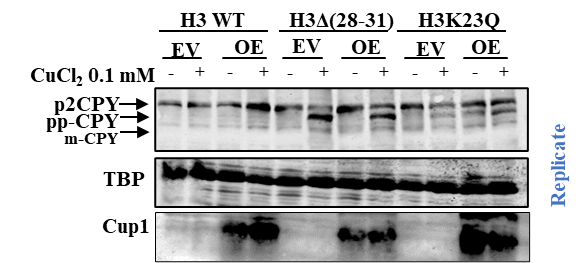
**

**(B)**


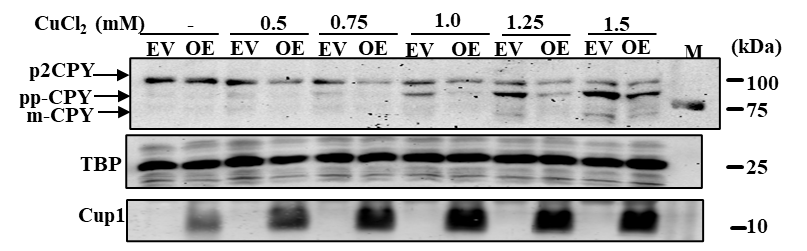

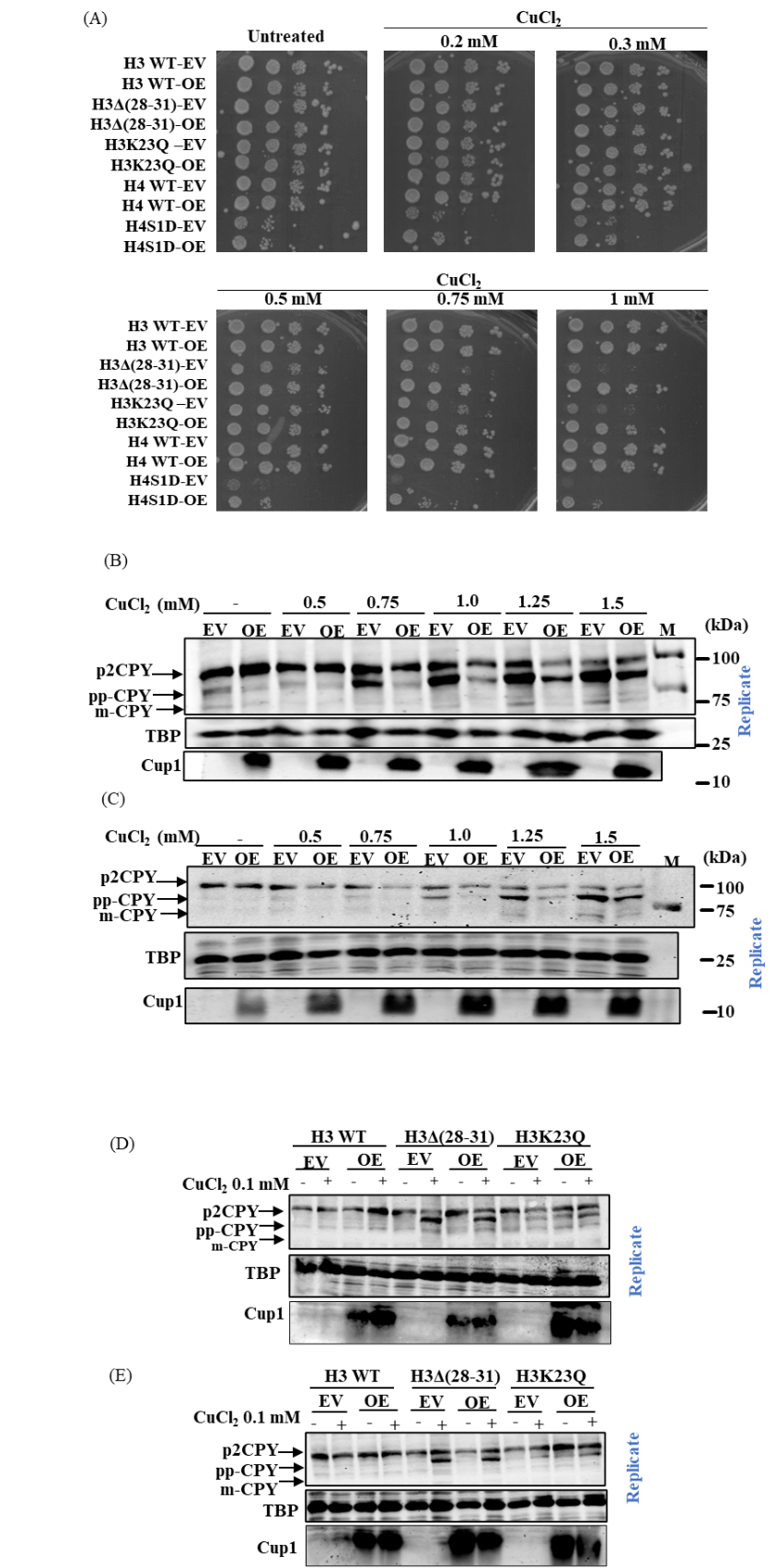


**
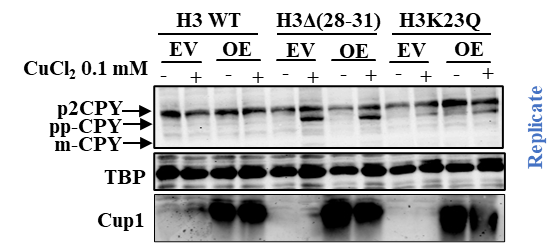
**

**(F)**


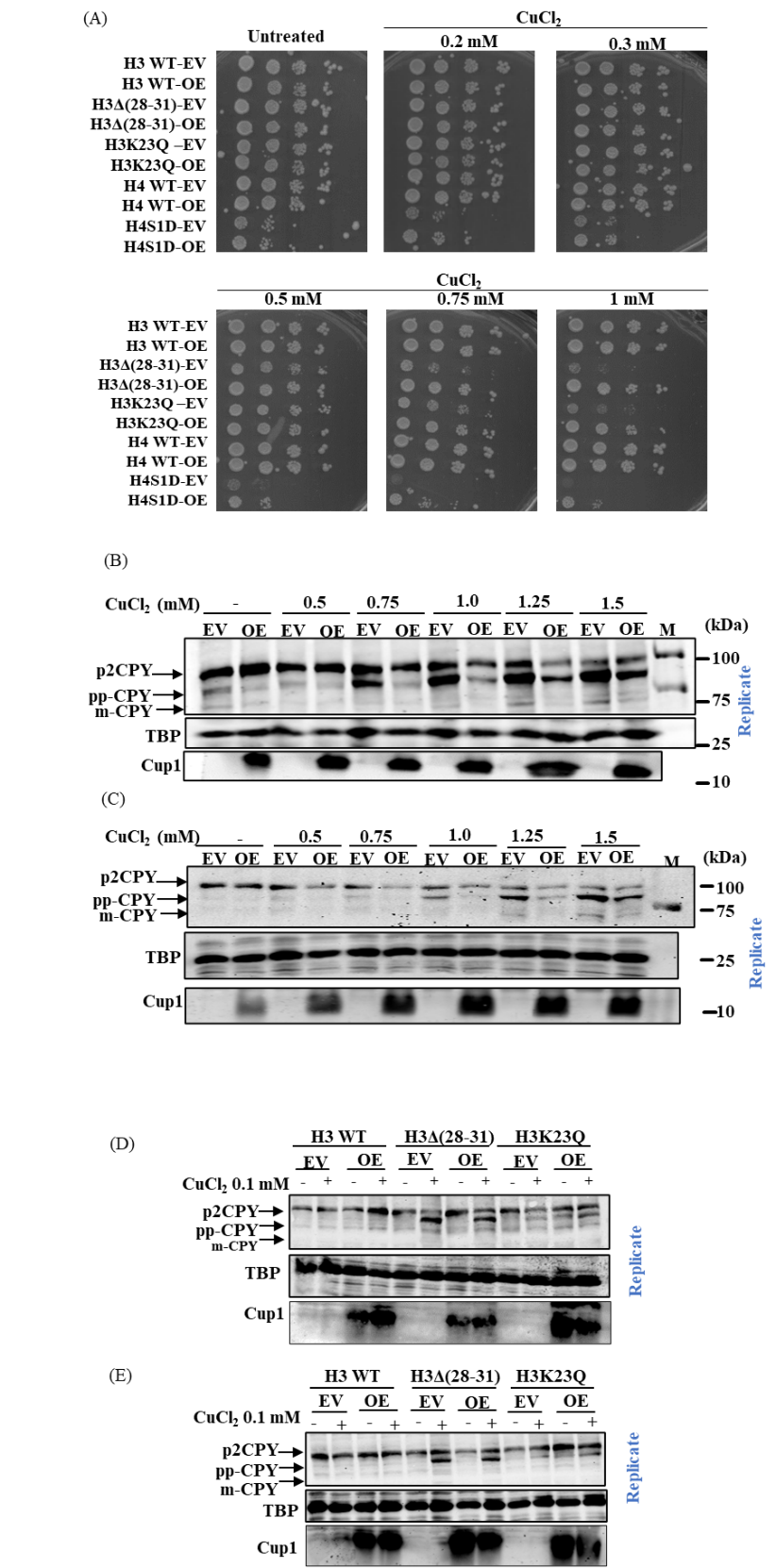


**(C)**

**Figure S25. Cup1 overexpression suppresses the copper induced protein translocation defects:** (A) Spot test assay of copper sensitive histone H3, and H4 mutants along with wild type cells to test their growth on SC+Agar solid medium in absence (untreated) and presence (+) of different concentrations of copper as indicated. The 10-fold serially diluted cells were spotted from left to right. UT means untreated growth of cells in normal SC media. (B-C) Immunoblot analysis of CPY in EV/OE transformed wild type cells treated with CuCl_2_.2H_2_O (0.5 mM, 0.75 mM, 1.0 mM, 1.25 mM, and 1.5 mM) for 2 hours. (D-F) Immunoblot analysis of CPY in EV/OE transformed histone H3 and H4 mutants and wild type cells treated with CuCl_2_.2H_2_O (0.1 mM) for 2 hours. TBP western blotting served as protein loading control. EV, Empty Vector; OE, Cup1 Overexpression.

**Figure S26**


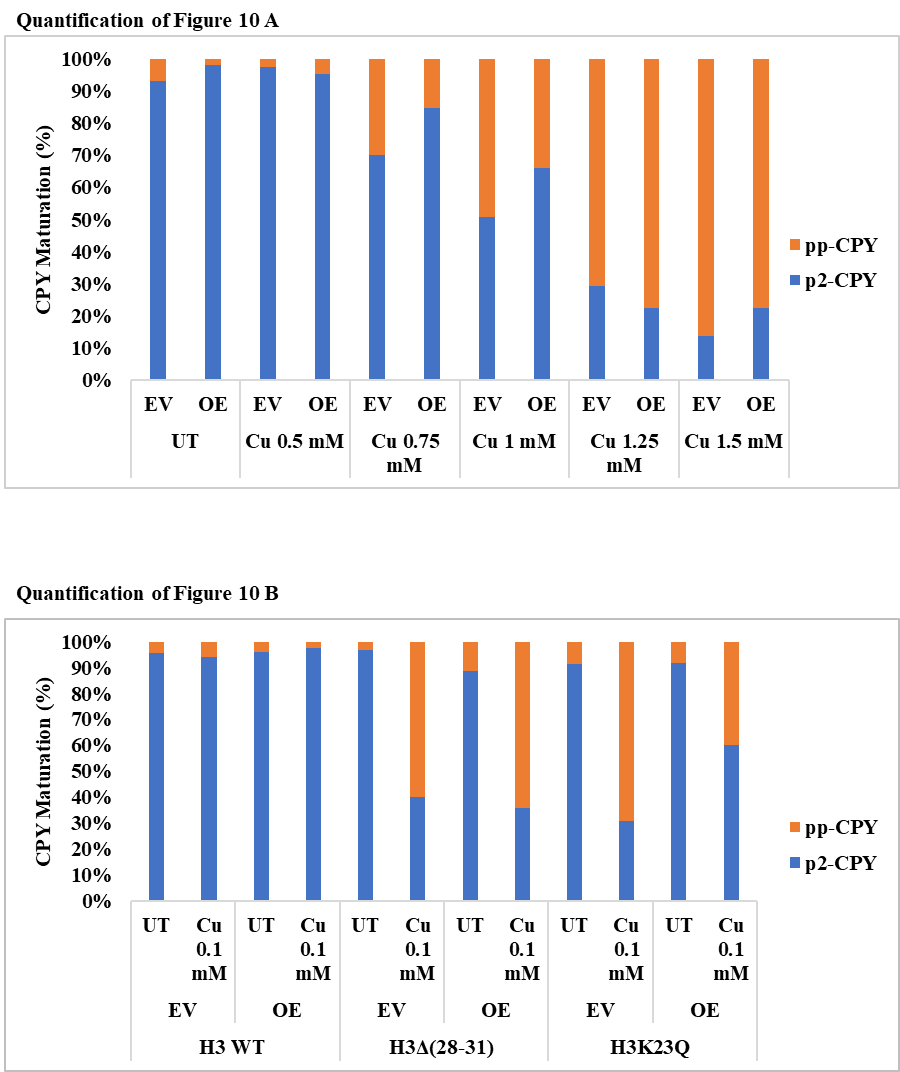


**(B)**

**(A)**

**Figure S26.** **Quantification of CPY in copper sensitive histone mutants.** (A-B) Quantification of the western blots presented in Figure 10. Data indicates the ratio of p2 and pp forms of CPY in histone H3 mutants, and wild type cells.


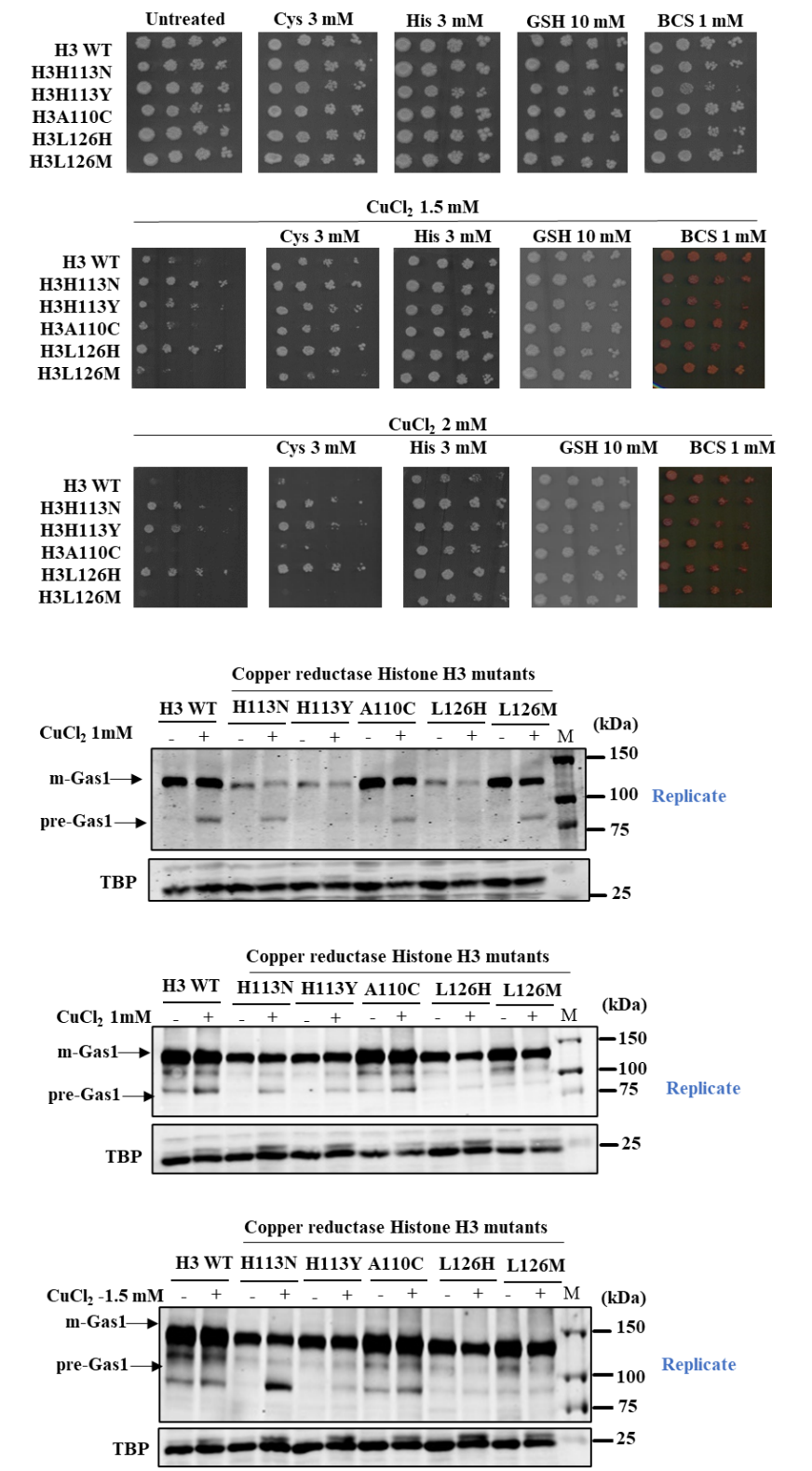

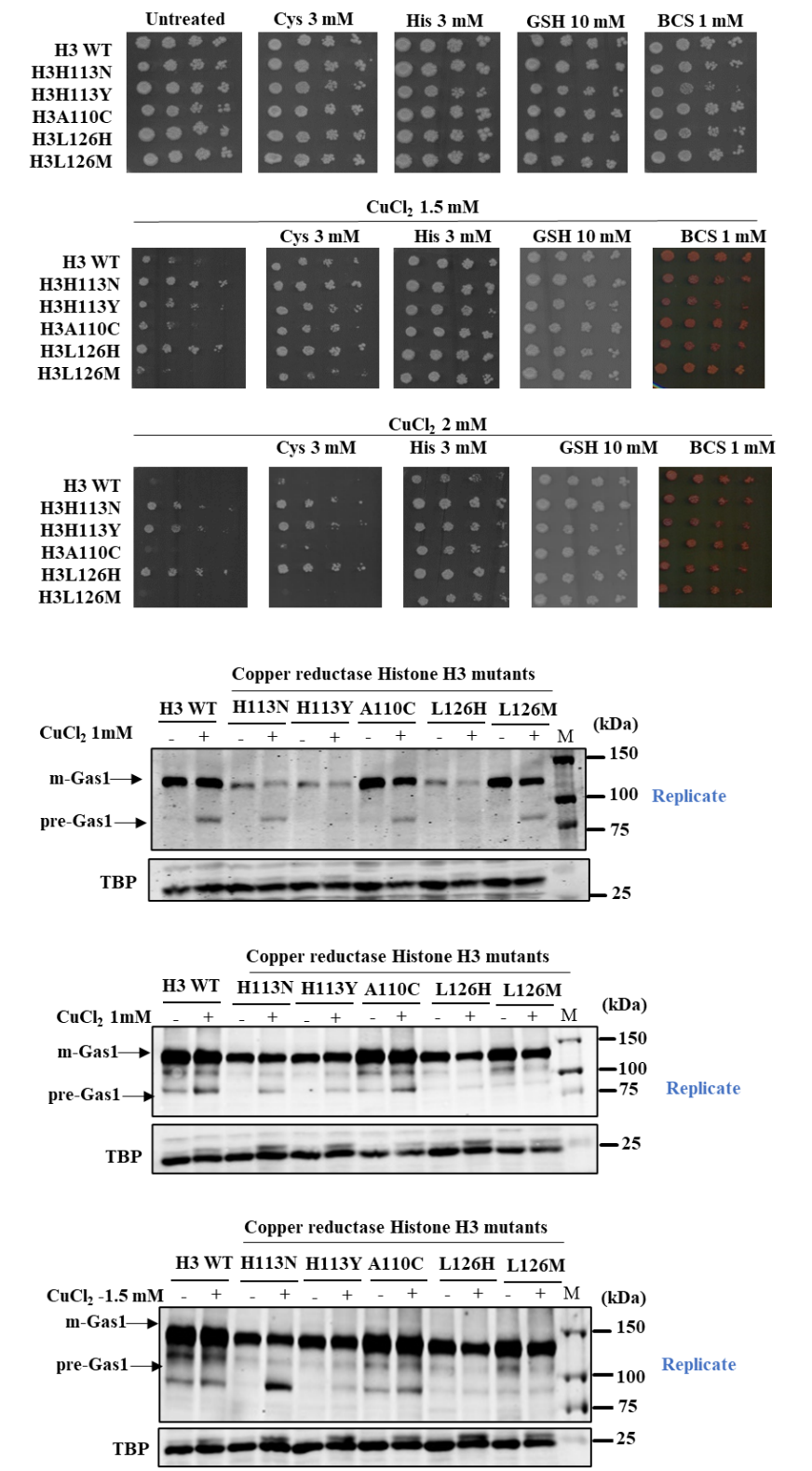


**(B)**

**(A)**

**Figure S27**

**
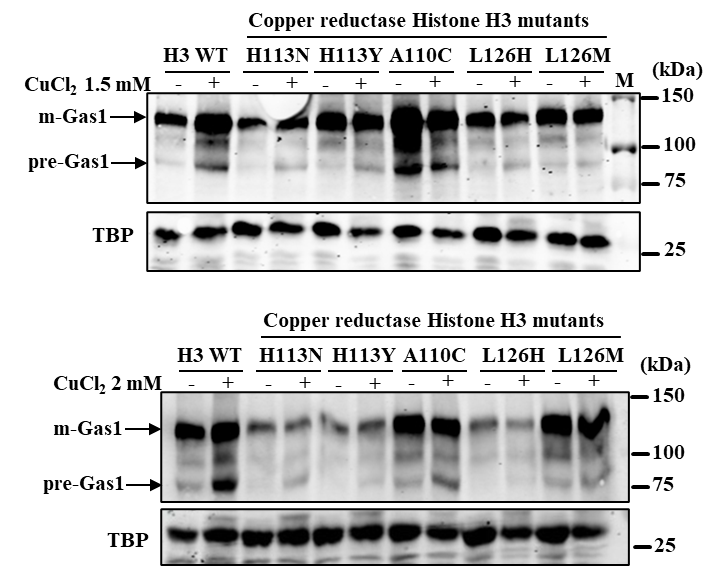
**
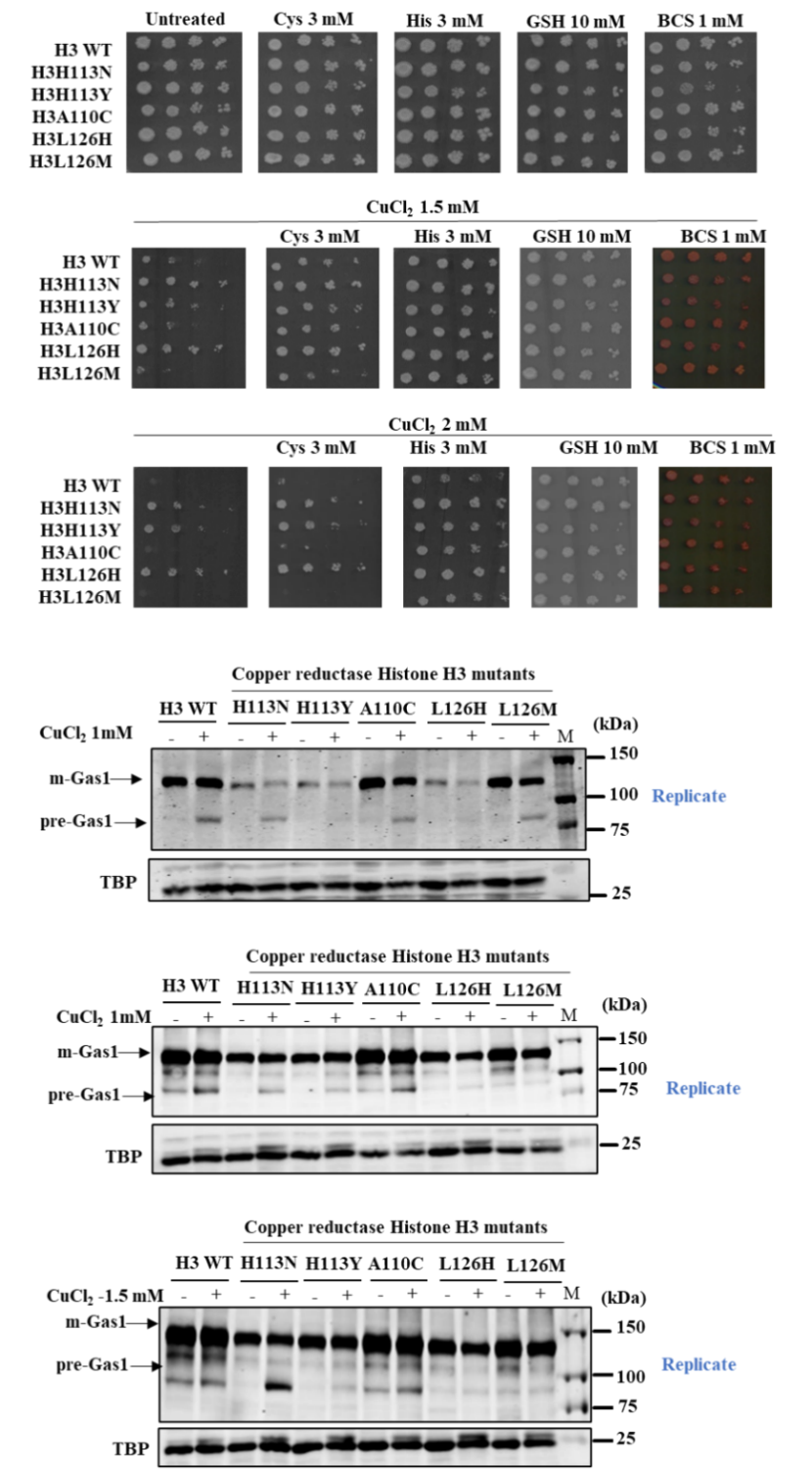


**(E)**

**(C)**

**(F)**

**(D)**

**Figure S27. Analysis of protein translocation in copper reductase histone H3 mutants.** (A) Spot test assay of Gas1 transformed copper reductase histone H3 mutants and wild type cells to test their growth on SC+Agar solid medium in presence of copper, histidine, cysteine, glutathione and co-treatments as indicated. The 10-fold serially diluted cells spotted from left to right on SC+Agar plates containing different concentrations of copper and co-treatments as indicated. UT means copper untreated, growth of cells in normal SC liquid media. (B-F) Immunoblot of Gas1 in copper reductase histone mutants treated with CuCl_2_.2H_2_O (1, 1.5 and 2 mM) for 2 hours in SC liquid growth media. TBP western blotting served as protein loading control.


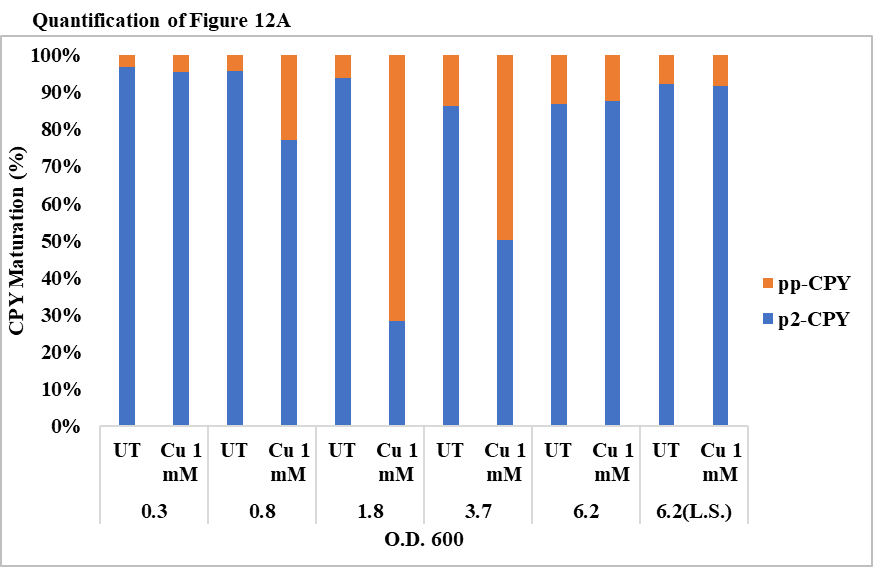


**(A)**

**Figure S28**

**Figure S28.** **Quantification of Gas1 and CPY secretary proteins in wild type yeast cells.** (A) Quantification of the western blots presented in Figure 12. Data indicates the ratio of precursor and mature forms of Gas1 and the ratio of p2 and pp forms of CPY in wild type cells.

**(A)**

**Figure S29**

**
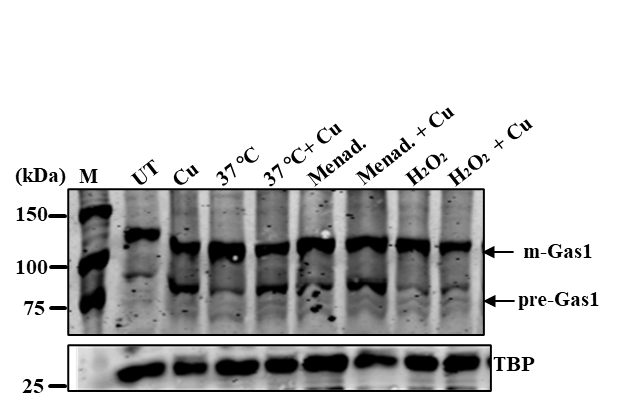
**
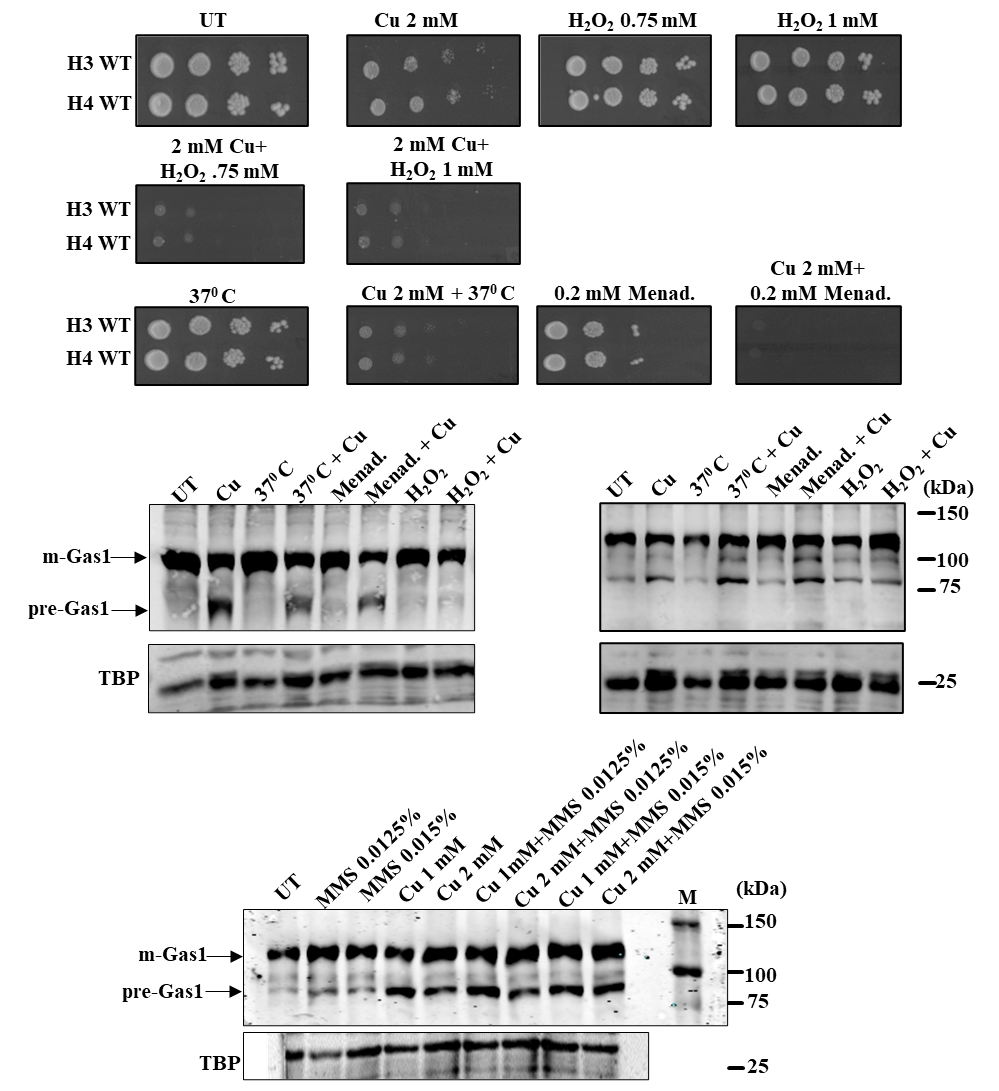


**(B)**


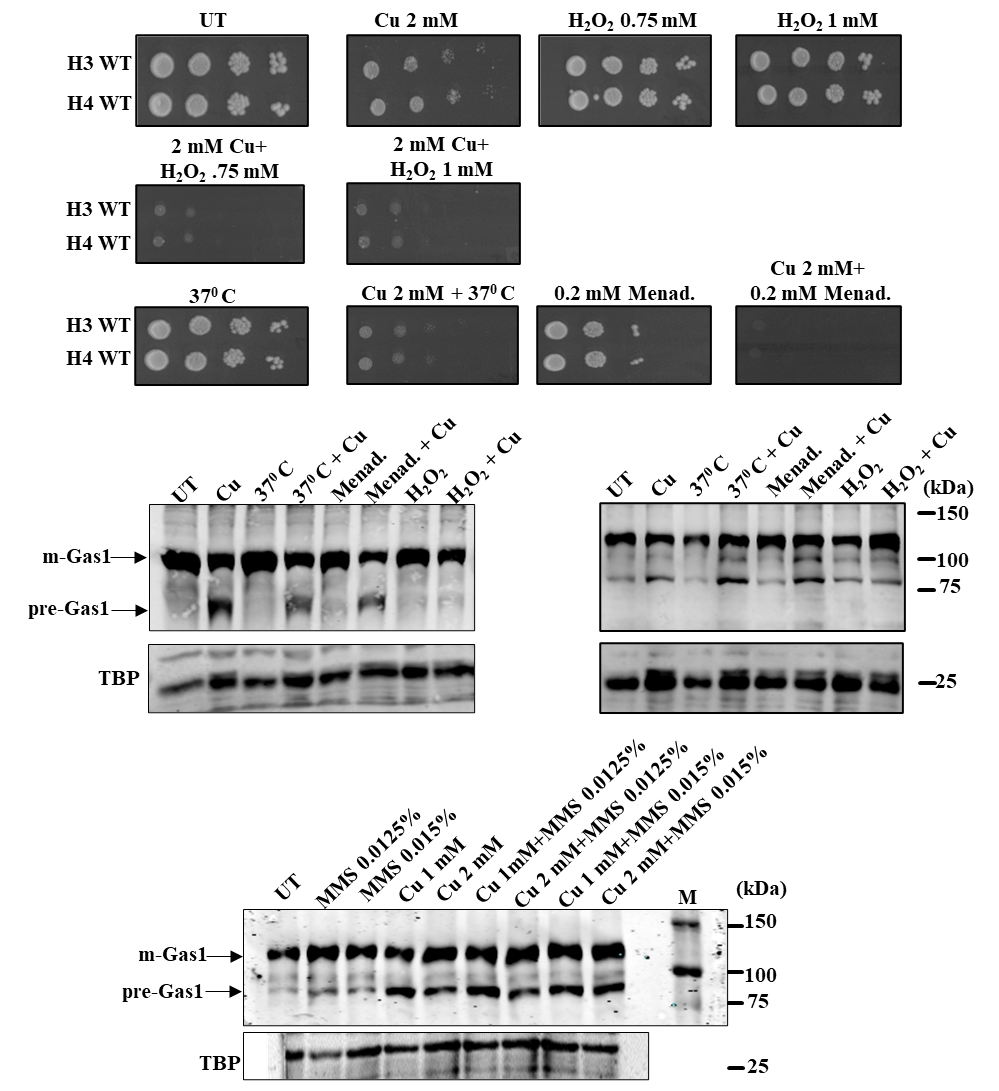


**(D)**

**(C)**

**(E)**

**Figure S29. Effect of stress conditions on copper-induced protein translocation defects:** (A) Spot test assay of wild type yeast cells to test their growth on SC+Agar solid medium in petri dishes. 10-fold serially diluted cells spotted from left to right on SC+Agar plates subjected to different stress conditions (temperature, menadione or H_2_O_2_) as indicated. UT means untreated, growth of cells in normal SC media. (B-D) Immunoblots of Gas1-GFP transformed wild-type cells treated with CuCl_2_.2H_2_O (2 mM) for 2 hours and co-treated with menadione (0.1 mM) or H_2_O_2_ (0.75. (E) Immunoblots of Gas1-GFP transformed wild type treated with CuCl_2_.2H_2_O (1 mM and 2 mM), MMS (0.0125% and 0.0150%), and co-treated as shown. Western blotting with TBP served as a protein loading control.

**(C)**

**(A)**

**Figure S30**

**(D)**

Replicate

**(E)**

**(B)**

**(F)**

**Figure S30. Effect of non-fermentative carbon sources on the copper induced protein translocation defects:** (A) Spot test assay of wild-type cells to test their growth in solid medium (SC+Agar) containing different carbon sources (E; ethanol, G; glycerol) as indicated. 10-fold serially diluted cells were spotted from left to right. UT means copper untreated growth of cells in normal media. (B) Immunoblot of CPY in wild-type cells treated with different concentrations of CuCl_2_.2H_2_O in mM (0.5, 1.0 and 1.5) for 2 hours in SC media containing different carbon sources. Western blotting with TBP served as a protein loading control. SC means synthetic complete media containing 2% glucose, SC(E) means SC media supplemented with 2% ethanol instead of glucose, SC(G) means SC media supplemented with 2% glycerol instead of glucose, and SC(G+E) means SC media supplemented with 2% glycerol and 2% ethanol instead of glucose. (C-D) Semi-quantitative analysis of *FBP1* and *CAT8* expression in wild type cells harvested at different growth (OD_600_ 0.2 – 6.2) phases starting from initial lag-late stationary. *FBP1* and *CAT8* transcripts were normalized to *HHO1* (histone H1) levels. (E-F) Quantification of ‘C and D’ PCR products resolved through 1.5% agarose gel. Data represents means and standard deviation of two independent biological repeats (n=2).

**Table S1:**

1. **List of plasmids used in this study**

| **Plasmid name** | **References** |
| --- | --- |
| pFA-6a-13myc | This study |
| pRS415-Gas1-GFP | (Ha et al., 2014) |
| pRS413-Gas1-GFP | (Ha et al., 2014) |
| pAG425GPD-CUP1-6His | (Yoshikawa et al., 2023) |
| pAG425GPD-ccdB | Addgene |
| pRS426GPD | (Junne et al., 2015) |
| pRS426GPD-DAP2-HA | (Junne et al., 2015) |
| pRS426GPD-Suc2-HA | (Junne et al., 2015) |
| pRS426GPD-H1-HA | (Junne et al., 2015) |

1. **List of primers used in this study:**

| **Gene** | **Primer sequence(5’-3’)** | **References** |
| --- | --- | --- |
| CPY-C term-Myc Tagging | FP:AAGTATGGTTAACGAATGGATCCACGGTGGTTTCTCCTTACGGATCCCCGGGTTAATTAA | This study |
|  | RP:GTAGCTGATAATAAAAACGGTATGCCTACACATACACGCTGAATTCGAGCTCGTTTAAAC | This study |
| *ACT1* | FP: TCGTCGGTAGACCAAGACAC | This study |
|  | RP: TTCTTCTGGGGCAACTCTCA | This study |
| *CAT8* | FP: TATCCAAGGGGGAGAACGCA | This study |
|  | RP: ATTGGCATCCGTGGCATCTG | This study |
| *CUP1* | FP: CCAATGCCAATGTGGTAGCTG | This study |
|  | RP: CATTTCCCAGAGCAGCATGAC | This study |
| *HHO1* | FP: AGCCTGCAACCAGCAAAGG | This study |
|  | RP: GGATCCGACGATCGGGTAGT | This study |
| *FBP1* | FP: CGCCTCAAAAGGCCATCTACT | This study |
|  | RP: GTCGCAAGGGTATGCGAAAAG | This study |

FP: forward primer, RP: Reverse primer

1. **List of strains used:**

| **Name of Strain** | **Genotype** | **References** |
| --- | --- | --- |
| H3 WT | *MATa his3Δ200 leu2Δ0 lys2Δ0 trp1Δ63 ura3Δ0 met15Δ0 can1::MFA1pr-HIS3 hht1-hhf1::NatMX4 hht2-hhf2::[HHTS-HHFS]*-URA3* | Dharmacon |
| H3Δ(13-16) | Isogenic to H3 WT | Dharmacon |
| H3Δ(13-28) | Isogenic to H3 WT | Dharmacon |
| H3Δ(28-31) | Isogenic to H3 WT | Dharmacon |
| H3Δ(29-32) | Isogenic to H3 WT | Dharmacon |
| H3Δ(4-35) | Isogenic to H3 WT | Dharmacon |
| H3K23Q | Isogenic to H3 WT | Dharmacon |
| H3K27R | Isogenic to H3 WT | Dharmacon |
| H3K36Q | Isogenic to H3 WT | Dharmacon |
| H4 WT | *MATa his3Δ200 leu2Δ0 lys2Δ0 trp1Δ63 ura3Δ0 met15Δ0 can1::MFA1pr-HIS3 hht1-hhf1::NatMX4 hht2-hhf2::[HHTS-HHFS]*-URA3* | Dharmacon |
| H4S1D | Isogenic to H4 WT | Dharmacon |
| H4K5R | Isogenic to H4 WT | Dharmacon |
| H4Δ(9-20) | Isogenic to H4 WT | Dharmacon |
| H4Δ(13-24) | Isogenic to H4 WT | Dharmacon |
| BY4741 | *MATa his3Δ1 leu2Δ met15Δ ura3Δ0* | Open Biosystem |
| H2AΔ(1-20) | *MATa his3-1 leu2-0 met15-0 ura3-0 hht1-hhf1::KAN hhf-2hht2::NAT hta1-htb1::HPH hta2-htb2::NAT*  *p[CEN LEU2 hta1Δ(1–20)-HTB1-HHT2-HHF2]* | (Kim et al., 2012) |
| H2AΔ(1-20) H3K4A  {Schmitt, 1990 #1}{Schmitt, 1990 #3} | *MATa his3-1 leu2-0 met15-0 ura3-0 hht1-hhf1::KAN hhf-2hht2::NAT hta1-htb1::HPH hta2-htb2::NAT*  *p[CEN LEU2 hta1Δ(1–20)-HTB1-hht2K4A-HHF2]* | (Kim et al., 2012) |
| H2AΔ(1-20) HR2A | *MATa his3-1 leu2-0 met15-0 ura3-0 hht1-hhf1::KAN hhf-2hht2::NAT hta1-htb1::HPH hta2-htb2::NAT*  *p[CEN LEU2 hta1Δ(1–20)-HTB1-hht2R2A-HHF2]* | (Kim et al., 2012) |
| H3 WT | *MATa his3Δ1 leu2Δ0 met15Δ0 ura3Δ0 (hht2Δ::URA)Δ::HHT2* | (Attar et al., 2020) |
| H3H113N | *MATa his3Δ1 leu2Δ0 met15Δ0 ura3Δ0 (hht2Δ::URA)Δ::hht2-H113N hht1-H113N* | (Attar et al., 2020) |
| H3H113Y | *MATa his3Δ1 leu2Δ0 met15Δ0 ura3Δ0 (hht2Δ::URA)Δ::hht2-H113Y hht1-H113Y* | (Attar et al., 2020) |
| H3A110C | *MATa his3Δ1 leu2Δ0 met15Δ0 ura3Δ0 (hht2Δ::URA)Δ::hht2-A110C hht1-A110C* | (Attar et al., 2020) |
| H3L126H | *MATa his3Δ1 leu2Δ0 met15Δ0 ura3Δ0 (hht2Δ::URA)Δ::HHT2 hht2-L126H hht1-L126H* | (Tod et al., 2024) |
| H3L126M | *MATa his3Δ1 leu2Δ0 met15Δ0 ura3Δ0 (hht2Δ::URA)Δ::HHT2 hht2-L126M hht1-L126M* | (Tod et al., 2024) |

**References:**

Attar, N., Campos, O. A., Vogelauer, M., Cheng, C., Xue, Y., Schmollinger, S., Salwinski, L., Mallipeddi, N. V, Boone, B. A., Yen, L., Yang, S., Zikovich, S., Dardine, J., Carey, M. F., Merchant, S. S., & Kurdistani, S. K. (n.d.). *The histone H3-H4 tetramer is a copper reductase enzyme*. https://www.science.org

Ha, C. W., Kim, K., Chang, Y. J., Kim, B., & Huh, W. K. (2014). The β-1,3-glucanosyltransferase Gas1 regulates Sir2-mediated rDNA stability in Saccharomyces cerevisiae. *Nucleic Acids Research*, *42*(13), 8486–8499. https://doi.org/10.1093/nar/gku570

Junne, T., Wong, J., Studer, C., Aust, T., Bauer, B. W., Beibel, M., Bhullar, B., Bruccoleri, R., Eichenberger, J., Estoppey, D., Hartmann, N., Knapp, B., Krastel, P., Melin, N., Oakeley, E. J., Oberer, L., Riedl, R., Roma, G., Schuierer, S., … Hoepfner, D. (2015). Decatransin, a new natural product inhibiting protein translocation at the Sec61/SecYEG translocon. *Journal of Cell Science*, *128*(6), 1217–1229. https://doi.org/10.1242/jcs.165746

Kim, J. A., Hsu, J. Y., Smith, M. M., & Allis, C. D. (2012). Mutagenesis of pairwise combinations of histone amino-terminal tails reveals functional redundancy in budding yeast. *Proceedings of the National Academy of Sciences of the United States of America*, *109*(15), 5779–5784. https://doi.org/10.1073/pnas.1203453109

Tod, N. P., Vogelauer, M., Cheng, C., Karimian, A., Schmollinger, S., Camacho, D., & Kurdistani, S. K. (2024). The role of histone H3 leucine 126 in fine-tuning the copper reductase activity of nucleosomes. *Journal of Biological Chemistry*, *300*(6). https://doi.org/10.1016/j.jbc.2024.107314

Yoshikawa, Y., Nasuno, R., Takaya, N., & Takagi, H. (2023). Metallothionein Cup1 attenuates nitrosative stress in the yeast Saccharomyces cerevisiae. *Microbial Cell*, *10*(8), 170–177. https://doi.org/10.15698/mic2023.08.802
